# Health Associations of Positive Childhood Experiences: A Scoping Review of the Literature

**DOI:** 10.3390/ijerph22010059

**Published:** 2025-01-03

**Authors:** Joachim Hero, Laura Gallant, Dina Burstein, Sydne Newberry, Nabeel Qureshi, Katie Feistel, Kayla N. Anderson, Kelsey Hannan, Robert Sege

**Affiliations:** 1 RAND Corporation, Santa Monica, CA 90401, USA; jhero@rand.org (J.H.); sydnen@rand.org (S.N.);; 2 Center for Community-Engaged Medicine, Institute for Clinical Research and Health Policy Studies, Tufts Medical Center, Boston, MA 02111, USA; laura.a.gallant@tuftsmedicine.org (L.G.); kelsey.hannan@tuftsmedicalcenter.org (K.H.); robert.sege@tuftsmedicine.org (R.S.); 3 Centers for Disease Control and Prevention, National Center for Injury Prevention and Control, Atlanta, GA 30333, USA; lxx7@cdc.gov

**Keywords:** positive childhood experiences, health outcomes, adverse childhood experiences, child development, substance use disorder, suicidal behavior, mental health

## Abstract

We report the results of a scoping review of the literature investigating associations between positive childhood experiences (PCEs) and selected health outcomes to identify which have the highest level of research activity based on the indexed academic literature. Yielded articles underwent title/abstract (Ti/Ab) and full text screening utilizing inclusion/exclusion criteria. The review was guided by PCE categories from the Healthy Outcomes from Positive Experiences framework: relationships, environment, engagement, and emotional growth. The initial search yielded 8,919 unduplicated articles, 759 were retained following Ti/Ab review and 220 articles were retained after full text screening describing 795 tested associations across 23 PCE types in ten outcome categories. The outcomes most commonly examined were substance misuse (305 tested associations across 93 studies), suicidal behaviors (195 tested associations across 56 studies), and depression (112 tested associations across 55 studies). Physical health outcomes were less common (14 tested associations across six studies). Of the PCE exposures, relationships represented 415 of tested associations, 236 with environment, and 114 with social engagement. A significant body of research demonstrated associations between PCEs and health outcomes. While further research is needed, available research suggests that public health efforts to promote PCEs may have impact across multiple domains.

## 1. Introduction

### 1.1. Rationale

Positive and adverse childhood experiences affect child and adult health outcomes. The detrimental effects of adverse childhood experiences (ACEs) have been well documented [1,2,3,4]. Recent research has demonstrated the beneficial and protective effect of positive childhood experiences (PCEs) on health outcomes [5,6,7,8,9,10,11]. The childhood exposures that contribute to positive developmental and health outcomes have been referred to as PCEs [5,12], childhood protective factors [13,14], benevolent childhood experiences [15], and counter-ACEs [7].

Evidence has emerged demonstrating that PCEs are associated with beneficial long-term health outcomes. This includes protecting against mental health conditions, suicidal behaviors [5,7,9,16,17], and reducing risk for cigarette use and alcohol quantity consumed [10]. PCEs have also been associated with a lower risk of reporting any adult physical health condition [17], improved heart health at middle age [18], and greater adherence to health-promoting diets [7]. PCEs contribute to family health, potentially mitigating the effects of ACEs [8]. In addition to physical health, PCEs have also been found to contribute to positive social outcomes, like higher educational and income attainment [19].

To date, a common understanding of how to measure and define PCEs has not coalesced, and no consensus framework exists for conceptualizing and categorizing the universe of potential PCEs. This has contributed to significant variation in research approach and terminology, and, consequently, the scope of the literature on these topics is poorly understood. Several reviews looking at the body of research describing the health effects of ACEs have been conducted [20,21,22,23,24,25], however a comprehensive scoping review on research investigating the effects of PCEs on health outcomes has not been published.

### 1.2. Objectives

The objective of this review was to identify potential PCEs in the published literature, the associations observed between specific PCEs and selected health outcomes, and which among these specific PCEs have the highest level of research activity. Potential PCEs refers to experiences during childhood that may contribute to positive outcomes in terms of health and well-being. These experiences may help promote resilience, physical, mental and behavioral health, as well as optimal development. They are hypothesized to have beneficial effects, and this review aims to explore the existing evidence and associations in the literature to better understand their impact. Although there is a broad array of potential outcomes, this review focused on a selected set of high priority outcomes.

## 2. Methods

We conducted a scoping review of the literature investigating associations between PCEs and the selected health outcomes. The goal of a scoping review is to map the key concepts, theories, evidence, or research gaps in a particular field or topic. As a result of this review, we sought to identify research trends, categorize existing studies, and highlight areas where further investigation is needed, providing a comprehensive overview of the field. All PRISMA (Preferred Reporting Items for Systematic Reviews and Meta-Analyses) guidelines were followed (File S1).

### 2.1. Positive Childhood Experiences

**Figure 1:**
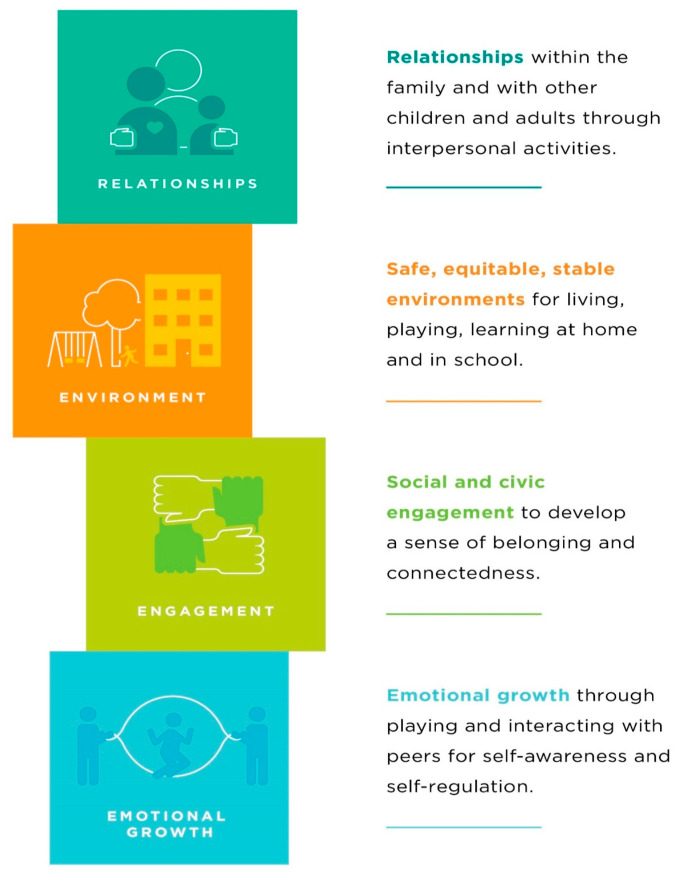
The four building blocks of HOPE. SOURCE: HOPE—Healthy Outcomes from Positive Experiences. Available online: https://positiveexperience.org/ (accessed on 1 November 2024).

This review used the HOPE (Healthy Outcomes from Positive Experiences) framework to define PCEs [26]. The HOPE framework identifies four key domains of PCEs that function to create thriving, resilient children, even in the face of adversity [17,27,28]: secure, nurturing relationships with adults and other children (henceforth known as “relationships”); safe, equitable, and stable environments to live, learn and play (“environment”); social and civic engagement (“social engagement”); and opportunities for emotional growth (“emotional growth”) (Figure 1) [26]. These domains cover a wide variety of potential PCEs; therefore, we organized PCEs into four overarching categories (relationships, environment, social engagement, and emotional growth) based respectively on the HOPE domains. We did not ultimately use emotional growth as an exposure to PCEs due to limitations in search tools, the difficulty in operationalizing, and the potential for it to be confused as a health outcome rather than an exposure.

The scope of the review included published studies on a wide variety of potential PCEs. We considered a potential PCE to be an exposure (1) experienced in childhood (younger than 18), (2) that was framed and coded as a beneficial factor, and (3) that is not simply the inverse or absence of negative experiences or harmful conditions. We did not include exposures representing the fulfillment of basic needs (e.g., adequate food or shelter) or interventions aimed at addressing prior trauma in symptomatic individuals.

Our approach was based on the widely adopted Arksey and O’Malley scoping review framework [29], which includes the five stages of identifying the research question, identifying relevant studies, study selection, charting the data, and collating, summarizing, and reporting the results. A detailed research protocol was developed and pre-registered using Open Science Framework (DOI: https://doi.org/10.17605/OSF.IO/PR8ZY (accessed on 15 November 2024)) [30].

### 2.2. Health Outcomes

Grounded by the Centers for Disease Control and Prevention’s (CDC) approach to studying the effects of childhood experiences [31,32], this study included outcomes related to four significant contributors to leading causes of death, disease, and limited well-being across the lifespan: (1) physical health conditions (e.g., cardiovascular disease, diabetes, infectious diseases); (2) mental health conditions (e.g., depression or anxiety); (3) other selected behavioral health outcomes (e.g., suicidal behaviors or substance misuse); and (4) violence victimization or perpetration. This approach ensured the review focused on outcomes that could have the greatest potential to inform policy and improve health outcomes.

### 2.3. Inclusion/Exclusion Criteria

The inclusion/exclusion (I/E) criteria were designed to identify studies that would allow us to address the key questions by defining the parameters for conducting the literature search. I/E criteria were developed through discussions with subject matter experts. The I/E criteria were developed using a predefined framework: Population, Exposure (PCE), Comparator, Outcome, Timing, Setting, Study Design. These criteria were used to design the literature searches and screen the search outputs, to ensure transparency of the review. Included studies were limited to those whose purpose was to assess relationships between individual or combined PCEs, or the moderating effect of these on ACEs, and associations with health outcomes. Other specific inclusion criteria were: (1) studies had to be of PCEs that occurred after age 1 and before age 18, (2) studies had to include a comparison group (those without exposure to the PCE), and (3) studies had to include the specific outcomes described above. Studies looking at exposures to PCEs prior to the first birthday were excluded. There were no restrictions on the length of follow-up. Studies of non-U.S. populations, purely qualitative studies, and non-research studies were excluded. Non-English language publications, unpublished research, and grey literature (e.g., studies not indexed in academic databases or that had not undergone peer-review) were excluded. In-scope systematic reviews and meta-analyses are not described in this scoping review, though they were identified as part of the search criteria and may be analyzed in the future. Detailed I/E criteria are available in File S2.

Screening and abstraction were conducted using DistillerSR software version 2.35 [33]. DistillerSR is a widely used web-based software tool designed for managing and conducting systematic reviews. This platform was chosen because it facilitates the screening of articles based on predefined inclusion and exclusion criteria, allowing multiple reviewers to evaluate abstracts and full texts in a systematic and organized way.

### 2.4. Search Parameters and Study Selection

**Figure 2:**
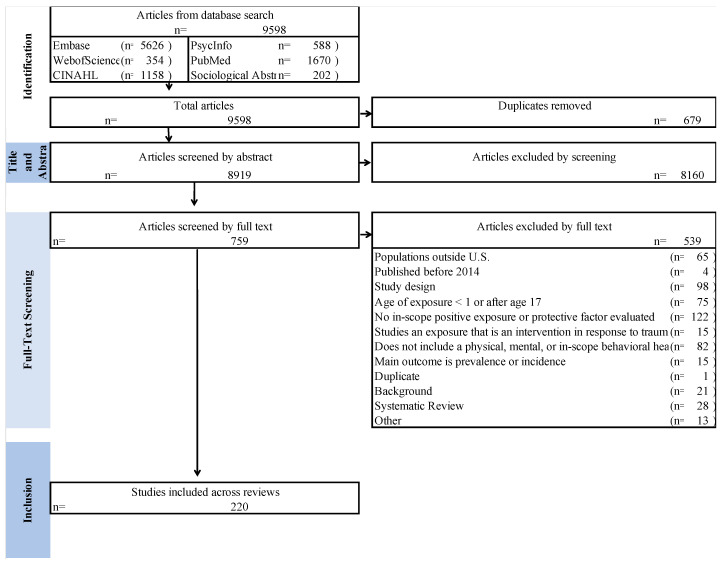
PRISMA Chart for Positive Childhood Experiences and Outcomes Search. SOURCE: RAND analysis of search results and screening data. NOTE: This PRISMA diagram shows the number of articles identified in our search by database, the number of articles that remained after automated de-duplication, and the number of articles excluded at each screening stage. Articles screened by full text include those excluded upon further review during abstraction. Systematic reviews and meta-analyses were excluded from scoping review results but retained for future analysis.

We conducted a broad-based search of the indexed academic literature clustering search terms by exposure to specific PCEs within the three categories of relationship, environment, social engagement, and the four health-related outcomes categories. PRISMA standards were followed for all searches [34]. PRISMA consists of a set of guidelines designed to help researchers transparently and comprehensively report scoping and systematic reviews and meta-analyses, ensuring consistency and quality in the reporting of evidence synthesis (Figure 2).

Keywords and controlled vocabulary were reviewed by the project team. Search terms were developed in stages, starting with broader, more general terms and gradually narrowing them down to more specific and precise terms. This iterative process was done using PubMed in order to optimize the search results by progressively adjusting the search terms to maximize the number of relevant publications included in the review or study.

Nested search strings were added to terms generating poorly targeted search results. Search terms were adapted for—and applied to—six databases: PubMed, CINAHL, Embase, Sociological Abstracts, PsycInfo, and Web of Science. Searches were limited to English language studies that focused on populations within the United States, published between 1 January 2014 and 6 April 2022. This approach aligned with the scope of the organizations’ research objectives and funding priorities. Additionally, limiting the search in this way helped keep the scope manageable within the time and resources available for the project. The final search strategy and results are provided in File S3.

Citations and abstracts identified in the literature searches were exported to a reference library manager, duplicates were removed, and the remaining citations were uploaded to DistillerSR for Ti/Ab screening. Using the I/E criteria described above, pairs of screeners comprising an experienced reviewer and a graduate student independently screened groups of 30 titles and abstracts of studies identified in the searches in practice rounds until all reviewers agreed on interpretation and application of the I/E criteria. Articles passing the Ti/Ab screen were screened using the full text of the article. Full-text screening was conducted using a form reflecting the I/E criteria. Reasons for exclusion were recorded (Figure 2). Full text screening was conducted independently in duplicate. Conflicts were resolved through senior investigator review. A minimum of three rounds were conducted for both Ti/Ab and full text screening. Weighted overall Kappa for Ti/Ab screening was 0.92 and 0.81 for full text screening.

### 2.5. Data Abstraction

A data abstraction form captured study-level data concerning the study population (including sample size, composition of the study population [e.g., age, gender, race/ethnicity, sexual or gender identity, and whether prior ACEs were captured, if known]), exposures (i.e., PCE type, definition, when and how it was measured), types of health outcomes (i.e., when and how they were assessed or measured), timeframe (i.e., years during which the study was conducted), geographic location(s) of the study, study design, and if the study implemented a previously published survey instrument. Data were abstracted by a single reviewer and cross-checked by a second reviewer. Discrepancies were reconciled with the help of a senior investigator if necessary. We also captured the authors’ assessments of whether and how each exposure to a PCE was statistically associated with an outcome of interest.

### 2.6. Analysis

#### 2.6.1. PCE Categories

We categorized all specific PCE exposures reported in studies into initial PCE types based on the HOPE framework [26]. New sub-categories were created for similar positive experiences when they were observed in three or more studies and could not be placed in a priori categories. These new sub-categories were created iteratively through group discussion. For example, several studies examined the positive impacts of “familism”, a cultural value emphasizing priority of family, which the research team had not included among its a priori categories. Experiences identified in our search that did not fit within a priori categories and were observed in fewer than three studies (e.g., relationships with pets, inter-parent or caregiver relationship functioning) were put into a miscellaneous category labeled “other”.

#### 2.6.2. Outcomes

The study team, in collaboration with subject matter experts at the CDC, carefully selected high priority outcomes from a wide range of potential outcomes. This selection process involved evaluating the relevance and impact of each outcome in relation to public health priorities. The team and CDC experts used their combined expertise to identify outcomes that were most likely to provide meaningful insights into the effects of PCEs, ensuring the focus was on outcomes that would have the greatest potential to inform policy and improve health outcomes.

Each outcome tested within a study was categorized as either: mental health (depression, anxiety, post-traumatic stress disorder [PTSD], other mental health), other selected behavioral health (substance misuse, suicidal behaviors [e.g., ideation, attempts]), violence victimization or perpetration, or physical health conditions (cardiovascular disease [CVD], other physical health). Results were analyzed at the level of the PCE-outcome pair, including the number of tested associations across studies (also identifying the number of studies); the distribution of study designs by tested association (cross-sectional, cohort, or other design); the kinds of outcomes examined; and the reported results by whether no association, or a beneficial or harmful association was detected. We coded consistently in favor of a beneficial association if any beneficial association was found; if an association was tested at multiple time-points, we coded a beneficial association if one was detected at any time-point.

#### 2.6.3. Level of Research Activity

We characterized the *level of research activity* for each PCE both by outcome and across all outcomes. To determine level of research activity, PCE-outcome pairs were assigned points based upon whether they had at least 10 studies, 5–9 studies, or fewer than 5 studies; based on the mean sample sizes (greater than 1000, 500–999, or 499 or less); and whether they employed cross-sectional or longitudinal study designs.

A “high” level of research activity designation was given to investigated PCE-outcome pairs with 10 or more studies, including at least 3 cohort and 3 cross-sectional studies (to prioritize areas with more robust research designs), and with average sample size of greater than 1000. Investigated PCE-outcome pair associations that did not meet all of these thresholds but had at least five studies or average sample size greater than or equal to 500, were given a “moderate” level of research activity. A “low” level of research activity was assigned to all other investigated associations supported by at least one study. An absolute measure of research activity was used to reflect any skew in the distribution and consistently represent areas where very few studies have been conducted.

This scoping review did not evaluate the quality of the evidence or assess the risk of bias based on study factors such as sample characteristics, study design, or analytical rigor.

## 3. Results

### 3.1. Summary of Evidence

The initial search yielded 8919 unduplicated articles. Of these, 8160 were excluded based on Ti/Ab review. After full-text screening, 220 articles were retained for inclusion in the review. The most common reasons for exclusion were that the study had an out-of-scope study design, health outcome, or age of exposure to the PCE (Figure 2). The complete list of included literature is available in File S4. A complete summary of all included literature including study type, data source, outcome category tested, and study size can be seen in File S5.

### 3.2. PCE Exposure/Outcome Associations in the Literature

We identified and characterized 795 tested associations between specific PCEs and outcomes in the 220 included studies. We tested the association between 23 PCE types and ten outcome categories. The outcomes most commonly examined in our search were substance misuse (305 tested associations across 93 studies), suicidal behaviors (195 tested associations across 56 studies), and depression (112 tested associations across 55 studies). Physical health outcomes were far less common (14 tested associations across six studies). The HOPE framework building block of Relationship had the largest number of tested associations (n = 415), followed by Environment (n = 236), and then Social Engagement (n = 114). Several PCE sub-categories within these building blocks had over 20 tested associations. For the relationship category, this included the quality of relationship with a caregiver, love and support from a caregiver, support from a non-caregiver adult (excluding teachers), having positive peer relationships, and support from a teacher. The environment category included having access to a positive school environment, engaging in regular physical activity, and parental monitoring and related family environments. The engagement category included opportunities for extracurricular engagement with school or community, having beliefs that give comfort, and having a sense of community or cultural belonging.

### 3.3. Level of Research Activity on PCE Exposure/Outcome Associations

Across all included outcomes, ten PCE sub-categories had a high level of research activity, five had a moderate level of research activity, and nine had a low level of research activity. Within the relationship PCEs, love and support from a caregiver, quality relationship with a caregiver, having positive peer relationships, social support, support from a teacher, and support from a non-caregiver adult (excluding teachers) all had high levels of research activity. Within the environment PCEs, having access to a protective school environment and parental monitoring and related family environments both had high level of research activity. Under social engagement, a high level of research activity was identified for research related to having opportunities for extracurricular engagement with school or with the community and having beliefs that give comfort (e.g., attendance at place of worship) (Table 1).

### 3.4. Beneficial Associations Between PCE Exposure and Outcomes

Table 2, Table 3 and Table 4 show the proportion of tested associations between a PCE type and an included outcome that were reported to be beneficial and statistically significant. A cell showing 50% would indicate that half of all tested associations between a given PCE and outcome showed a statistically significant beneficial association, whereas the other half either did not detect an association or detected a harmful association (harmful associations were rare and no PCE type had a majority of associations that were harmful). Below each proportion, we show the total number of associations that were tested for a PCE-outcome pair, followed by the mean analytic sample size reported among studies that found a beneficial association. We also highlight areas where we found a high level of research activity, which may correspond to a stronger basis of evidence. Below we present results from those outcomes with the highest research activity. A summary of the percent of all associations tested showing a beneficial association between a PCE and a health outcome can be seen in File S6.

Relationships. We recorded a high proportion of beneficial associations between love and support from a caregiver and indicators of suicidal behaviors (74 percent) as well as mental health outcomes (65 percent for depression, 82 percent for other mental health outcomes). A minority of studies detected a beneficial association with substance misuse (41 percent), though sample sizes were lower than for other outcomes. A similar pattern was found for high-quality relationships with a caregiver. We recorded fewer beneficial associations with non-caregiver sources of support, including friends, teachers, and other non-caregiver adults. A broad summary measure of “social support,” which captured support provided to the child from any source, was also found to have a lower proportion of significant results (Table 2).

Environment. We recorded a high proportion of beneficial associations between having access to a protective school environment and substance misuse (81 percent) as well as for included outcomes overall (74 percent). Parental monitoring and related family environments overall had beneficial findings with substance misuse (63 percent), though on a research base with smaller sample sizes. Parental monitoring and related PCEs also were largely found to be beneficial for included outcomes overall (Table 3).

Social engagement. Slightly more than half (56 percent) of tested associations for having opportunities for extracurricular engagement with school or community were recorded to be beneficial for all outcomes. A similar proportion (53 percent) of tested associations for having beliefs that give comfort were found to be beneficial for all outcomes (Table 4).

Combinations of PCEs. In a small set of articles, PCEs were not individually tested but examined for their cumulative effect on health outcomes. We identified 15 tested associations across 9 studies (seven cross-sectional studies, and two cohort studies) of cumulative PCEs measures and their association with in-scope outcomes. Among these studies, 80% of tested associations (12 of 15 associations tested with a mean sample size of 38,523) of cumulative PCEs found beneficial associations, with tests concentrated in substance misuse and depression.

## 4. Discussion

This scoping review aimed to identify potential PCEs in the existing literature, explore the associations observed between specific PCEs and various health outcomes, determine which of these PCEs has the highest level of research activity, and provide a comprehensive understanding of the state of research on PCEs. This review identified a substantial body of work associating PCEs with improvements in child and adult outcomes. This research base highlights factors that promote health or mitigate the adverse impacts of ACEs. However, despite the extensive research in this field, significant variations exist in the volume of research focused on specific types of PCEs and outcomes.

We found that research activity is most prevalent in the areas of caregiver relationships and access to a positive school environment. Many of the less frequently studied PCEs relate to environmental factors and social engagement and are rooted in structural and social determinants of health (SDoH) [35,36], which shape the conditions in which children grow, live, and play. Enhancing our understanding of how these PCEs affect health and well-being across the lifespan could direct public health attention toward the most impactful PCEs for advancing equity [37].

More than half (63 percent) of the tested associations in the included research concentrated on outcomes related to substance misuse or suicidal behaviors (e.g., ideation, planning, or attempts). Mental health conditions, primarily depression, were the next most common type of outcome studied. Other mental health conditions that lead to significant public health burden, including anxiety and PTSD [38], received less research attention. This review also identified low levels of research activity focused on outcomes related to physical health or violence victimization or perpetration. The low levels of research activity on various mental health conditions, physical health, and violence can have profound consequences for both individuals and society. In mental health, insufficient research limits the development of effective interventions. Similarly, when research is scarce in the domain of physical health, it results in missed opportunities for improving prevention strategies. For violence, particularly intimate partner violence, child abuse, or systemic violence, the absence of robust research impedes the design of preventive measures, accurate reporting mechanisms, and effective policies.

The disparity in research on the relationship between PCEs and mental health, physical health, and violence could be influenced by a number of factors. One major factor that could be impacting which associations get research attention is bias in funding allocation. Most funding exploring the health effects of ACEs is provided by the National Institutes of Health and has focused on mental health and substance use disorders [39]. Our informal reading of the acknowledgement sections of the papers included here suggests a similar funding pattern for PCEs. For example, the National Institutes of Health currently has 28 known active projects with total funding of USD 12,155,575 related to “positive childhood experiences.” Of these, the National Institutes of Mental Health has eight active projects, National Institute of Child Health and Human Development has five, and no other institute has more than three [39]. The NIH prioritizes research on mental health and substance misuse in relation to ACEs, and appears to do the same with PCEs, potentially resulting in the exclusion of important factors.

In addition to bias in funding allocation, the publication of certain research findings could also be biased. For example, studies that did not find associations between PCEs and these outcomes, or those with negative or inconclusive results, may be less likely to be published than those with positive findings. This kind of bias in research publication has been documented [40,41].

Beyond bias in funding and publication, gaps in the literature may also stem from methodological challenges. For example, difficulty in identifying appropriate datasets or devising appropriate and accurate measures for certain outcomes will limit researchers’ ability to conduct high-quality research. Efforts to improve the availability and quality of data on PCEs and their implications for health across the lifespan are necessary to understand and capitalize upon their potential to improve public health and population impact.

### Limitations

As a scoping review, this study has inherent limitations. First, we did not evaluate the quality of the literature or strength of associations. For this reason, our findings cannot inform discussion on the relative impact of different PCEs. Next, categorizing studies as prospective versus retrospective cohort studies proved challenging using published methods. Many studies utilized datasets from large, longitudinal, prospective studies but did not clearly specify whether research questions were formulated prior to or after data had been collected. To avoid miscategorization, we did not differentiate prospective and retrospective cohort studies. This review only considered publications in peer-reviewed, indexed publications. Publication and researcher bias favor positive findings, which could cause bias in this scoping review if investigations with null findings were not published. In addition, the level of research activity may reflect the availability of funding: Gaps in the literature may reflect the need for further study and not necessarily a lack of association. The approach used also focused on negative health outcomes; positive health outcomes were excluded. Finally, the review only collected evidence from 2014–2022, in English, and focused on populations in the United States, which may limit generalizability.

## 5. Conclusions

In summary, this review sought to map the landscape of PCEs in the current literature, assess the connections between specific PCEs and health outcomes, and identify which PCEs have garnered the most attention in terms of research activity. Significant research activity focuses on the health effects of PCEs. However, notable gaps were observed in the research focused on PCEs related to environmental factors and social engagement. Many plausible physical, mental, and behavioral health outcomes stemming from PCEs are similarly understudied. These findings provide a valuable reference for understanding where the most significant gaps and opportunities for future research exist in the field of PCEs. Efforts to better understand the effects of understudied PCEs on an array of outcomes can inform prevention, intervention, and response efforts. Further investigation into underexplored PCEs and the strengthening of research in key areas identified by this review will be critical to advancing the understanding and effectiveness of primary care environments in improving health outcomes. While addressing these gaps in the literature is necessary to improve our understanding of the mechanisms that impact health, public health practice can continue to promote and implement evidence-based strategies that support the development of safe, stable, nurturing relationships and environments [38].

## Figures and Tables

**Table 1:** Level of Research Activity for Positive Childhood Experience type by Health Outcome.

		Substance Misuse	Suicidal Behaviors	Violence Perpetration	Violence Victimization	Anxiety	Depression	Post-Traumatic stress Disorders	Other Mental Health	Cardiovascular Disease	Other Physical Health	Any Outcome
Relationships	Being in nurturing, supportive relationships (combined measures)											
	Being securely attached to a parent or caregiver											
	Doing activities with caregiver											
	Familism and related family environments											
	Having positive peer relationships											
	Having prosocial peers											
	Love and support from a caregiver											
	Quality relationship with caregiver											
	Social Support											
	Support from a teacher											
	Support from non-caregiver adult (excluding teachers)											
Environment	Access to green spaces and playgrounds to play											
	Having access to a protective school environment											
	Living in a safe neighborhood or community											
	Parental monitoring and related family environments											
	Regular physical activity											
Social Engagement	Community connectedness											
	For American Indian or Alaska Native communities, native culture engagement											
	Having opportunities with constructive social engagement and developing connectedness											
	Having opportunity for extracurricular engagement with school or with the community											
	Having opportunity to have beliefs that give comfort											
	Sense of community or other cultural belonging											
	Cumulative measures of PCE											
	Other											

SOURCE: RAND analysis of study data. NOTE: A “high” level of research activity designation was given to investigated PCE-outcome pairs with 10 or more studies, including at least 3 cohort and 3 cross-sectional studies (to prioritize areas with more robust research designs), and with average sample size of greater than 1000. This corresponds to the darkest color cells. Investigated PCE-outcome pair associations that did not meet all of these thresholds but had at least five studies or average sample size greater than or equal to 500, were given a “moderate” level of research activity. This corresponds to the moderate color cells. A “low” level of research activity was assigned to all other investigated associations supported by at least one study. This corresponds to the light color cells. Blank indicates no research was found.

**Table 2:** Percent of tests showing a beneficial association between a relationship-focused positive childhood experience and a health outcome.

		Substance Misuse	Suicidal Behaviors	Violence Perpetration	Depression	Other Mental Health	Cardiovascular Disease	Other Physical Health	Any Outcome
		% of associations where beneficial relationship detected (Total # of tests, mean sample size studies where beneficial association was detected)							
Relationships	Being in nurturing, supportive relationships (combined measures)	100% (2, 3414)	0% (0, 0)	0% (0, 0)	0% (0, 0)	100% (1, 357)	0% (0, 0)	0% (0, 0)	100% (3, 2395)
	Being securely attached to a parent or caregiver	100% (4, 1779)	0% (1, 0)	0% (0, 0)	100% (2, 12,248)	0% (0, 0)	0% (0, 0)	0% (0, 0)	86% (7, 5269)
	Doing activities with caregiver	0% (0, 0)	0% (0, 0)	0% (0, 0)	100% (1, 12,248)	0% (0, 0)	0% (0, 0)	0% (0, 0)	100% (1, 12,248)
	Familism and related family environments	75% (4, 251)	0% (0, 0)	0% (0, 0)	0% (3, 0)	0% (0, 0)	0% (0, 0)	0% (0, 0)	33% (9, 251)
	Having positive peer relationships	10% (10, 2168)	50% (20, 20,501)	20% (5, 18,451)	38% (8, 5914)	80% (5, 34,162)	0% (0, 0)	0% (0, 0)	43% (56, 16,952)
	Having prosocial peers	70% (10, 2145)	0% (0, 0)	0% (0, 0)	50% (2, 927)	0% (0, 0)	0% (0, 0)	0% (0, 0)	67% (12, 1993)
	Love and support from a caregiver	41% (22, 6207)	74% (46, 26,440)	89% (9, 15,234)	65% (20, 26,148)	82% (11, 19,724)	67% (3, 454)	50% (4, 1930)	67% (123, 20,097)
	Quality relationship with caregiver	46% (46, 3600)	71% (14, 8771)	67% (9, 3931)	79% (19, 12,805)	100% (4, 4416)	0% (0, 0)	100% (1, 340)	62% (104, 6379)
	Social Support	11% (9, 538)	50% (6, 11,814)	60% (5, 1909)	50% (6, 3455)	100% (1, 141)	0% (0, 0)	0% (0, 0)	47% (34, 3653)
	Support from a teacher	67% (9, 18,441)	17% (6, 11,836)	0% (0, 0)	33% (3, 82,135)	67% (3, 9241)	0% (0, 0)	0% (0, 0)	48% (21, 22,310)
	Support from non-caregiver adult (excluding teachers)	64% (11, 1854)	67% (15, 17,743)	40% (5, 1520)	17% (6, 396)	0% (2, 0)	0% (0, 0)	50% (2, 12,270)	53% (45, 9094)

SOURCE: RAND analysis of study data. NOTE: Table shows proportion of beneficial results found in research. Cells highlighted in grey are areas with high level of research activity (see Table 1), indicating more robust support for the figures displayed within. A “high” level of research activity designation was given to investigated PCE-outcome pairs with 10 or more studies, including at least 3 cohort and 3 cross-sectional studies (to prioritize areas with more robust research designs), and with average sample size of greater than 1000.

**Table 3:** Percent of tests showing a beneficial association between an environment-focused positive childhood experience and a health outcome.

		Substance Misuse	Suicidal Behaviors	Violence Perpetration	Depression	Other Mental Health	Cardiovascular Disease	Other Physical Health	Any Outcome
		% of associations where beneficial relationship detected (Total # of tests, mean sample size studies where beneficial association was detected)							
Environment	Access to green spaces and playgrounds to play	0% (0, 0)	0% (0, 0)	0% (0, 0)	100% (1, 762)	0% (0, 0)	0% (0, 0)	0% (1, 0)	67% (3, 762)
	Having access to a protective school environment	81% (36, 10,678)	63% (38, 9277)	82% (11, 11,392)	60% (10, 4231)	100% (6, 9737)	0% (0, 0)	0% (0, 0)	74% (107, 9744)
	Living in a safe neighborhood or community	40% (10, 3530)	0% (2, 0)	0% (2, 0)	0% (2, 0)	100% (1, 6483)	0% (0, 0)	0% (0, 0)	29% (17, 4121)
	Parental monitoring and related family environments	63% (51, 2311)	100% (12, 11,076)	75% (13, 2159)	100% (4, 516)	0% (0, 0)	0% (0, 0)	0% (0, 0)	72% (80, 4068)
	Regular physical activity	67% (9, 16,343)	36% (14, 14,765)	0% (0, 0)	50% (4, 7389)	100% (1, 14,306)	0% (0, 0)	0% (0, 0)	52% (29, 13,724)

SOURCE: RAND analysis of study data. NOTE: Table shows proportion of beneficial results found in research. Cells highlighted in grey are areas with high level of research activity (see Table 1), indicating more robust support for the figures displayed within. A “high” level of research activity designation was given to investigated PCE-outcome pairs with 10 or more studies, including at least 3 cohort and 3 cross-sectional studies (to prioritize areas with more robust research designs), and with average sample size of greater than 1000.

**Table 4:** Percent of tests showing a beneficial association between a social engagement-focused positive childhood experience and a health outcome.

		Substance Misuse	Suicidal Behaviors	Violence Perpetration	Depression	Other Mental Health	Cardiovascular Disease	Other Physical Health	Any Outcome
		% of associations where beneficial relationship detected (Total # of tests, mean sample size studies where beneficial association was detected)							
Social Engagement	Community connectedness	100% (2, 26,536)	100% (8, 40,146)	100% (1, 46,588)	67% (3, 42,152)	100% (2, 4722)	0% (0, 0)	0% (0, 0)	94% (16, 34,305)
	For American Indian or Alaska Native communities, native culture engagement	100% (1, 123)	0% (0, 0)	0% (0, 0)	0% (0, 0)	0% (0, 0)	0% (0, 0)	0% (0, 0)	100% (1, 123)
	Having opportunities with constructive social engagement and developing connectedness	100% (2, 450)	0% (0, 0)	0% (0, 0)	0% (1, 0)	100% (1, 129)	0% (0, 0)	0% (0, 0)	75% (4, 343)
	Having opportunity for extracurricular engagement with school or with the community	38% (8, 2681)	50% (8, 29,644)	50% (2, 18,451)	80% (5, 37,116)	67% (6, 1924)	0% (0, 0)	0% (0, 0)	56% (32, 21,960)
	Having opportunity to have beliefs that give comfort	50% (26, 1466)	100% (2, 17,143)	0% (0, 0)	50% (2, 14,272)	0% (0, 0)	0% (0, 0)	0% (0, 0)	53% (30, 4226)
	Sense of community or other cultural belonging	52% (21, 557)	0% (0, 0)	40% (5, 598)	50% (2, 1466)	0% (1, 0)	0% (0, 0)	0% (0, 0)	45% (31, 628)

SOURCE: RAND analysis of study data. NOTE: Table shows proportion of beneficial results found in research. Cells highlighted in grey are areas with high level of research activity (see Table 1), indicating more robust support for the figures displayed within. A “high” level of research activity designation was given to investigated PCE-outcome pairs with 10 or more studies, including at least 3 cohort and 3 cross-sectional studies (to prioritize areas with more robust research designs), and with average sample size of greater than 1000.

## Data Availability

The raw data supporting the conclusions of this article will be made available by the authors on request.

## Supplementary Materials

The following supporting information can be downloaded at: https://www.mdpi.com/article/10.3390/ijerph22010059/s1, File S1. PRISMA Checklist for scoping reviews, File S2. Inclusion/Exclusion Criteria, File S3. Search strategy, File S4. Included references, File S5. Summary table of studies reviewed, File S6. Percent of tests showing a beneficial association between a positive childhood experience and a health outcome.

## References

[1] Felitti V.J., Anda R.F., Nordenberg D., Williamson D.F., Spitz A.M., Edwards V., Koss M.P., Marks J.S. (1998). Relationship of childhood abuse and household dysfunction to many of the leading causes of death in adults. The Adverse Childhood Experiences (ACE) Study. Am. J. Prev. Med..

[2] Merrick M.T., Ford D.C., Ports K.A., Guinn A.S., Chen J., Klevens J., Metzler M., Jones C.M., Simon T.R., Daniel V.M. (2019). Vital Signs: Estimated Proportion of Adult Health Problems Attributable to Adverse Childhood Experiences and Implications for Prevention—25 States, 2015–2017. MMWR Morb. Mortal. Wkly. Rep..

[3] Hughes K., Bellis M.A., Hardcastle K.A., Sethi D., Butchart A., Mikton C., Jones L., Dunne M.P. (2017). The effect of multiple adverse childhood experiences on health: A systematic review and meta-analysis. Lancet Public Health.

[4] Ports K.A., Holman D.M., Guinn A.S., Pampati S., Dyer K.E., Merrick M.T., Lunsford N.B., Metzler M. (2019). Adverse Childhood Experiences and the Presence of Cancer Risk Factors in Adulthood: A Scoping Review of the Literature From 2005 to 2015. J Pediatr. Nurs..

[5] Bethell C., Jones J., Gombojav N., Linkenbach J., Sege R. (2019). Positive Childhood Experiences and Adult Mental and Relational Health in a Statewide Sample: Associations Across Adverse Childhood Experiences Levels. JAMA Pediatr..

[6] Crouch E., Radcliff E., Merrell M.A., Hung P., Bennett K.J. (2021). Positive Childhood Experiences Promote School Success. Matern. Child Health J..

[7] Crandall A., Miller J.R., Cheung A., Novilla L.K., Glade R., Novilla M.L.B., Magnusson B.M., Leavitt B.L., Barnes M.D., Hanson C.L. (2019). ACEs and counter-ACEs: How positive and negative childhood experiences influence adult health. Child Abuse Negl..

[8] Daines C.L., Hansen D., Novilla M.L.B., Crandall A. (2021). Effects of positive and negative childhood experiences on adult family health. BMC Public Health.

[9] Guo S., O’Connor M., Mensah F., Olsson C.A., Goldfeld S., Lacey R.E., Slopen N., Thurber K.A., Priest N. (2022). Measuring Positive Childhood Experiences: Testing the Structural and Predictive Validity of the Health Outcomes From Positive Experiences (HOPE) Framework. Acad. Pediatr..

[10] Graupensperger S., Kilmer J.R., Olson D.C.D., Linkenbach J.W. (2023). Associations Between Positive Childhood Experiences and Adult Smoking and Alcohol Use Behaviors in a Large Statewide Sample. J. Community Health.

[11] Wang D., Jiang Q., Yang Z., Choi J.K. (2021). The longitudinal influences of adverse childhood experiences and positive childhood experiences at family, school, and neighborhood on adolescent depression and anxiety. J. Affect. Disord..

[12] HOPE—Healthy Outcomes from Positive Experiences. https://positiveexperience.org/.

[13] Eisenberg M.E., Gower A.L., McMorris B.J., Rider G.N., Shea G., Coleman E. (2017). Risk and Protective Factors in the Lives of Transgender/Gender Nonconforming Adolescents. J. Adolesc. Health.

[14] Harper Browne C., Shapiro C., Harper Browne C. (2016). The Strengthening Families Approach and Protective Factors Framework™: A Pathway to Healthy Development and Well-Being. Innovative Approaches to Supporting Families of Young Children.

[15] Merrick J.S., Narayan A.J., DePasquale C.E., Masten A.S. (2019). Benevolent Childhood Experiences (BCEs) in homeless parents: A validation and replication study. J. Fam. Psychol..

[16] Bunting L., McCartan C., Davidson G., Grant A., Mulholland C., Schubotz D., Hamill R., McBride O., Murphy J., Nolan E. (2023). Theinfluence of adverse and positive childhood experiences on young people’s mental health and experiences of self-harm and suicidal ideation. Child Abuse Negl..

[17] Huang C.X., Halfon N., Sastry N., Chung P.J., Schickedanz A. (2023). Positive Childhood Experiences and Adult Health Outcomes. Pediatrics.

[18] Slopen N., Chen Y., Guida J.L., Albert M.A., Williams D.R. (2017). Positive childhood experiences and ideal cardiovascular health in midlife: Associations and mediators. Prev. Med..

[19] Sege R., Swedo E.A., Burstein D., Aslam M.V., Jones J., Bethell C., Niolon P.H. (2024). Prevalence of Positive Childhood Experiences Among Adults—Behavioral Risk Factor Surveillance System, Four States, 2015–2021. MMWR Morb. Mortal. Wkly. Rep..

[20] Karatekin C., Mason S.M., Riegelman A., Bakker C., Hunt S., Gresham B., Corcoran F., Barnes A. (2022). Adverse childhood experiences: A scoping review of measures and methods. Child. Youth Serv. Rev..

[21] Tzouvara V., Kupdere P., Wilson K., Matthews L., Simpson A., Foye U. (2023). Adverse childhood experiences, mental health, and social functioning: A scoping review of the literature. Child Abuse Negl..

[22] Dowling B.A., Grigsby T.J., Ziomek G.J., Schnarrs P.W. (2023). Substance Use Outcomes For Sexual and Gender Minority Adults With a History of Adverse Childhood Experiences: A Scoping Review. Drug Alcohol. Depend. Rep..

[23] Hadwen B., Pila E., Thornton J. (2022). The Associations Between Adverse Childhood Experiences, Physical and Mental Health, and Physical Activity: A Scoping Review. J. Phys. Act. Health.

[24] Navarro R., Larranaga E., Yubero S., Villora B. (2022). Associations between Adverse Childhood Experiences within the Family Context and In-Person and Online Dating Violence in Adulthood: A Scoping Review. Behav. Sci..

[25] Wang X., Jiang L., Barry L., Zhang X., Vasilenko S.A., Heath R.D. (2024). A Scoping Review on Adverse Childhood Experiences Studies Using Latent Class Analysis: Strengths and Challenges. Trauma Violence Abuse.

[26] Sege R.D., Harper Browne C. (2017). Responding to ACEs With HOPE: Health Outcomes from Positive Experiences. Acad. Pediatr..

[27] Zhang L., Fang J., Zhang D., Wan Y., Gong C., Su P., Tao F., Sun Y. (2021). Poly-victimization and psychopathological symptoms in adolescence: Examining the potential buffering effect of positive childhood experiences. J. Affect. Disord..

[28] Crandall A., Broadbent E., Stanfill M., Magnusson B.M., Novilla M.L.B., Hanson C.L., Barnes M.D. (2020). The influence of adverse and advantageous childhood experiences during adolescence on young adult health. Child Abuse Negl..

[29] Arksey H., O’Malley L. (2005). Scoping studies: Towards a methodological framework. Int. J. Soc. Res. Methodol..

[30] Hero J. (2022). Uniform Definitions and Measures for Adverse Childhood Experiences (ACES) and Childhood Protective Factors. https://archive.org/details/osf-registrations-pr8zy-v1.

[31] Gervin D.W., Holland K.M., Ottley P.G., Holmes G.M., Niolon P.H., Mercy J.A. (2022). Centers for Disease Control and Prevention Investments in Adverse Childhood Experience Prevention Efforts. Am. J. Prev. Med..

[32] Centers for Disease Control and Prevention Injury Center Research Priorities. https://www.cdc.gov/injury-violence-prevention/programs/research-priorities.html?CDC_AAref_Val=https://www.cdc.gov/injury/researchpriorities/.

[33] (2022). DistillerSR.

[34] Tricco A.C., Lillie E., Zarin W., O’Brien K.K., Colquhoun H., Levac D., Moher D., Peters M.D.J., Horsley T., Weeks L. (2018). PRISMA Extension for Scoping Reviews (PRISMA-ScR): Checklist and Explanation. Ann. Intern. Med..

[35] Centers for Disease Control and Prevention Social Determinants of Health at CDC. https://www.cdc.gov/about/priorities/why-is-addressing-sdoh-important.html?CDC_AAref_Val=https://www.cdc.gov/about/sdoh/index.html.

[36] World Health Organization Social Determinants of Health. https://www.who.int/health-topics/social-determinants-of-health#tab=tab_1.

[37] Centers for Disease Control and Prevention Adverse Childhood Experiences Research Priorities for Equitable Prevention, Intervention, Identification, and Response. https://www.cdc.gov/injury/pdfs/researchpriorities/research-priorities_aces.pdf.

[38] National Institute for Mental Health Statistics. https://www.nimh.nih.gov/health/statistics.

[39] National Institute of Health RePORTER. https://reporter.nih.gov/.

[40] Song F., Hooper L., Loke Y.K. (2013). Publication bias: What is it? How do we measure it? How do we avoid it?. Open Access J. Clin. Trials.

[41] Dwan K., Gamble C., Williamson P.R., Kirkham J.J., Group R.B. (2013). Systematic review of the empirical evidence of study publication bias and outcome reporting bias—An updated review. PLoS ONE.

