# Supplementary material for: Health Associations of Positive Childhood Experiences: A Scoping Review of the Literature
Source: Int J Environ Res Public Health. 2025 Jan 3;22(1):59. doi: 10.3390/ijerph22010059 (PMC11765245; doi:10.3390/ijerph22010059)
Supplement: Supplementary file 1 [file ijerph-22-00059-s001.zip › ijerph-3302463-supplementary.pdf]

File S1. PRISMA Checklist for scoping reviews

SECTION	ITEM	PRISMA-ScR CHECKLIST ITEM	REPORTED ON PAGE #
**TITLE**			
Title	1	Identify the report as a scoping review.	1
**ABSTRACT**			
Structured summary	2	Provide a structured summary that includes (as applicable): background, objectives, eligibility criteria, sources of evidence, charting methods, results, and conclusions that relate to the review questions and objectives.	1
**INTRODUCTION**			
Rationale	3	Describe the rationale for the review in the context of what is already known. Explain why the review questions/objectives lend themselves to a scoping review approach.	1-2
Objectives	4	Provide an explicit statement of the questions and objectives being addressed with reference to their key elements (e.g., population or participants, concepts, and context) or other relevant key elements used to conceptualize the review questions and/or objectives.	2
**METHODS**			
Protocol and registration	5	Indicate whether a review protocol exists; state if and where it can be accessed (e.g., a Web address); and if available, provide registration information, including the registration number.	3
Eligibility criteria	6	Specify characteristics of the sources of evidence used as eligibility criteria (e.g., years considered, language, and publication status), and provide a rationale.	4
Information sources*	7	Describe all information sources in the search (e.g., databases with dates of coverage and contact with authors to identify additional sources), as well as the date the most recent search was executed.	4
Search	8	Present the full electronic search strategy for at least 1 database, including any limits used, such that it could be repeated.	Appendix 1
Selection of sources of evidence†	9	State the process for selecting sources of evidence (i.e., screening and eligibility) included in the scoping review.	4
Data charting process‡	10	Describe the methods of charting data from the included sources of evidence (e.g., calibrated forms or forms that have been tested by the team before their use, and whether data charting was done independently or in duplicate) and any processes for obtaining and confirming data from investigators.	4-5
Data items	11	List and define all variables for which data were sought and any assumptions and simplifications made.	3 - 4
Critical appraisal of individual sources of evidence§	12	If done, provide a rationale for conducting a critical appraisal of included sources of evidence; describe the methods used and how this information was used in any data synthesis (if appropriate).	N/A
Synthesis of results	13	Describe the methods of handling and summarizing the data that were charted.	5 - 6
**RESULTS**			
Selection of sources of evidence	14	Give numbers of sources of evidence screened, assessed for eligibility, and included in the review, with reasons for exclusions at each stage, ideally using a flow diagram.	7
Characteristics of sources of evidence	15	For each source of evidence, present characteristics for which data were charted and provide the citations.	5 - 6
Critical appraisal within sources of evidence	16	If done, present data on critical appraisal of included sources of evidence (see item 12).	N/A
Results of individual sources of evidence	17	For each included source of evidence, present the relevant data that were charted that relate to the review questions and objectives.	7 - 12

SECTION	ITEM	PRISMA-ScR CHECKLIST ITEM	REPORTED ON PAGE #
Synthesis of results	18	Summarize and/or present the charting results as they relate to the review questions and objectives.	7 - 12
**DISCUSSION**			
Summary of evidence	19	Summarize the main results (including an overview of concepts, themes, and types of evidence available), link to the review questions and objectives, and consider the relevance to key groups.	12-13
Limitations	20	Discuss the limitations of the scoping review process.	13
Conclusions	21	Provide a general interpretation of the results with respect to the review questions and objectives, as well as potential implications and/or next steps.	13-14
**FUNDING**			
Funding	22	Describe sources of funding for the included sources of evidence, as well as sources of funding for the scoping review. Describe the role of the funders of the scoping review.	14

File S2. Inclusion/Exclusion Criteria

	**Inclusion Criteria**	**Exclusion Criteria**
Populations	Measured outcomes of populations ages ≥1 of exposures occurring before age 18. Exposure occurring at or after age 1	Samples primarily composed of individuals where outcomes were measured before 1 year of age. Samples where exposure was after age 17. Exposure occurring before age 1
Exposures	Studies whose purpose is to assess exposure to individual or combined PCEs	Studies evaluating the beneficial impacts of interventions that are in response to trauma (e.g., child protective services, crisis centers, medical care in response to an injury)
Comparators	Studies with at least one comparison group	Studies with no comparison group
Outcomes	Downstream physical health, mental health, and other high priority behavioral health outcomes (SUD, suicide, and interpersonal violence). Risk behaviors closely associated with high priority behavioral health areas will be included (e.g., fighting, suicidal ideation).	Risk behaviors not closely associated with high priority behavioral health outcomes; educational attainment; involvement with the justice system; employment; relationship stability, unsafe sex.<br>Studies where the outcome is the prevalence or incidence of ACEs, PCEs or other outcomes of interest. (Note: prevalence and incidence data may still be abstracted from included articles)
Time Frames	All timeframes for length of follow-up	None
Settings	U.S. only	Non-U.S. studies
Study Design	Meta-analyses, systematic reviews, and prospective and retrospective cohort (observational) studies with at least two exposure groups, longitudinal and cross-sectional studies. RCTs[b] will be included.	Case studies, qualitative studies, conceptual or theoretical studies, editorials, commentaries, expert opinions, non-systematic literature reviews, article summaries, letters to editors, books, theses, and conference abstracts.

PCE=positive childhood experience, SUD=substance use disorder, U.S.=United States, RCTs=randomized controlled trials.

File S3. Search strategy

PUBMED	
Date conducted	6 April 2022
# of results	4,273
Final combined	((((("Protective Factors"[Mesh] OR "Resilience, Psychological"[Mesh] OR "positive childhood experien*"[tiab] OR "childhood protective factor*"[tiab] OR ("protective factor*"[tiab] AND ("physical activity"[tiab] OR"relig*"[tiab]OR "substance use"[tiab] OR "suicid*"[tiab] OR "friend*"[tiab] OR "sports"[tiab] OR "hobby"[tiab] OR "exercise"[tiab] OR "native language"[tiab] OR "culture"[tiab] OR "attachment"[tiab])) OR "PCE"[tiab] OR "CPF"[tiab] OR "positive experien*"[tiab] OR "mitigating factor*"[tiab] OR "buffer*"[tiab] OR "resilien*"[tiab] OR "resilience factor*"[tiab] OR "flourish*"[tiab] OR "emotional stability"[tiab] OR "self esteem"[tiab] OR "self concept"[tiab] OR "emotional regulation"[tiab] OR "nurturing relation*"[tiab] OR "stable relation*"[tiab] OR "interpersonal connection*"[tiab] OR "family warmth"[tiab] OR "familial warmth"[tiab] OR "familial support"[tiab] OR "family support"[tiab] OR "parent warmth"[tiab] OR "parental warmth"[tiab] OR "emotional support"[tiab] OR "emotional growth"[tiab] OR "instrumental support"[tiab] OR "secure attachment"[tiab] OR "family strength"[tiab] OR "supportive famil*"[tiab] OR "parent* strength*"[tiab] OR "familial asset*"[tiab] OR "family asset*"[tiab] OR "developmental asset*"[tiab] OR "secure relation*"[tiab] OR "feeling safe"[tiab] OR "close relation*"[tiab] OR "social support"[tiab] OR " best friend"[tiab] OR "prosocial peer"[tiab] OR "cultural belonging"[tiab] OR "sense of belonging"[tiab] OR "safe environment"[tiab] OR "equitable environment"[tiab] OR "living environment"[tiab] OR "residential stability"[tiab] OR "stable environment"[tiab] OR "safe home"[tiab] OR "safe school"[tiab] OR "food availability"[tiab] OR "access to food"[tiab] OR "safe hous*"[tiab] OR "safe neighborhood"[tiab] OR "access to healthcare"[tiab] OR "healthcare access"[tiab] OR "behavioral health access"[tiab] OR "access to education"[tiab] OR "educational access"[tiab] OR "education access" OR "parent supervision"[tiab] OR "parental supervision"[tiab] OR "adult supervision"[tiab] OR "caregiver supervision"[tiab] OR "green space"[tiab] OR "greenspace"[tiab] OR "natural space"[tiab] OR "safe play"[tiab] OR "social engagement"[tiab] OR "civic engagement"[tiab] OR "school engagement"[tiab] OR "community engagement"[tiab] OR "cultural engagement"[tiab] OR "academic support"[tiab] OR "community traditions"[tiab] OR "compensatory experien*"[tiab] OR "protective experien*"[tiab] OR "positive youth development"[tiab] OR "youth asset*"[tiab] OR "internal asset*"[tiab] OR "caring adult*"[tiab] OR "parental support"[tiab] OR "connectedness"[tiab] OR "family connect*"[tiab] OR "family cohesion"[tiab] OR "parent* connect*"[tiab] OR "familism"[tiab] OR "friendship ties"[tiab] OR "interpersonal relationship*"[tiab] OR "social condition*"[tiab] OR "supportive community"[tiab] OR "parental monitoring"[tiab] OR "mentor*"[tiab] OR "promotive factor*"[tiab] OR "positive relation*"[tiab] OR "safe stable nurturing relationship"[tiab] OR "SSNR"[tiab] OR "benevolent childhood experien*"[tiab] OR "BCE"[tiab] OR "protective and compensatory experien*"[tiab] OR "PACEs"[tiab]) AND ("Mental Health"[Mesh] OR "Adolescent Health"[Mesh] OR "Child Health"[Mesh] OR "Family Health"[Mesh] OR "Social Determinants of Health"[Mesh] OR "physical health"[tiab] OR "health outcomes"[tiab] OR "health indicators"[tiab] OR "substance use disorder"[tiab] OR "suicid*"[tiab] OR "interpersonal violence"[tiab] OR "mental health"[tiab] OR "emotional health"[tiab] OR "psychological health"[tiab] OR "violence victimization"[tiab] OR "violence perpetration"[tiab] OR "self harm"[tiab] OR "self directed violence"[tiab] OR “mental illness”[tiab] OR “depression”[tiab] OR "substance abuse"[tiab] OR "substance use"[tiab] OR "overdose"[tiab] OR "intimate partner violence"[tiab] OR "domestic violence"[tiab] OR "domestic abuse"[tiab] OR “anxiet*”[tiab] OR “substance misuse”[tiab] OR “drug poisoning”[tiab] OR “alcohol misuse”[tiab] OR “alcohol abuse”[tiab] OR “behavioral health”[tiab])) AND ("Child"[Mesh] OR "Adolescent"[Mesh] OR "child*"[tiab] OR "kid"[tiab] OR "kids"[tiab] OR "girl*"[tiab] OR "boy"[tiab] OR "boys"[tiab] OR "teen*"[tiab] OR "youth*"[tiab] OR "youngster*"[tiab] OR "adolesc*"[tiab] OR "preschool"[tiab] OR "pre-school"[tiab] OR "kindergarten*"[tiab] OR "elementary school*"[tiab] OR "junior high"[tiab] OR "junior high school*"[tiab] OR "middle school*"[tiab] OR "high school*"[tiab] OR "juvenile"[tiab] OR "minor*"[tiab])) AND ("United States"[Mesh] OR "United States" OR "States" OR "US" OR "U.S." OR "USA" OR "U.S.A." OR "America*" OR "Appalachia*" OR "Appalachian region" OR "Georgia" OR "Kentucky" OR "North Carolina" OR "Ohio" OR "South Carolina" OR "Tennessee" OR "Virginia" OR "West Virginia" OR "Great Lakes" OR "Great Lakes Region" OR "Illinois" OR "Indiana" OR "Michigan" OR "Minnesota" OR "New York" OR "Ohio" OR "Pennsylvania" OR "Wisconsin" OR "Delaware" OR "District of Columbia" OR "Washington DC" OR "Washington D.C." OR "Maryland" OR "New Jersey" OR "Midwest United States" OR "Midwestern United States" OR "Midwest States" OR "Midwestern States" OR "Iowa" OR "Kansas" OR "Missouri" OR "Nebraska" OR "Oklahoma" OR "South Dakota" OR "Wisconsin" OR "North Dakota" OR "New England" OR "New England Region" OR "Connecticut" OR "Maine" OR "Massachusetts" OR "New Hampshire" OR "Rhode Island" OR "Vermont" OR "Northwest United States" OR "Northwestern United States" OR "Northwest States" OR "Northwestern States" OR "Idaho" OR "Montana" OR "Oregon" OR "Washington" OR "Wyoming" OR "Pacific States" OR "Alaska" OR "California" OR "Hawaii" OR "Hawai’i" OR "Southeast United States" OR "Southeastern United States" OR "Southeast States" OR "Southeastern States" OR "Alabama" OR "Arkansas" OR "Florida" OR "Georgia" OR "Louisiana" OR "Mississippi" OR "Southwest United States" OR "Southwestern United States" OR "Southwest States" OR "Southwestern States" OR "Arizona" OR "Colorado" OR "Nevada" OR "New Mexico" OR "Texas" OR "Utah" OR "Chicago" OR "New York City" OR "Baltimore" OR "Philadelphia" OR "Boston" OR "Los Angeles" OR "San Francisco" OR "New Orleans")) AND ("Cohort Studies"[Mesh] OR "Observational Study" [Publication Type] OR "Systematic Review" [Publication Type] OR "Systematic Reviews as Topic"[Mesh] OR "Meta-Analysis" [Publication Type] OR "Meta-Analysis as Topic"[Mesh] OR "Cross-Sectional Studies"[Mesh] OR "Randomized Controlled Trials as Topic"[Mesh] OR "Randomized Controlled Trial" [Publication Type]

	OR "meta analys*" OR "meta analyz*" OR "meta analyt*" OR "systematic review*" OR "systematic" OR "cross sectional stud*"[tiab] OR "cross sectional"[tiab] OR "survey"[tiab] OR "cohort stud*"[tiab] OR "observational stud*"[tiab] OR "retrospective cohort"[tiab] OR "retrospective"[tiab] OR "retrospective cohort stud*"[tiab] OR "prospective cohort"[tiab] OR "prospective"[tiab] OR "prospective cohort stud*"[tiab] OR "cohort"[tiab] OR "randomized controlled trial"[tiab] OR "randomized"[tiab] OR "RCT"[tiab] OR "randomized control"[tiab] OR "randomized trial"[tiab] OR "controlled"[tiab] OR "quasi-randomized"[tiab] OR "randomized stud*"[tiab])) AND (2014:2022[pdat])
SOCIOLOGICAL ABSTRACTS	
Date conducted	6 April 2022
# of results	428
Final combined	(MAINSUBJECT.EXACT("Prevention") OR MAINSUBJECT.EXACT("Resilience") OR TI("positive childhood experience*" OR "childhood protective factor*" OR ("protective factor*" AND ("physical activity" OR "relig*" OR "substance use" OR "suicid*" OR “friend*” OR “sports” OR “hobby” OR “exercise” OR “native language” OR “culture” OR “attachment”)) OR "PCE" OR "CPF" OR "positive experiences*" OR "mitigating factor*" OR "buffer" OR "resilient*" OR "resilience factor" OR "flourish" OR "emotional stability" OR "self esteem" OR "self concept" OR "emotional regulation" OR "nurturing relation*" OR "stable relation*" OR "interpersonal connection*" OR "family warmth" OR "familial warmth" OR "familial support" OR "family support" OR "parent warmth" OR "parental warmth" OR "emotional support" OR "emotional growth" OR "instrumental support" OR "secure attachment" OR "family strength" OR "supportive famil*" OR "parent* strength*" OR "familial asset*" OR "family asset*" OR "developmental asset*" OR "secure relation*" OR "feeling safe" OR "close relation*" OR "social support" OR "best friend" OR "prosocial peer" OR "cultural belonging" OR "sense of belonging" OR "safe environment" OR "equitable environment" OR "living environment" OR "residential stability" OR "stable environment" OR "safe home" OR "safe school" OR "food availability" OR "access to food" OR "safe hous*" OR "safe neighborhood" OR "access to healthcare" OR "healthcare access" OR "behavioral health access" OR "access to education" OR "educational access" OR "education access" OR "parent supervision" OR "parental supervision" OR "adult supervision" OR "caregiver supervision" OR "green space" OR "greenspace" OR "natural space" OR "safe play" OR "social engagement" OR "civic engagement" OR "school engagement" OR "community engagement" OR "cultural engagement" OR "academic support" OR "community traditions" OR "compensatory experien*" OR "protective experien*" OR "positive youth development" OR "youth asset*" OR "internal asset*" OR "caring adult*" OR "parental support" OR "connectedness" OR "family connect*" OR "family cohesion" OR "parent* connect*" OR "familism" OR "friendship ties" OR "interpersonal relationship*" OR "social condition*" OR "supportive community" OR "parental monitoring" OR “mentor*” OR “promotive factor*” OR “positive relation*” OR “safe stable nurturing relationship” OR “SSNR” OR “benevolent childhood experien*” OR “BCE” OR “protective and compensatory experien*” OR “PACEs”) OR AB("positive childhood experience*" OR "childhood protective factor*" OR ("protective factor*" AND ("physical activity" OR "relig*" OR "substance use" OR "suicid*" OR “friend*” OR “sports” OR “hobby” OR “exercise” OR “native language” OR “culture” OR “attachment”)) OR "PCE" OR "CPF" OR "positive experiences*" OR "mitigating factor*" OR "buffer" OR "resilient*" OR "resilience factor" OR "flourish" OR "emotional stability" OR "self esteem" OR "self concept" OR "emotional regulation" OR "nurturing relation*" OR "stable relation*" OR "interpersonal connection*" OR "family warmth" OR "familial warmth" OR "familial support" OR "family support" OR "parent warmth" OR "parental warmth" OR "emotional support" OR "emotional growth" OR "instrumental support" OR "secure attachment" OR "family strength" OR "supportive famil*" OR "parent* strength*" OR "familial asset*" OR "family asset*" OR "developmental asset*" OR "secure relation*" OR "feeling safe" OR "close relation*" OR "social support" OR "best friend" OR "prosocial peer" OR "cultural belonging" OR "sense of belonging" OR "safe environment" OR "equitable environment" OR "living environment" OR "residential stability" OR "stable environment" OR "safe home" OR "safe school" OR "food availability" OR "access to food" OR "safe hous*" OR "safe neighborhood" OR "access to healthcare" OR "healthcare access" OR "behavioral health access" OR "access to education" OR "educational access" OR "education access" OR "parent supervision" OR "parental supervision" OR "adult supervision" OR "caregiver supervision" OR "green space" OR "greenspace" OR "natural space" OR "safe play" OR "social engagement" OR "civic engagement" OR "school engagement" OR "community engagement" OR "cultural engagement" OR "academic support" OR "community traditions" OR "compensatory experien*" OR "protective experien*" OR "positive youth development" OR "youth asset*" OR "internal asset*" OR "caring adult*" OR "parental support" OR "connectedness" OR "family connect*" OR "family cohesion" OR "parent* connect*" OR "familism" OR "friendship ties" OR "interpersonal relationship*" OR "social condition*" OR "supportive community" OR "parental monitoring" OR “mentor*” OR “promotive factor*” OR “positive relation*” OR “safe stable nurturing relationship” OR “SSNR” OR “benevolent childhood experien*” OR “BCE” OR “protective and compensatory experien*” OR “PACEs”)) AND (TI(“child health” OR “adolescent health” OR “social determinants of health” OR “family health” OR "physical health" OR "mental health" OR "emotional health" OR "psychological health" OR "violence victimization" OR "violence perpetration" OR "self harm" OR “health outcomes” OR “health indicators” OR “substance use disorder” OR “suicid*” OR “interpersonal violence” OR “self directed violence” OR ‘mental illness’ OR ‘depression’ OR ‘substance abuse’ OR ‘substance use’ OR ‘overdose’ OR ‘intimate partner violence’ OR ‘domestic violence’ OR ‘domestic abuse’ OR ‘anxiet*’ OR ‘substance misuse’ OR ‘drug poisoning’ OR ‘alcohol misuse’ OR ‘alcohol abuse’ OR ‘behavioral health’) OR AB(“child health” OR “adolescent health” OR “social determinants of health” OR “family health” OR "physical health" OR "mental health" OR "emotional health" OR "psychological health" OR "violence victimization" OR "violence perpetration" OR "self harm" OR “health outcomes” OR “health indicators” OR “substance use disorder” OR “suicid*” OR “interpersonal violence” OR “self directed violence” OR ‘mental illness’ OR

	‘depression’ OR ‘substance abuse’ OR ‘substance use’ OR ‘overdose’ OR ‘intimate partner violence’ OR ‘domestic violence’ OR ‘domestic abuse’ OR ‘anxiet*’ OR ‘substance misuse’ OR ‘drug poisoning’ OR ‘alcohol misuse’ OR ‘alcohol abuse’ OR ‘behavioral health’)) AND (MAINSUBJECT.EXACT.EXPLODE("Children") OR MAINSUBJECT.EXACT.EXPLODE("Adolescents") OR TI("child*" OR "kid" OR "kids" OR "girl*" OR "boy*" OR "teen*" OR "youth*" OR "youngster" OR "adolescent*" OR "preschool" OR "pre-school" OR "kindergarten*" OR "elementary school*" OR "junior high" OR "junior high school*" OR "middle school*" OR "high school*" OR "juvenile" OR "minor*") OR AB("child*" OR "kid" OR "kids" OR "girl*" OR "boy*" OR "teen*" OR "youth*" OR "youngster" OR "adolescent*" OR "preschool" OR "pre-school" OR "kindergarten*" OR "elementary school*" OR "junior high" OR "junior high school*" OR "middle school*" OR "high school*" OR "juvenile" OR "minor*")) AND (TI("United States" OR "States" OR "US" OR "U.S." OR "USA" OR "U.S.A." OR "America*" OR "Appalachia*" OR "Appalachian region" OR "Georgia" OR "Kentucky" OR "North Carolina" OR "Ohio" OR "South Carolina" OR "Tennessee" OR "Virginia" OR "West Virginia" OR "Great Lakes" OR "Great Lakes Region" OR "Illinois" OR "Indiana" OR "Michigan" OR "Minnesota" OR "New York" OR "Ohio" OR "Pennsylvania" OR "Wisconsin" OR "Delaware" OR "District of Columbia" OR "Washington DC" OR "Washington D.C." OR "Maryland" OR "New Jersey" OR "Midwest United States" OR "Midwestern United States" OR "Midwest States" OR "Midwestern States" OR "Iowa" OR "Kansas" OR "Missouri" OR "Nebraska" OR "Oklahoma" OR "South Dakota" OR "Wisconsin" OR "North Dakota" OR "New England" OR "New England Region" OR "Connecticut" OR "Maine" OR "Massachusetts" OR "New Hampshire" OR "Rhode Island" OR "Vermont" OR "Northwest United States" OR "Northwestern United States" OR "Northwest States" OR "Northwestern States" OR "Idaho" OR "Montana" OR "Oregon" OR "Washington" OR "Wyoming" OR "Pacific States" OR "Alaska" OR "California" OR "Hawaii" OR "Hawai’i" OR "Southeast United States" OR "Southeastern United States" OR "Southeast States" OR "Southeastern States" OR "Alabama" OR "Arkansas" OR "Florida" OR "Georgia" OR "Louisiana" OR "Mississippi" OR "Southwest United States" OR "Southwestern United States" OR "Southwest States" OR "Southwestern States" OR "Arizona" OR "Colorado" OR "Nevada" OR "New Mexico" OR "Texas" OR "Utah" OR "Chicago" OR "New York City" OR "Baltimore" OR "Philadelphia" OR "Boston" OR "Los Angeles" OR "San Francisco" OR "New Orleans") OR AB("United States" OR "States" OR "US" OR "U.S." OR "USA" OR "U.S.A." OR "America*" OR "Appalachia*" OR "Appalachian region" OR "Georgia" OR "Kentucky" OR "North Carolina" OR "Ohio" OR "South Carolina" OR "Tennessee" OR "Virginia" OR "West Virginia" OR "Great Lakes" OR "Great Lakes Region" OR "Illinois" OR "Indiana" OR "Michigan" OR "Minnesota" OR "New York" OR "Ohio" OR "Pennsylvania" OR "Wisconsin" OR "Delaware" OR "District of Columbia" OR "Washington DC" OR "Washington D.C." OR "Maryland" OR "New Jersey" OR "Midwest United States" OR "Midwestern United States" OR "Midwest States" OR "Midwestern States" OR "Iowa" OR "Kansas" OR "Missouri" OR "Nebraska" OR "Oklahoma" OR "South Dakota" OR "Wisconsin" OR "North Dakota" OR "New England" OR "New England Region" OR "Connecticut" OR "Maine" OR "Massachusetts" OR "New Hampshire" OR "Rhode Island" OR "Vermont" OR "Northwest United States" OR "Northwestern United States" OR "Northwest States" OR "Northwestern States" OR "Idaho" OR "Montana" OR "Oregon" OR "Washington" OR "Wyoming" OR "Pacific States" OR "Alaska" OR "California" OR "Hawaii" OR "Hawai’i" OR "Southeast United States" OR "Southeastern United States" OR "Southeast States" OR "Southeastern States" OR "Alabama" OR "Arkansas" OR "Florida" OR "Georgia" OR "Louisiana" OR "Mississippi" OR "Southwest United States" OR "Southwestern United States" OR "Southwest States" OR "Southwestern States" OR "Arizona" OR "Colorado" OR "Nevada" OR "New Mexico" OR "Texas" OR "Utah" OR "Chicago" OR "New York City" OR "Baltimore" OR "Philadelphia" OR "Boston" OR "Los Angeles" OR "San Francisco" OR "New Orleans")) AND (MAINSUBJECT.EXACT.EXPLODE("Cohort Analysis") OR MAINSUBJECT.EXACT.EXPLODE("Observation") OR MAINSUBJECT.EXACT.EXPLODE("Surveys") OR TI("meta analyses" OR "meta analysis" OR "meta analyz*" OR "meta analytic" OR "systematic reviews" OR "systematic" OR "cross sectional stud*" OR "cross sectional" OR "survey" OR "cohort studies" OR "cohort studios" OR "cohort study" OR "observational studies" OR "observational study" OR "retrospective cohort" OR "retrospective" OR "retrospective cohort stud*" OR "prospective cohort" OR "prospective" OR "prospective cohort stud*" OR "cohort" OR "randomized controlled trial" OR "randomized" OR "RCT" OR "randomized control" OR "randomized trial" OR "controlled" OR "quasi-randomized" OR "randomized studies" OR "randomized study") OR AB("meta analyses" OR "meta analysis" OR "meta analyz*" OR "meta analytic" OR "systematic reviews" OR "systematic" OR "cross sectional stud*" OR "cross sectional" OR "survey" OR "cohort studies" OR "cohort studios" OR "cohort study" OR "observational studies" OR "observational study" OR "retrospective cohort" OR "retrospective" OR "retrospective cohort stud*" OR "prospective cohort" OR "prospective" OR "prospective cohort stud*" OR "cohort" OR "randomized controlled trial" OR "randomized" OR "RCT" OR "randomized control" OR "randomized trial" OR "controlled" OR "quasi-randomized" OR "randomized studies" OR "randomized study")) AND (PD(2014 OR 2015 OR 2016 OR 2017 OR 2018 OR 2019 OR 2020 OR 2021 OR 2022))
**PSYCINFO**	
Date conducted	6 April 2022
# of results	994
Final combined	(MAINSUBJECT.EXACT.EXPLODE("Protective Factors") OR TI("positive childhood experience*" OR "childhood protective factor*" OR ("protective factor*" AND ("physical activity" OR "relig*" OR "substance use" OR "suicid*" OR “friend*” OR “sports” OR “hobby” OR “exercise” OR “native language” OR “culture” OR “attachment”)) OR "PCE" OR "CPF" OR "positive experiences*" OR "mitigating factor*" OR "buffer" OR "resilient*" OR "resilience factor" OR "flourish" OR "emotional stability" OR "self esteem" OR "self concept" OR "emotional regulation" OR "nurturing relation*" OR "stable relation*" OR "interpersonal connection*" OR "family warmth" OR "familial warmth" OR "familial

support" OR "family support" OR "parent warmth" OR "parental warmth" OR "emotional support" OR "emotional growth" OR "instrumental support" OR "secure attachment" OR "family strength" OR "supportive famil*" OR "parent* strength*" OR "familial asset*" OR "family asset*" OR "developmental asset*" OR "secure relation*" OR "feeling safe" OR "close relation*" OR "social support" OR "best friend" OR "prosocial peer" OR "cultural belonging" OR "sense of belonging" OR "safe environment" OR "equitable environment" OR "living environment" OR "residential stability" OR "stable environment" OR "safe home" OR "safe school" OR "food availability" OR "access to food" OR "safe hous*" OR "safe neighborhood" OR "access to healthcare" OR "healthcare access" OR "behavioral health access" OR "access to education" OR "educational access" OR "education access" OR "parent supervision" OR "parental supervision" OR "adult supervision" OR "caregiver supervision" OR "green space" OR "greenspace" OR "natural space" OR "safe play" OR "social engagement" OR "civic engagement" OR "school engagement" OR "community engagement" OR "cultural engagement" OR "academic support" OR "community traditions" OR "compensatory experien*" OR "protective experien*" OR "positive youth development" OR "youth asset*" OR "internal asset*" OR "caring adult*" OR "parental support" OR "connectedness" OR "family connect*" OR "family cohesion" OR "parent* connect*" OR "familism" OR "friendship ties" OR "interpersonal relationship*" OR "social condition*" OR "supportive community" OR "parental monitoring" OR “mentor*” OR “promotive factor*” OR “positive relation*” OR “safe stable nurturing relationship” OR “SSNR” OR “benevolent childhood experien*” OR “BCE” OR “protective and compensatory experien*” OR “PACEs”) OR AB("positive childhood experience*" OR "childhood protective factor*" OR ("protective factor*" AND ("physical activity" OR "relig*" OR "substance use" OR "suicid*" OR “friend*” OR “sports” OR “hobby” OR “exercise” OR “native language” OR “culture” OR “attachment”)) OR "PCE" OR "CPF" OR "positive experiences*" OR "mitigating factor*" OR "buffer" OR "resilient*" OR "resilience factor" OR "flourish" OR "emotional stability" OR "self esteem" OR "self concept" OR "emotional regulation" OR "nurturing relation*" OR "stable relation*" OR "interpersonal connection*" OR "family warmth" OR "familial warmth" OR "familial support" OR "family support" OR "parent warmth" OR "parental warmth" OR "emotional support" OR "emotional growth" OR "instrumental support" OR "secure attachment" OR "family strength" OR "supportive famil*" OR "parent* strength*" OR "familial asset*" OR "family asset*" OR "developmental asset*" OR "secure relation*" OR "feeling safe" OR "close relation*" OR "social support" OR "best friend" OR "prosocial peer" OR "cultural belonging" OR "sense of belonging" OR "safe environment" OR "equitable environment" OR "living environment" OR "residential stability" OR "stable environment" OR "safe home" OR "safe school" OR "food availability" OR "access to food" OR "safe hous*" OR "safe neighborhood" OR "access to healthcare" OR "healthcare access" OR "behavioral health access" OR "access to education" OR "educational access" OR "education access" OR "parent supervision" OR "parental supervision" OR "adult supervision" OR "caregiver supervision" OR "green space" OR "greenspace" OR "natural space" OR "safe play" OR "social engagement" OR "civic engagement" OR "school engagement" OR "community engagement" OR "cultural engagement" OR "academic support" OR "community traditions" OR "compensatory experien*" OR "protective experien*" OR "positive youth development" OR "youth asset*" OR "internal asset*" OR "caring adult*" OR "parental support" OR "connectedness" OR "family connect*" OR "family cohesion" OR "parent* connect*" OR "familism" OR "friendship ties" OR "interpersonal relationship*" OR "social condition*" OR "supportive community" OR "parental monitoring" OR “mentor*” OR “promotive factor*” OR “positive relation*” OR “safe stable nurturing relationship” OR “SSNR” OR “benevolent childhood experien*” OR “BCE” OR “protective and compensatory experien*” OR “PACEs”)) AND (MAINSUBJECT.EXACT.EXPLODE("Mental Health") OR MAINSUBJECT.EXACT.EXPLODE("Child Health") OR MAINSUBJECT.EXACT.EXPLODE("Adolescent Health") OR TI(“family health” OR “social determinants of health” OR "physical health" OR "mental health" OR "emotional health" OR "psychological health" OR "violence victimization" OR "violence perpetration" OR "self harm" OR “health outcomes” OR “health indicators” OR “substance use disorder” OR “suicid*” OR “interpersonal violence” OR “self directed violence” OR “mental illness” OR “depression” OR “substance abuse” OR “substance use” OR “overdose” OR “intimate partner violence” OR “domestic violence” OR “domestic abuse” OR “anxiet*” OR “substance misuse” OR “drug poisoning” OR “alcohol misuse” OR “alcohol abuse” OR “behavioral health”) OR AB(“family health” OR “social determinants of health” OR "physical health" OR "mental health" OR "emotional health" OR "psychological health" OR "violence victimization" OR "violence perpetration" OR "self harm" OR “health outcomes” OR “health indicators” OR “substance use disorder” OR “suicid*” OR “interpersonal violence” OR “self directed violence” OR “mental illness” OR “depression” OR “substance abuse” OR “substance use” OR “overdose” OR “intimate partner violence” OR “domestic violence” OR “domestic abuse” OR “anxiet*” OR “substance misuse” OR “drug poisoning” OR “alcohol misuse” OR “alcohol abuse” OR “behavioral health”)) AND (TI("child*" OR "kid" OR "kids" OR "girl*" OR "boy*" OR "teen*" OR "youth*" OR "youngster" OR "adolescent*" OR "preschool" OR "pre-school" OR "kindergarten*" OR "elementary school*" OR "junior high" OR "junior high school*" OR "middle school*" OR "high school*" OR "juvenile" OR "minor*") OR AB("child*" OR "kid" OR "kids" OR "girl*" OR "boy*" OR "teen*" OR "youth*" OR "youngster" OR "adolescent*" OR "preschool" OR "pre-school" OR "kindergarten*" OR "elementary school*" OR "junior high" OR "junior high school*" OR "middle school*" OR "high school*" OR "juvenile" OR "minor*")) AND (TI("United States" OR "States" OR "US" OR "U.S." OR "USA" OR "U.S.A." OR "America*" OR "Appalachia*" OR "Appalachian region" OR "Georgia" OR "Kentucky" OR "North Carolina" OR "Ohio" OR "South Carolina" OR "Tennessee" OR "Virginia" OR "West Virginia" OR "Great Lakes" OR "Great Lakes Region" OR "Illinois" OR "Indiana" OR "Michigan" OR "Minnesota" OR "New York" OR "Ohio" OR "Pennsylvania" OR "Wisconsin" OR "Delaware" OR "District of Columbia" OR "Washington DC" OR "Washington D.C." OR "Maryland" OR "New Jersey" OR "Midwest United States" OR "Midwestern United States" OR "Midwest States" OR "Midwestern States" OR "Iowa" OR "Kansas" OR "Missouri" OR "Nebraska" OR "Oklahoma" OR "South Dakota" OR "Wisconsin" OR "North Dakota" OR "New England" OR "New England Region" OR "Connecticut" OR "Maine" OR "Massachusetts" OR "New Hampshire" OR "Rhode Island" OR "Vermont" OR "Northwest United States" OR "Northwestern United States" OR "Northwest States" OR "Northwestern States" OR "Idaho" OR "Montana" OR "Oregon" OR "Washington" OR "Wyoming" OR "Pacific States" OR "Alaska" OR "California" OR "Hawaii" OR "Hawai'i" OR "Southeast United States" OR "Southeastern United States" OR "Southeast States" OR "Southeastern States" OR "Alabama" OR "Arkansas" OR "Florida" OR "Georgia" OR "Louisiana" OR "Mississippi" OR "Southwest United States" OR "Southwestern United States" OR "Southwest States" OR "Southwestern States" OR "Arizona" OR "Colorado" OR "Nevada" OR "New Mexico" OR "Texas" OR "Utah" OR "Chicago" OR "New York City" OR "Baltimore" OR "Philadelphia" OR "Boston" OR "Los Angeles" OR "San Francisco" OR "New Orleans") OR

	AB("United States" OR "States" OR "US" OR "U.S." OR "USA" OR "U.S.A." OR "America*" OR "Appalachia*" OR "Appalachian region" OR "Georgia" OR "Kentucky" OR "North Carolina" OR "Ohio" OR "South Carolina" OR "Tennessee" OR "Virginia" OR "West Virginia" OR "Great Lakes" OR "Great Lakes Region" OR "Illinois" OR "Indiana" OR "Michigan" OR "Minnesota" OR "New York" OR "Ohio" OR "Pennsylvania" OR "Wisconsin" OR "Delaware" OR "District of Columbia" OR "Washington DC" OR "Washington D.C." OR "Maryland" OR "New Jersey" OR "Midwest United States" OR "Midwestern United States" OR "Midwest States" OR "Midwestern States" OR "Iowa" OR "Kansas" OR "Missouri" OR "Nebraska" OR "Oklahoma" OR "South Dakota" OR "Wisconsin" OR "North Dakota" OR "New England" OR "New England Region" OR "Connecticut" OR "Maine" OR "Massachusetts" OR "New Hampshire" OR "Rhode Island" OR "Vermont" OR "Northwest United States" OR "Northwestern United States" OR "Northwest States" OR "Northwestern States" OR "Idaho" OR "Montana" OR "Oregon" OR "Washington" OR "Wyoming" OR "Pacific States" OR "Alaska" OR "California" OR "Hawaii" OR "Hawai'i" OR "Southeast United States" OR "Southeastern United States" OR "Southeast States" OR "Southeastern States" OR "Alabama" OR "Arkansas" OR "Florida" OR "Georgia" OR "Louisiana" OR "Mississippi" OR "Southwest United States" OR "Southwestern United States" OR "Southwest States" OR "Southwestern States" OR "Arizona" OR "Colorado" OR "Nevada" OR "New Mexico" OR "Texas" OR "Utah" OR "Chicago" OR "New York City" OR "Baltimore" OR "Philadelphia" OR "Boston" OR "Los Angeles" OR "San Francisco" OR "New Orleans")) AND (MAINSUBJECT.EXACT.EXPLODE("Systematic Review") OR MAINSUBJECT.EXACT.EXPLODE("Cohort Analysis") OR MAINSUBJECT.EXACT.EXPLODE("Meta Analysis") OR MAINSUBJECT.EXACT.EXPLODE("Surveys") OR MAINSUBJECT.EXACT.EXPLODE("Observation Methods") OR TI("meta analyses" OR "meta analysis" OR "meta analyz*" OR "meta analytic" OR "systematic reviews" OR "systematic" OR "cross sectional stud*" OR "cross sectional" OR "survey" OR "cohort studies" OR "cohort studios" OR "cohort study" OR "observational studies" OR "observational study" OR "retrospective cohort" OR "retrospective" OR "retrospective cohort stud*" OR "prospective cohort" OR "prospective" OR "prospective cohort stud*" OR "cohort" OR "randomized controlled trial" OR "randomized" OR "RCT" OR "randomized control" OR "randomized trial" OR "controlled" OR "quasi-randomized" OR "randomized studies" OR "randomized study") OR AB("meta analyses" OR "meta analysis" OR "meta analyz*" OR "meta analytic" OR "systematic reviews" OR "systematic" OR "cross sectional stud*" OR "cross sectional" OR "survey" OR "cohort studies" OR "cohort studios" OR "cohort study" OR "observational studies" OR "observational study" OR "retrospective cohort" OR "retrospective" OR "retrospective cohort stud*" OR "prospective cohort" OR "prospective" OR "prospective cohort stud*" OR "cohort" OR "randomized controlled trial" OR "randomized" OR "RCT" OR "randomized control" OR "randomized trial" OR "controlled" OR "quasi-randomized" OR "randomized studies" OR "randomized study")) AND (PD(2014-2022))
**EMBASE**	
Date conducted	6 April 2022
# of results	6,092
Final combined	No. Query Results / Results / Date<br>#7. ('protective factor'/exp OR 'psychological resilience'/exp OR 'positive childhood experien*':ti,ab,kw OR 'childhood protective factor*':ti,ab,kw OR ('protective factor*':ti,ab,kw AND ('physical activity':ti,ab,kw OR 'relig*':ti,ab,kw OR 'substance use':ti,ab,kw OR 'suicid*':ti,ab,kw OR 'friend*':ti,ab,kw OR 'sports':ti,ab,kw OR 'hobby':ti,ab,kw OR 'exercise':ti,ab,kw OR 'native language':ti,ab,kw OR 'culture':ti,ab,kw / 6,092 / 6 Apr 2022

OR 'attachment':ti,ab,kw)) OR 'pce':ti,ab,kw OR
'cpf':ti,ab,kw OR 'positive experien*':ti,ab,kw
OR 'mitigating factor*':ti,ab,kw OR
'buffer*':ti,ab,kw OR 'resilien*':ti,ab,kw OR
'resilience factor*':ti,ab,kw OR
'flourish*':ti,ab,kw OR 'emotional
stability':ti,ab,kw OR 'self esteem':ti,ab,kw OR
'self concept':ti,ab,kw OR 'emotional
regulation':ti,ab,kw OR 'nurturing
relation*':ti,ab,kw OR 'stable
relation*':ti,ab,kw OR 'interpersonal
connection*':ti,ab,kw OR 'family warmth':ti,ab,kw
OR 'familial warmth':ti,ab,kw OR 'familial
support':ti,ab,kw OR 'family support':ti,ab,kw OR
'parent warmth':ti,ab,kw OR 'parental
warmth':ti,ab,kw OR 'emotional support':ti,ab,kw
OR 'emotional growth':ti,ab,kw OR 'instrumental
support':ti,ab,kw OR 'secure attachment':ti,ab,kw
OR 'family strength':ti,ab,kw OR 'supportive
famil*':ti,ab,kw OR 'parent* strength*':ti,ab,kw
OR 'familial asset*':ti,ab,kw OR 'family
asset*':ti,ab,kw OR 'developmental
asset*':ti,ab,kw OR 'secure relation*':ti,ab,kw
OR 'feeling safe':ti,ab,kw OR 'close
relation*':ti,ab,kw OR 'social support':ti,ab,kw
OR 'best friend':ti,ab,kw OR 'prosocial
peer':ti,ab,kw OR 'cultural belonging':ti,ab,kw
OR 'sense of belonging':ti,ab,kw OR 'safe

environment':ti,ab,kw OR 'equitable
environment':ti,ab,kw OR 'living
environment':ti,ab,kw OR 'residential
stability':ti,ab,kw OR 'stable
environment':ti,ab,kw OR 'safe home':ti,ab,kw OR
'safe school':ti,ab,kw OR 'food
availability':ti,ab,kw OR 'access to
food':ti,ab,kw OR 'safe hous*':ti,ab,kw OR 'safe
neighborhood':ti,ab,kw OR 'access to
healthcare':ti,ab,kw OR 'healthcare
access':ti,ab,kw OR 'behavioral health
access':ti,ab,kw OR 'access to
education':ti,ab,kw OR 'educational
access':ti,ab,kw OR 'education access':ti,ab,kw
OR 'parent supervision':ti,ab,kw OR 'parental
supervision':ti,ab,kw OR 'adult
supervision':ti,ab,kw OR 'caregiver
supervision':ti,ab,kw OR 'green space':ti,ab,kw
OR 'greenspace':ti,ab,kw OR 'natural
space':ti,ab,kw OR 'safe play':ti,ab,kw OR
'social engagement':ti,ab,kw OR 'civic
engagement':ti,ab,kw OR 'school
engagement':ti,ab,kw OR 'community
engagement':ti,ab,kw OR 'cultural
engagement':ti,ab,kw OR 'academic
support':ti,ab,kw OR 'community
traditions':ti,ab,kw OR 'compensatory
experien*':ti,ab,kw OR 'protective

experien*':ti,ab,kw OR 'positive youth development':ti,ab,kw OR 'youth asset*':ti,ab,kw OR 'internal asset*':ti,ab,kw OR 'caring adult*':ti,ab,kw OR 'parental support':ti,ab,kw OR 'connectedness':ti,ab,kw OR 'family connect*':ti,ab,kw OR 'family cohesion':ti,ab,kw OR 'parent* connect*':ti,ab,kw OR 'familism':ti,ab,kw OR 'friendship ties':ti,ab,kw OR 'interpersonal relationship*':ti,ab,kw OR 'social condition*':ti,ab,kw OR 'supportive community':ti,ab,kw OR 'parental monitoring':ti,ab,kw OR 'mentor*':ti,ab,kw OR 'promotive factor*':ti,ab,kw OR 'positive relation*':ti,ab,kw OR 'safe stable nurturing relationship':ti,ab,kw OR 'ssnr':ti,ab,kw OR 'benevolent childhood experien*':ti,ab,kw OR 'bce':ti,ab,kw OR 'protective and compensatory experien*':ti,ab,kw OR 'paces':ti,ab,kw) AND ('mental health'/exp OR 'adolescent health'/exp OR 'child health'/exp OR 'family health'/exp OR 'social determinants of health'/exp OR 'physical health':ti,ab,kw OR 'health outcomes':ti,ab,kw OR 'health indicators':ti,ab,kw OR 'substance use disorder':ti,ab,kw OR 'suicid*':ti,ab,kw OR 'interpersonal violence':ti,ab,kw OR 'mental health':ti,ab,kw OR 'emotional health':ti,ab,kw OR 'psychological health':ti,ab,kw OR 'violence victimization':ti,ab,kw OR 'violence

perpetration':ti,ab,kw OR 'self harm':ti,ab,kw OR 'self directed violence':ti,ab,kw OR 'mental illness':ti,ab,kw OR 'substance abuse':ti,ab,kw OR 'substance use':ti,ab,kw OR 'overdose':ti,ab,kw OR 'intimate partner violence':ti,ab,kw OR 'domestic violence':ti,ab,kw OR 'domestic abuse':ti,ab,kw OR 'anxiet*':ti,ab,kw OR 'substance misuse':ti,ab,kw OR 'drug poisoning':ti,ab,kw OR 'alcohol misuse':ti,ab,kw OR 'alcohol abuse':ti,ab,kw OR 'behavioral health':ti,ab,kw) AND ('child'/exp OR 'adolescent'/exp OR 'child*' OR 'kid':ti,ab,kw OR 'kids':ti,ab,kw OR 'girl*':ti,ab,kw OR 'boy':ti,ab,kw OR 'boys':ti,ab,kw OR 'teen*':ti,ab,kw OR 'youth*':ti,ab,kw OR 'youngster*':ti,ab,kw OR 'adolesc*':ti,ab,kw OR 'preschool':ti,ab,kw OR 'pre-school':ti,ab,kw OR 'kindergarten*':ti,ab,kw OR 'elementary school*':ti,ab,kw OR 'junior high':ti,ab,kw OR 'junior high school*':ti,ab,kw OR 'middle school*':ti,ab,kw OR 'high school*':ti,ab,kw OR 'juvenile':ti,ab,kw OR 'minor*':ti,ab,kw) AND ('united states'/exp OR 'united states' OR 'states' OR 'us' OR 'u.s.' OR 'usa' OR 'u.s.a.' OR 'america*' OR 'appalachia*' OR 'appalachian region' OR 'kentucky' OR 'north carolina' OR 'south carolina' OR 'tennessee' OR 'virginia' OR 'west virginia' OR 'great lakes' OR

'great lakes region' OR 'illinois' OR 'indiana' OR 'michigan' OR 'minnesota' OR 'new york' OR 'ohio' OR 'pennsylvania' OR 'delaware' OR 'district of columbia' OR 'washington dc' OR 'washington d.c.' OR 'maryland' OR 'new jersey' OR 'midwest united states' OR 'midwestern united states' OR 'midwest states' OR 'midwestern states' OR 'iowa' OR 'kansas' OR 'missouri' OR 'nebraska' OR 'oklahoma' OR 'south dakota' OR 'wisconsin' OR 'north dakota' OR 'new england' OR 'new england region' OR 'connecticut' OR 'maine' OR 'massachusetts' OR 'new hampshire' OR 'rhode island' OR 'vermont' OR 'northwest united states' OR 'northwestern united states' OR 'northwest states' OR 'northwestern states' OR 'idaho' OR 'montana' OR 'oregon' OR 'washington' OR 'wyoming' OR 'pacific states' OR 'alaska' OR 'california' OR 'hawaii' OR 'southeast united states' OR 'southeastern united states' OR 'southeast states' OR 'southeastern states' OR 'alabama' OR 'arkansas' OR 'florida' OR 'georgia' OR 'louisiana' OR 'mississippi' OR 'southwest united states' OR 'southwestern united states' OR 'southwest states' OR 'southwestern states' OR 'arizona' OR 'colorado' OR 'nevada' OR 'new mexico' OR 'texas' OR 'utah' OR 'chicago' OR 'new york city' OR 'baltimore' OR 'philadelphia' OR 'boston' OR 'los angeles' OR 'san francisco' OR

	'new orleans') AND ('cohort analysis'/exp OR 'cohort analysis topic'/exp OR 'observational study'/exp OR 'systematic review'/exp OR 'systematic review (topic)'/exp OR 'meta analysis'/exp OR 'meta analysis (topic)'/exp OR 'cross-sectional study'/exp OR 'cross sectional study topic'/exp OR 'randomized controlled trial'/exp OR 'randomized controlled trial (topic)'/exp OR 'cross sectional stud*':ti,ab,kw OR 'cross sectional':ti,ab,kw OR 'survey':ti,ab,kw OR 'cohort stud*':ti,ab,kw OR 'observational stud*':ti,ab,kw OR 'retrospective cohort':ti,ab,kw OR 'retrospective':ti,ab,kw OR 'retrospective cohort stud*':ti,ab,kw OR 'prospective cohort':ti,ab,kw OR 'prospective':ti,ab,kw OR 'prospective cohort stud*':ti,ab,kw OR 'cohort':ti,ab,kw OR 'randomized controlled trial':ti,ab,kw OR 'randomized':ti,ab,kw OR 'rct':ti,ab,kw OR 'randomized control':ti,ab,kw OR 'randomized trial':ti,ab,kw OR 'controlled':ti,ab,kw OR 'quasi-randomized':ti,ab,kw OR 'randomized stud*':ti,ab,kw) AND (2014:py OR 2015:py OR 2016:py OR 2017:py OR 2018:py OR 2019:py OR 2020:py OR 2021:py OR 2022:py)		
#6.	2014:py OR 2015:py OR 2016:py OR 2017:py OR 2018:py OR 2019:py OR 2020:py OR 2021:py OR 2022:py	13,390,689	6 Apr 2022

#5. 'cohort analysis'/exp OR 'cohort analysis topic'/exp OR 'observational study'/exp OR 'systematic review'/exp OR 'systematic review (topic)'/exp OR 'meta analysis'/exp OR 'meta analysis (topic)'/exp OR 'cross-sectional study'/exp OR 'cross sectional study topic'/exp OR 'randomized controlled trial'/exp OR 'randomized controlled trial (topic)'/exp OR 'cross sectional stud*':ti,ab,kw OR 'cross sectional':ti,ab,kw OR 'survey':ti,ab,kw OR 'cohort stud*':ti,ab,kw OR 'observational stud*':ti,ab,kw OR 'retrospective cohort':ti,ab,kw OR 'retrospective':ti,ab,kw OR 'retrospective cohort stud*':ti,ab,kw OR 'prospective cohort':ti,ab,kw OR 'prospective':ti,ab,kw OR 'prospective cohort stud*':ti,ab,kw OR 'cohort':ti,ab,kw OR 'randomized controlled trial':ti,ab,kw OR 'randomized':ti,ab,kw OR 'rct':ti,ab,kw OR 'randomized control':ti,ab,kw OR 'randomized trial':ti,ab,kw OR 'controlled':ti,ab,kw OR 'quasi-randomized':ti,ab,kw OR 'randomized stud*':ti,ab,kw	5,937,847	6 Apr 2022
#4. 'united states'/exp OR 'united states' OR 'states' OR 'us' OR 'u.s.' OR 'usa' OR 'u.s.a.' OR 'america*' OR 'appalachia*' OR 'appalachian region' OR 'kentucky' OR 'north carolina' OR 'south carolina' OR 'tennessee' OR 'virginia' OR	21,198,437	6 Apr 2022

'west virginia' OR 'great lakes' OR 'great lakes region' OR 'illinois' OR 'indiana' OR 'michigan' OR 'minnesota' OR 'new york' OR 'ohio' OR 'pennsylvania' OR 'delaware' OR 'district of columbia' OR 'washington dc' OR 'washington d.c.' OR 'maryland' OR 'new jersey' OR 'midwest united states' OR 'midwestern united states' OR 'midwest states' OR 'midwestern states' OR 'iowa' OR 'kansas' OR 'missouri' OR 'nebraska' OR 'oklahoma' OR 'south dakota' OR 'wisconsin' OR 'north dakota' OR 'new england' OR 'new england region' OR 'connecticut' OR 'maine' OR 'massachusetts' OR 'new hampshire' OR 'rhode island' OR 'vermont' OR 'northwest united states' OR 'northwestern united states' OR 'northwest states' OR 'northwestern states' OR 'idaho' OR 'montana' OR 'oregon' OR 'washington' OR 'wyoming' OR 'pacific states' OR 'alaska' OR 'california' OR 'hawaii' OR 'southeast united states' OR 'southeastern united states' OR 'southeast states' OR 'southeastern states' OR 'alabama' OR 'arkansas' OR 'florida' OR 'georgia' OR 'louisiana' OR 'mississippi' OR 'southwest united states' OR 'southwestern united states' OR 'southwest states' OR 'southwestern states' OR 'arizona' OR 'colorado' OR 'nevada' OR 'new mexico' OR 'texas' OR 'utah' OR 'chicago' OR 'new york city' OR 'baltimore' OR 'philadelphia' OR

	'boston' OR 'los angeles' OR 'san francisco' OR 'new orleans'		
#3.	'child'/exp OR 'adolescent'/exp OR 'child*' OR 'kid':ti,ab,kw OR 'kids':ti,ab,kw OR 'girl*':ti,ab,kw OR 'boy':ti,ab,kw OR 'boys':ti,ab,kw OR 'teen*':ti,ab,kw OR 'youth*':ti,ab,kw OR 'youngster*':ti,ab,kw OR 'adolesc*':ti,ab,kw OR 'preschool':ti,ab,kw OR 'pre-school':ti,ab,kw OR 'kindergarten*':ti,ab,kw OR 'elementary school*':ti,ab,kw OR 'junior high':ti,ab,kw OR 'junior high school*':ti,ab,kw OR 'middle school*':ti,ab,kw OR 'high school*':ti,ab,kw OR 'juvenile':ti,ab,kw OR 'minor*':ti,ab,kw	5,644,719	6 Apr 2022
#2.	'mental health'/exp OR 'adolescent health'/exp OR 'child health'/exp OR 'family health'/exp OR 'social determinants of health'/exp OR 'physical health':ti,ab,kw OR 'health outcomes':ti,ab,kw OR 'health indicators':ti,ab,kw OR 'substance use disorder':ti,ab,kw OR 'suicid*':ti,ab,kw OR 'interpersonal violence':ti,ab,kw OR 'mental health':ti,ab,kw OR 'emotional health':ti,ab,kw OR 'psychological health':ti,ab,kw OR 'violence victimization':ti,ab,kw OR 'violence perpetration':ti,ab,kw OR 'self harm':ti,ab,kw OR 'self directed violence':ti,ab,kw OR 'mental illness':ti,ab,kw OR 'substance abuse':ti,ab,kw OR 'substance use':ti,ab,kw OR	999,719	6 Apr 2022

'overdose':ti,ab,kw OR 'intimate partner
violence':ti,ab,kw OR 'domestic
violence':ti,ab,kw OR 'domestic abuse':ti,ab,kw
OR 'anxiet*':ti,ab,kw OR 'substance
misuse':ti,ab,kw OR 'drug poisoning':ti,ab,kw OR
'alcohol misuse':ti,ab,kw OR 'alcohol
abuse':ti,ab,kw OR 'behavioral health':ti,ab,kw

#1. 'protective factor'/exp OR 'psychological resilience'/exp OR 'positive childhood experien*':ti,ab,kw OR 'childhood protective factor*':ti,ab,kw OR ('protective factor*':ti,ab,kw AND ('physical activity':ti,ab,kw OR 'relig*':ti,ab,kw OR 'substance use':ti,ab,kw OR 'suicid*':ti,ab,kw OR 'friend*':ti,ab,kw OR 'sports':ti,ab,kw OR 'hobby':ti,ab,kw OR 'exercise':ti,ab,kw OR 'native language':ti,ab,kw OR 'culture':ti,ab,kw OR 'attachment':ti,ab,kw)) OR 'pce':ti,ab,kw OR 'cpf':ti,ab,kw OR 'positive experien*':ti,ab,kw OR 'mitigating factor*':ti,ab,kw OR 'buffer*':ti,ab,kw OR 'resilien*':ti,ab,kw OR 'resilience factor*':ti,ab,kw OR 'flourish*':ti,ab,kw OR 'emotional stability':ti,ab,kw OR 'self esteem':ti,ab,kw OR 'self concept':ti,ab,kw OR 'emotional regulation':ti,ab,kw OR 'nurturing relation*':ti,ab,kw OR 'stable relation*':ti,ab,kw OR 'interpersonal	514,760	6 Apr 2022

connection*':ti,ab,kw OR 'family warmth':ti,ab,kw OR 'familial warmth':ti,ab,kw OR 'familial support':ti,ab,kw OR 'family support':ti,ab,kw OR 'parent warmth':ti,ab,kw OR 'parental warmth':ti,ab,kw OR 'emotional support':ti,ab,kw OR 'emotional growth':ti,ab,kw OR 'instrumental support':ti,ab,kw OR 'secure attachment':ti,ab,kw OR 'family strength':ti,ab,kw OR 'supportive famil*':ti,ab,kw OR 'parent* strength*':ti,ab,kw OR 'familial asset*':ti,ab,kw OR 'family asset*':ti,ab,kw OR 'developmental asset*':ti,ab,kw OR 'secure relation*':ti,ab,kw OR 'feeling safe':ti,ab,kw OR 'close relation*':ti,ab,kw OR 'social support':ti,ab,kw OR 'best friend':ti,ab,kw OR 'prosocial peer':ti,ab,kw OR 'cultural belonging':ti,ab,kw OR 'sense of belonging':ti,ab,kw OR 'safe environment':ti,ab,kw OR 'equitable environment':ti,ab,kw OR 'living environment':ti,ab,kw OR 'residential stability':ti,ab,kw OR 'stable environment':ti,ab,kw OR 'safe home':ti,ab,kw OR 'safe school':ti,ab,kw OR 'food availability':ti,ab,kw OR 'access to food':ti,ab,kw OR 'safe hous*':ti,ab,kw OR 'safe neighborhood':ti,ab,kw OR 'access to healthcare':ti,ab,kw OR 'healthcare access':ti,ab,kw OR 'behavioral health

access':ti,ab,kw OR 'access to education':ti,ab,kw OR 'educational access':ti,ab,kw OR 'education access':ti,ab,kw OR 'parent supervision':ti,ab,kw OR 'parental supervision':ti,ab,kw OR 'adult supervision':ti,ab,kw OR 'caregiver supervision':ti,ab,kw OR 'green space':ti,ab,kw OR 'greenspace':ti,ab,kw OR 'natural space':ti,ab,kw OR 'safe play':ti,ab,kw OR 'social engagement':ti,ab,kw OR 'civic engagement':ti,ab,kw OR 'school engagement':ti,ab,kw OR 'community engagement':ti,ab,kw OR 'cultural engagement':ti,ab,kw OR 'academic support':ti,ab,kw OR 'community traditions':ti,ab,kw OR 'compensatory experien*':ti,ab,kw OR 'protective experien*':ti,ab,kw OR 'positive youth development':ti,ab,kw OR 'youth asset*':ti,ab,kw OR 'internal asset*':ti,ab,kw OR 'caring adult*':ti,ab,kw OR 'parental support':ti,ab,kw OR 'connectedness':ti,ab,kw OR 'family connect*':ti,ab,kw OR 'family cohesion':ti,ab,kw OR 'parent* connect*':ti,ab,kw OR 'familism':ti,ab,kw OR 'friendship ties':ti,ab,kw OR 'interpersonal relationship*':ti,ab,kw OR 'social condition*':ti,ab,kw OR 'supportive community':ti,ab,kw OR 'parental

	monitoring':ti,ab,kw OR 'mentor*':ti,ab,kw OR 'promotive factor*':ti,ab,kw OR 'positive relation*':ti,ab,kw OR 'safe stable nurturing relationship':ti,ab,kw OR 'ssnr':ti,ab,kw OR 'benevolent childhood experien*':ti,ab,kw OR 'bce':ti,ab,kw OR 'protective and compensatory experien*':ti,ab,kw OR 'paces':ti,ab,kw
WEB OF SCIENCE	
Date conducted	6 April 2022
# of results	1,241
Final combined	(TI=(("positive childhood experience*" OR "childhood protective factor*" OR ("protective factor*" AND ("physical activity" OR "relig*" OR "substance use" OR "suicid*" OR “friend*” OR “sports” OR “hobby” OR “exercise” OR “native language” OR “culture” OR “attachment”)) OR "PCE" OR "CPF" OR "positive experiences*" OR "mitigating factor*" OR "buffer" OR "resilient*" OR "resilience factor" OR "flourish" OR "emotional stability" OR "self esteem" OR "self concept" OR "emotional regulation" OR "nurturing relation*" OR "stable relation*" OR "interpersonal connection*" OR "family warmth" OR "familial warmth" OR "familial support" OR "family support" OR "parent warmth" OR "parental warmth" OR "emotional support" OR "emotional growth" OR "instrumental support" OR "secure attachment" OR "family strength" OR "supportive famil*" OR "parent* strength*" OR "familial asset*" OR "family asset*" OR "developmental asset*" OR "secure relation*" OR "feeling safe" OR "close relation*" OR "social support" OR "best friend" OR "prosocial peer" OR "cultural belonging" OR "sense of belonging" OR "safe environment" OR "equitable environment" OR "living environment" OR "residential stability" OR "stable environment" OR "safe home" OR "safe school" OR "food availability" OR "access to food" OR "safe hous*" OR "safe neighborhood" OR "access to healthcare" OR "healthcare access" OR "behavioral health access" OR "access to education" OR "educational access" OR "education access" OR "parent supervision" OR "parental supervision" OR "adult supervision" OR "caregiver supervision" OR "green space" OR "greenspace" OR "natural space" OR "safe play" OR "social engagement" OR "civic engagement" OR "school engagement" OR "community engagement" OR "cultural engagement" OR "academic support" OR "community traditions" OR "compensatory experien*" OR "protective experien*" OR "positive youth development" OR "youth asset*" OR "internal asset*" OR "caring adult*" OR "parental support" OR "connectedness" OR "family connect*" OR "family cohesion" OR "parent* connect*" OR "familism" OR "friendship ties" OR "interpersonal relationship*" OR "social condition*" OR "supportive community" OR "parental monitoring" OR “mentor*” OR “promotive factor*” OR “positive relation*” OR “safe stable nurturing relationship” OR “SSNR” OR “benevolent childhood experien*” OR “BCE” OR “protective and compensatory experien*” OR “PACEs”)) OR AB=(("positive childhood experience*" OR "childhood protective factor*" OR ("protective factor*" AND ("physical activity" OR "relig*" OR "substance use" OR "suicid*" OR “friend*” OR “sports” OR “hobby” OR “exercise” OR “native language” OR “culture” OR “attachment”)) OR "PCE" OR "CPF" OR "positive experiences*" OR "mitigating factor*" OR "buffer" OR "resilient*" OR "resilience factor" OR "flourish" OR "emotional stability" OR "self esteem" OR "self concept" OR "emotional regulation" OR "nurturing relation*" OR "stable relation*" OR "interpersonal connection*" OR "family warmth" OR "familial warmth" OR "familial support" OR "family support" OR "parent warmth" OR "parental warmth" OR "emotional support" OR "emotional growth" OR "instrumental support" OR "secure attachment" OR "family strength" OR "supportive famil*" OR "parent* strength*" OR "familial asset*" OR "family asset*" OR "developmental asset*" OR "secure relation*" OR "feeling safe" OR "close relation*" OR "social support" OR "best friend" OR "prosocial peer" OR "cultural belonging" OR "sense of belonging" OR "safe environment" OR "equitable environment" OR "living environment" OR "residential stability" OR "stable environment" OR "safe home" OR "safe school" OR "food availability" OR "access to food" OR "safe hous*" OR "safe neighborhood" OR "access to healthcare" OR "healthcare access" OR "behavioral health access" OR "access to education" OR "educational access" OR "education access" OR "parent supervision" OR "parental supervision" OR "adult supervision" OR "caregiver supervision" OR "green space" OR "greenspace" OR "natural space" OR "safe play" OR "social engagement" OR "civic engagement" OR "school engagement" OR "community engagement" OR "cultural engagement" OR "academic support" OR "community traditions" OR "compensatory experien*" OR "protective experien*" OR "positive youth development" OR "youth asset*" OR "internal asset*" OR "caring adult*" OR "parental support" OR "connectedness" OR "family connect*" OR "family cohesion" OR "parent* connect*" OR "familism" OR "friendship ties" OR "interpersonal relationship*" OR "social condition*" OR "supportive community" OR "parental monitoring" OR “mentor*” OR “promotive factor*” OR “positive relation*” OR “safe stable nurturing relationship” OR “SSNR” OR “benevolent childhood experien*” OR “BCE” OR “protective and compensatory experien*” OR “PACEs”))) AND (TI=(“family health” OR “social

determinants of health” OR "physical health" OR "mental health" OR "emotional health" OR "psychological health" OR "violence victimization" OR "violence perpetration" OR "self harm" OR “health outcomes” OR “health indicators” OR “substance use disorder” OR “suicid*” OR “interpersonal violence” OR “self directed violence” OR “mental illness” OR “depression” OR “substance abuse” OR “substance use” OR “overdose” OR “intimate partner violence” OR “domestic violence” OR “domestic abuse” OR “anxiet*” OR “substance misuse” OR “drug poisoning” OR “alcohol misuse” OR “alcohol abuse” OR “behavioral health”) OR AB=(“family health” OR “social determinants of health” OR "physical health" OR "mental health" OR "emotional health" OR "psychological health" OR "violence victimization" OR "violence perpetration" OR "self harm" OR “health outcomes” OR “health indicators” OR “substance use disorder” OR “suicid*” OR “interpersonal violence” OR “self directed violence” OR “mental illness” OR “depression” OR “substance abuse” OR “substance use” OR “overdose” OR “intimate partner violence” OR “domestic violence” OR “domestic abuse” OR “anxiet*” OR “substance misuse” OR “drug poisoning” OR “alcohol misuse” OR “alcohol abuse” OR “behavioral health”)) AND (TI=("child*" OR "kid" OR "kids" OR "girl*" OR "boy*" OR "teen*" OR "youth*" OR "youngster" OR "adolescent*" OR "preschool" OR "pre-school" OR "kindergarten*" OR "elementary school*" OR "junior high" OR "junior high school*" OR "middle school*" OR "high school*" OR "juvenile" OR "minor*") OR AB=("child*" OR "kid" OR "kids" OR "girl*" OR "boy*" OR "teen*" OR "youth*" OR "youngster" OR "adolescent*" OR "preschool" OR "pre-school" OR "kindergarten*" OR "elementary school*" OR "junior high" OR "junior high school*" OR "middle school*" OR "high school*" OR "juvenile" OR "minor*")) AND (TI=("United States" OR "States" OR "US" OR "U.S." OR "USA" OR "U.S.A." OR "America*" OR "Appalachia*" OR "Appalachian region" OR "Georgia" OR "Kentucky" OR "North Carolina" OR "Ohio" OR "South Carolina" OR "Tennessee" OR "Virginia" OR "West Virginia" OR "Great Lakes" OR "Great Lakes Region" OR "Illinois" OR "Indiana" OR "Michigan" OR "Minnesota" OR "New York" OR "Ohio" OR "Pennsylvania" OR "Wisconsin" OR "Delaware" OR "District of Columbia" OR "Washington DC" OR "Washington D.C." OR "Maryland" OR "New Jersey" OR "Midwest United States" OR "Midwestern United States" OR "Midwest States" OR "Midwestern States" OR "Iowa" OR "Kansas" OR "Missouri" OR "Nebraska" OR "Oklahoma" OR "South Dakota" OR "Wisconsin" OR "North Dakota" OR "New England" OR "New England Region" OR "Connecticut" OR "Maine" OR "Massachusetts" OR "New Hampshire" OR "Rhode Island" OR "Vermont" OR "Northwest United States" OR "Northwestern United States" OR "Northwest States" OR "Northwestern States" OR "Idaho" OR "Montana" OR "Oregon" OR "Washington" OR "Wyoming" OR "Pacific States" OR "Alaska" OR "California" OR "Hawaii" OR "Hawai’i" OR "Southeast United States" OR "Southeastern United States" OR "Southeast States" OR "Southeastern States" OR "Alabama" OR "Arkansas" OR "Florida" OR "Georgia" OR "Louisiana" OR "Mississippi" OR "Southwest United States" OR "Southwestern United States" OR "Southwest States" OR "Southwestern States" OR "Arizona" OR "Colorado" OR "Nevada" OR "New Mexico" OR "Texas" OR "Utah" OR "Chicago" OR "New York City" OR "Baltimore" OR "Philadelphia" OR "Boston" OR "Los Angeles" OR "San Francisco" OR "New Orleans") OR AB=("United States" OR "States" OR "US" OR "U.S." OR "USA" OR "U.S.A." OR "America*" OR "Appalachia*" OR "Appalachian region" OR "Georgia" OR "Kentucky" OR "North Carolina" OR "Ohio" OR "South Carolina" OR "Tennessee" OR "Virginia" OR "West Virginia" OR "Great Lakes" OR "Great Lakes Region" OR "Illinois" OR "Indiana" OR "Michigan" OR "Minnesota" OR "New York" OR "Ohio" OR "Pennsylvania" OR "Wisconsin" OR "Delaware" OR "District of Columbia" OR "Washington DC" OR "Washington D.C." OR "Maryland" OR "New Jersey" OR "Midwest United States" OR "Midwestern United States" OR "Midwest States" OR "Midwestern States" OR "Iowa" OR "Kansas" OR "Missouri" OR "Nebraska" OR "Oklahoma" OR "South Dakota" OR "Wisconsin" OR "North Dakota" OR "New England" OR "New England Region" OR "Connecticut" OR "Maine" OR "Massachusetts" OR "New Hampshire" OR "Rhode Island" OR "Vermont" OR "Northwest United States" OR "Northwestern United States" OR "Northwest States" OR "Northwestern States" OR "Idaho" OR "Montana" OR "Oregon" OR "Washington" OR "Wyoming" OR "Pacific States" OR "Alaska" OR "California" OR "Hawaii" OR "Hawai’i" OR "Southeast United States" OR "Southeastern United States" OR "Southeast States" OR "Southeastern States" OR "Alabama" OR "Arkansas" OR "Florida" OR "Georgia" OR "Louisiana" OR "Mississippi" OR "Southwest United States" OR "Southwestern United States" OR "Southwest States" OR "Southwestern States" OR "Arizona" OR "Colorado" OR "Nevada" OR "New Mexico" OR "Texas" OR "Utah" OR "Chicago" OR "New York City" OR "Baltimore" OR "Philadelphia" OR "Boston" OR "Los Angeles" OR "San Francisco" OR "New Orleans")) AND (TI=("meta analyses" OR "meta analysis" OR "meta analyz*" OR "meta analytic" OR "systematic reviews" OR "systematic" OR "cross sectional stud*" OR "cross sectional" OR "survey" OR "cohort studies" OR "cohort studios" OR "cohort study" OR "observational studies" OR "observational study" OR "retrospective cohort" OR "retrospective" OR "retrospective cohort stud*" OR "prospective cohort" OR "prospective" OR "prospective cohort stud*" OR "cohort" OR "randomized controlled trial" OR "randomized" OR "RCT" OR "randomized control" OR "randomized trial" OR "controlled" OR "quasi-randomized" OR "randomized studies" OR "randomized study") OR AB=("meta analyses" OR "meta analysis" OR "meta analyz*" OR "meta analytic" OR "systematic reviews" OR "systematic" OR "cross sectional stud*" OR "cross sectional" OR "survey" OR "cohort studies" OR "cohort studios" OR "cohort study" OR "observational studies" OR "observational study" OR "retrospective cohort" OR "retrospective" OR "retrospective cohort stud*" OR "prospective cohort" OR "prospective" OR "prospective cohort stud*" OR "cohort" OR "randomized controlled trial" OR "randomized" OR "RCT" OR "randomized control" OR "randomized trial" OR "controlled" OR "quasi-randomized" OR "randomized studies" OR "randomized study")) AND (PY=(2014) OR PY=(2015) OR PY=(2016) OR PY=(2017) OR PY=(2018) OR PY=(2019) OR PY=(2020) OR PY=(2021) OR PY=(2022))

CINAHL	
Date conducted	6 April 2022

# of results	1,172
Final combined	((MH "Hardiness") OR (TI("positive childhood experience*" OR "childhood protective factor*" OR ("protective factor*" AND ("physical activity" OR "relig*" OR "substance use" OR "suicid*" OR “friend*” OR “sports” OR “hobby” OR “exercise” OR “native language” OR “culture” OR “attachment”)) OR "PCE" OR "CPF" OR "positive experiences*" OR "mitigating factor*" OR "buffer" OR "resilient*" OR "resilience factor" OR "flourish" OR "emotional stability" OR "self esteem" OR "self concept" OR "emotional regulation" OR "nurturing relation*" OR "stable relation*" OR "interpersonal connection*" OR "family warmth" OR "familial warmth" OR "familial support" OR "family support" OR "parent warmth" OR "parental warmth" OR "emotional support" OR "emotional growth" OR "instrumental support" OR "secure attachment" OR "family strength" OR "supportive famil*" OR "parent* strength*" OR "familial asset*" OR "family asset*" OR "developmental asset*" OR "secure relation*" OR "feeling safe" OR "close relation*" OR "social support" OR "best friend" OR "prosocial peer" OR "cultural belonging" OR "sense of belonging" OR "safe environment" OR "equitable environment" OR "living environment" OR "residential stability" OR "stable environment" OR "safe home" OR "safe school" OR "food availability" OR "access to food" OR "safe hous*" OR "safe neighborhood" OR "access to healthcare" OR "healthcare access" OR "behavioral health access" OR "access to education" OR "educational access" OR "education access" OR "parent supervision" OR "parental supervision" OR "adult supervision" OR "caregiver supervision" OR "green space" OR "greenspace" OR "natural space" OR "safe play" OR "social engagement" OR "civic engagement" OR "school engagement" OR "community engagement" OR "cultural engagement" OR "academic support" OR "community traditions" OR "compensatory experien*" OR "protective experien*" OR "positive youth development" OR "youth asset*" OR "internal asset*" OR "caring adult*" OR "parental support" OR "connectedness" OR "family connect*" OR "family cohesion" OR "parent* connect*" OR "familism" OR "friendship ties" OR "interpersonal relationship*" OR "social condition*" OR "supportive community" OR "parental monitoring" OR “mentor*” OR “promotive factor*” OR “positive relation*” OR “safe stable nurturing relationship” OR “SSNR” OR “benevolent childhood experien*” OR “BCE” OR “protective and compensatory experien*” OR “PACEs”) OR AB("positive childhood experience*" OR "childhood protective factor*" OR ("protective factor*" AND ("physical activity" OR "relig*" OR "substance use" OR "suicid*" OR “friend*” OR “sports” OR “hobby” OR “exercise” OR “native language” OR “culture” OR “attachment”)) OR "PCE" OR "CPF" OR "positive experiences*" OR "mitigating factor*" OR "buffer" OR "resilient*" OR "resilience factor" OR "flourish" OR "emotional stability" OR "self esteem" OR "self concept" OR "emotional regulation" OR "nurturing relation*" OR "stable relation*" OR "interpersonal connection*" OR "family warmth" OR "familial warmth" OR "familial support" OR "family support" OR "parent warmth" OR "parental warmth" OR "emotional support" OR "emotional growth" OR "instrumental support" OR "secure attachment" OR "family strength" OR "supportive famil*" OR "parent* strength*" OR "familial asset*" OR "family asset*" OR "developmental asset*" OR "secure relation*" OR "feeling safe" OR "close relation*" OR "social support" OR "best friend" OR "prosocial peer" OR "cultural belonging" OR "sense of belonging" OR "safe environment" OR "equitable environment" OR "living environment" OR "residential stability" OR "stable environment" OR "safe home" OR "safe school" OR "food availability" OR "access to food" OR "safe hous*" OR "safe neighborhood" OR "access to healthcare" OR "healthcare access" OR "behavioral health access" OR "access to education" OR "educational access" OR "education access" OR "parent supervision" OR "parental supervision" OR "adult supervision" OR "caregiver supervision" OR "green space" OR "greenspace" OR "natural space" OR "safe play" OR "social engagement" OR "civic engagement" OR "school engagement" OR "community engagement" OR "cultural engagement" OR "academic support" OR "community traditions" OR "compensatory experien*" OR "protective experien*" OR "positive youth development" OR "youth asset*" OR "internal asset*" OR "caring adult*" OR "parental support" OR "connectedness" OR "family connect*" OR "family cohesion" OR "parent* connect*" OR "familism" OR "friendship ties" OR "interpersonal relationship*" OR "social condition*" OR "supportive community" OR "parental monitoring" OR “mentor*” OR “promotive factor*” OR “positive relation*” OR “safe stable nurturing relationship” OR “SSNR” OR “benevolent childhood experien*” OR “BCE” OR “protective and compensatory experien*” OR “PACEs”)))) AND ((MH "Adolescent Health") OR (MH "Child Health") OR (MH "Family Health") OR (TI(“child health” OR “adolescent health” OR “social determinants of health” OR “family health” OR "physical health" OR "mental health" OR "emotional health" OR "psychological health" OR "violence victimization" OR "violence perpetration" OR "self harm" OR “health outcomes” OR “health indicators” OR “substance use disorder” OR “suicid*” OR “interpersonal violence” OR “self directed violence” OR “mental illness” OR “depression” OR “substance abuse” OR “substance use” OR “overdose” OR “intimate partner violence” OR “domestic violence” OR “domestic abuse” OR “anxiet*” OR “substance misuse” OR “drug poisoning” OR “alcohol misuse” OR “alcohol abuse” OR “behavioral health”) OR AB(“child health” OR “adolescent health” OR “social determinants of health” OR “family health” OR "physical health" OR "mental health" OR "emotional health" OR "psychological health" OR "violence victimization" OR "violence perpetration" OR "self harm" OR “health outcomes” OR “health indicators” OR “substance use disorder” OR “suicid*” OR “interpersonal violence” OR “self directed violence” OR “mental illness” OR “depression” OR “substance abuse” OR “substance use” OR “overdose” OR “intimate partner violence” OR “domestic violence” OR “domestic abuse” OR “anxiet*” OR “substance misuse” OR “drug poisoning” OR “alcohol misuse” OR “alcohol abuse” OR “behavioral health”))) AND ((MH "Child+") OR (MH "Adolescence+")OR (TI("child*" OR "kid" OR "kids" OR "girl*" OR "boy*" OR "teen*" OR "youth*" OR "youngster" OR "adolescent*" OR "preschool" OR "pre-school" OR "kindergarten*" OR "elementary school*" OR "junior high" OR "junior high school*" OR "middle school*" OR "high school*" OR "juvenile" OR "minor*") OR AB("child*" OR "kid" OR "kids" OR "girl*" OR "boy*" OR "teen*" OR "youth*" OR "youngster" OR "adolescent*" OR "preschool" OR "pre-school" OR "kindergarten*" OR "elementary school*" OR "junior high" OR "junior high school*" OR "middle school*" OR "high school*" OR "juvenile" OR "minor*"))) AND ((MH "United States+") OR (TI("United States" OR "States" OR "US" OR "U.S." OR "USA" OR "U.S.A." OR "America*" OR "Appalachia*" OR "Appalachian region" OR "Georgia" OR "Kentucky" OR "North Carolina" OR "Ohio" OR "South Carolina" OR "Tennessee" OR "Virginia" OR "West Virginia" OR "Great Lakes" OR "Great Lakes Region" OR "Illinois" OR "Indiana" OR "Michigan" OR "Minnesota" OR "New York" OR "Ohio" OR "Pennsylvania" OR "Wisconsin" OR "Delaware" OR "District of Columbia" OR "Washington DC" OR "Washington D.C." OR "Maryland" OR "New Jersey" OR "Midwest United States" OR "Midwestern United States" OR "Midwest States" OR "Midwestern States" OR "Iowa" OR "Kansas" OR "Missouri" OR "Nebraska" OR "Oklahoma"

OR "South Dakota" OR "Wisconsin" OR "North Dakota" OR "New England" OR "New England Region" OR "Connecticut" OR "Maine" OR "Massachusetts" OR "New Hampshire" OR "Rhode Island" OR "Vermont" OR "Northwest United States" OR "Northwestern United States" OR "Northwest States" OR "Northwestern States" OR "Idaho" OR "Montana" OR "Oregon" OR "Washington" OR "Wyoming" OR "Pacific States" OR "Alaska" OR "California" OR "Hawaii" OR "Hawai'i" OR "Southeast United States" OR "Southeastern United States" OR "Southeast States" OR "Southeastern States" OR "Alabama" OR "Arkansas" OR "Florida" OR "Georgia" OR "Louisiana" OR "Mississippi" OR "Southwest United States" OR "Southwestern United States" OR "Southwest States" OR "Southwestern States" OR "Arizona" OR "Colorado" OR "Nevada" OR "New Mexico" OR "Texas" OR "Utah" OR "Chicago" OR "New York City" OR "Baltimore" OR "Philadelphia" OR "Boston" OR "Los Angeles" OR "San Francisco" OR "New Orleans") OR AB("United States" OR "States" OR "US" OR "U.S." OR "USA" OR "U.S.A." OR "America*" OR "Appalachia*" OR "Appalachian region" OR "Georgia" OR "Kentucky" OR "North Carolina" OR "Ohio" OR "South Carolina" OR "Tennessee" OR "Virginia" OR "West Virginia" OR "Great Lakes" OR "Great Lakes Region" OR "Illinois" OR "Indiana" OR "Michigan" OR "Minnesota" OR "New York" OR "Ohio" OR "Pennsylvania" OR "Wisconsin" OR "Delaware" OR "District of Columbia" OR "Washington DC" OR "Washington D.C." OR "Maryland" OR "New Jersey" OR "Midwest United States" OR "Midwestern United States" OR "Midwest States" OR "Midwestern States" OR "Iowa" OR "Kansas" OR "Missouri" OR "Nebraska" OR "Oklahoma" OR "South Dakota" OR "Wisconsin" OR "North Dakota" OR "New England" OR "New England Region" OR "Connecticut" OR "Maine" OR "Massachusetts" OR "New Hampshire" OR "Rhode Island" OR "Vermont" OR "Northwest United States" OR "Northwestern United States" OR "Northwest States" OR "Northwestern States" OR "Idaho" OR "Montana" OR "Oregon" OR "Washington" OR "Wyoming" OR "Pacific States" OR "Alaska" OR "California" OR "Hawaii" OR "Hawai'i" OR "Southeast United States" OR "Southeastern United States" OR "Southeast States" OR "Southeastern States" OR "Alabama" OR "Arkansas" OR "Florida" OR "Georgia" OR "Louisiana" OR "Mississippi" OR "Southwest United States" OR "Southwestern United States" OR "Southwest States" OR "Southwestern States" OR "Arizona" OR "Colorado" OR "Nevada" OR "New Mexico" OR "Texas" OR "Utah" OR "Chicago" OR "New York City" OR "Baltimore" OR "Philadelphia" OR "Boston" OR "Los Angeles" OR "San Francisco" OR "New Orleans"))) AND ((MH "Prospective Studies+") OR (MH "Cross Sectional Studies") OR (MH "Surveys+") OR (MH "Systematic Review") OR (MH "Meta Analysis") OR (MH "Randomized Controlled Trials+") OR (TI("meta analyses" OR "meta analysis" OR "meta analyz*" OR "meta analytic" OR "systematic reviews" OR "systematic" OR "cross sectional stud*" OR "cross sectional" OR "survey" OR "cohort studies" OR "cohort studios" OR "cohort study" OR "observational studies" OR "observational study" OR "retrospective cohort" OR "retrospective" OR "retrospective cohort stud*" OR "prospective cohort" OR "prospective" OR "prospective cohort stud*" OR "cohort" OR "randomized controlled trial" OR "randomized" OR "RCT" OR "randomized control" OR "randomized trial" OR "controlled" OR "quasi-randomized" OR "randomized studies" OR "randomized study") OR AB("meta analyses" OR "meta analysis" OR "meta analyz*" OR "meta analytic" OR "systematic reviews" OR "systematic" OR "cross sectional stud*" OR "cross sectional" OR "survey" OR "cohort studies" OR "cohort studios" OR "cohort study" OR "observational studies" OR "observational study" OR "retrospective cohort" OR "retrospective" OR "retrospective cohort stud*" OR "prospective cohort" OR "prospective" OR "prospective cohort stud*" OR "cohort" OR "randomized controlled trial" OR "randomized" OR "RCT" OR "randomized control" OR "randomized trial" OR "controlled" OR "quasi-randomized" OR "randomized studies" OR "randomized study"))) AND ((DT 2014) OR (DT 2015) OR (DT 2016) OR (DT 2017) OR (DT 2018) OR (DT 2019) OR (DT 2020) OR (DT 2021) OR (DT 2022))

File S4. Included references

Abbott, Marissa, and Kristen S. Slack, "Exploring the relationship between childhood adversity and adult depression: A risk versus strengths-oriented approach," Child abuse & neglect, Vol. 120, Oct 2021, 2021, p. 1.

Abraczinskas, M., R. Kilmer, M. Haber, J. Cook, and N. Zarrett, "Effects of Extracurricular Participation on the Internalizing Problems and Intrapersonal Strengths of Youth in a System of Care," Am J Community Psychol, Vol. 57, No. 3-4, Jun, 2016, pp. 308-319.

Affrunti, N. W., L. Suárez, and D. Simpson, "Community violence and posttraumatic stress disorder symptoms in urban youth: The moderating influence of friend and parent support," Journal of community psychology, Vol. 46, No. 5, 2018, pp. 636-650.

Aguayo, L., L. B. Beach, X. Wang, M. M. Ruprecht, D. Felt, K. N. Kershaw, M. M. Davis, and G. Phillips, "Someone to talk to: the association of mentorship and cyberbullying with suicidality among US high school students," Social psychiatry and psychiatric epidemiology, Vol. 57, No. 1, 2022, pp. 149-159.

Agyemang, D. O., E. F. Madden, K. English, K. L. Venner, R. Handy, T. P. Singh, and F. Qeadan, "The mediation and moderation effect of social support on the relationship between opioid misuse and suicide attempts among native American youth in New Mexico: 2009-2019 Youth Risk Resiliency Survey (NM-YRRS)," BMC Psychiatry, Vol. 22, No. 1, Apr 5, 2022, p. 243.

Alleyne-Green, Binta, Alex Kulick, Hadiza L. Osuji, Nisha Beharie, and Yvette Sealy, "The Impact of Shelter Environment, Parental Communication, and Supervision on Depression Outcomes Among an Urban Sample of Adolescent First-Time Shelter Users in New York City," Journal of Family Issues, Vol. 39, No. 11, Aug 2018, 2018, pp. 3075-3095.

Anderson, A. S., R. E. Siciliano, L. M. Henry, K. H. Watson, M. A. Gruhn, T. M. Kuhn, J. Ebert, A. J. Vreeland, A. E. Ciriegio, C. Guthrie, and B. E. Compas, "Adverse childhood experiences, parenting, and socioeconomic status: Associations with internalizing and externalizing symptoms in adolescence," Child Abuse and Neglect, Vol. 125, 2022.

Andersson, Matthew A., "The Long Arm of Warm Parenting: A Sex-Matching Perspective on Adult Children's Physical Health," Journal of Family Issues, Vol. 37, No. 7, 2016, pp. 879-901.

Arango, A., Y. Cole-Lewis, R. Lindsay, C. E. Yeguez, M. Clark, and C. King, "The Protective Role of Connectedness on Depression and Suicidal Ideation Among Bully Victimized Youth," J Clin Child Adolesc Psychol, Vol. 48, No. 5, Sep-Oct, 2019, pp. 728-739.

Areba, Eunice M., Lindsay A. Taliaferro, Myriam Forster, Barbara J. McMorris, Michelle A. Mathiason, and Marla E. Eisenberg, "Adverse childhood experiences and suicidality: school connectedness as a protective factor for ethnic minority adolescents," Children & Youth Services Review, Vol. 120, 2021, pp. N.PAG-N.PAG.

Arora, Prema G., Sycarah Fisher, Lorey A. Wheeler, Jessica Barnes, and Prerna G. Arora, "A prospective examination of anxiety as a predictor of depressive symptoms among Asian American early adolescent youth: The role of parent, peer, and teacher support and school engagement," Cultural Diversity & Ethnic Minority Psychology, Vol. 23, No. 4, 2017, pp. 541-550.

Atherton, O. E., R. D. Conger, E. Ferrer, and R. W. Robins, "Risk and Protective Factors for Early Substance Use Initiation: A Longitudinal Study of Mexican-Origin Youth," J Res Adolesc, Vol. 26, No. 4, Dec, 2016, pp. 864-879.

Ayers, Stephanie, Justin Jager, and Stephen S. Kulis, "Variations in risk and promotive factors on substance use among urban American Indian youth," Journal of Ethnicity in Substance Abuse, Vol. 20, No. 2, 2021, pp. 187-210.

Ballard, Parissa J., Lindsay T. Hoyt, and Mark C. Pachucki, "Impacts of Adolescent and Young Adult Civic Engagement on Health and Socioeconomic Status in Adulthood," Child Development, Vol. 90, No. 4, 2019, pp. 1138-1154.

Bennefield, Zinobia C., "Disadvantage Begets Disadvantage? Exploring Mental Health Pathways for Girls of Color," Research in the Sociology of Health Care, Vol. 37, 2019, 2019, pp. 15-32.

Bennett, M. Daniel, and Sean Joe, "Exposure to Community Violence, Suicidality, and Psychological Distress Among African American and Latino Youths: Findings From the CDC Youth Violence Survey," Journal of Human Behavior in the Social Environment, Vol. 25, No. 8, 2015, pp. 775-789.

Bethell, Christina, Jennifer Jones, Narangerel Gombojav, Jeff Linkenbach, and Robert Sege, "Positive Childhood Experiences and Adult Mental and Relational Health in a Statewide Sample: Associations Across Adverse Childhood Experiences Levels," JAMA Pediatrics, Vol. 173, No. 11, 2019, pp. e193007-e193007.

Bleck, J., and R. DeBate, "Long-Term Association Between Developmental Assets and Health Behaviors: An Exploratory Study," Health Educ Behav, Vol. 43, No. 5, Oct, 2016, pp. 543-551.

Boyas, Javier F., Yi Jin Kim, Tatiana Villarreal-Otálora, and J. Kenneth Sink, "Suicide ideation among Latinx adolescents: Examining the role of parental monitoring and intrinsic religiosity," Children & Youth Services Review, Vol. 102, 2019, pp. 177-185.

Bradley, Robert H., "Home Life and Health Among Native American, African American, and Latino Adolescents," Health Psychology, Vol. 38, No. 8, 2019, pp. 738-747.

Brinkman, Jesse, Bernice Garnett, Jane Kolodinsky, Weiwei Wang, and Lizzy Pope, "Intra-and Interpersonal Factors Buffer the Relationship Between Food Insecurity and Mental Well-Being Among Middle Schoolers," Journal of School Health, Vol. 91, No. 2, 2021, pp. 102-110.

Brooks-Russell, A., K. P. Conway, D. Liu, Y. Xie, G. C. Vullo, K. Li, R. J. Iannotti, W. Compton, and B. Simons-Morton, "Dynamic Patterns of Adolescent Substance Use: Results From a Nationally Representative Sample of High School Students," J Stud Alcohol Drugs, Vol. 76, No. 6, Nov, 2015, pp. 962-970.

Brown, S. M., and A. M. Shillington, "Childhood adversity and the risk of substance use and delinquency: The role of protective adult relationships," Child Abuse and Neglect, Vol. 63, 2017, pp. 211-221.

Chen, M. S., and V. A. Foshee, "Stressful life events and the perpetration of adolescent dating abuse," Journal of youth and adolescence, Vol. 44, No. 3, 2015, pp. 696-707.

Chen, Ping, and Kathleen Mullan Harris, "Association of Positive Family Relationships With Mental Health Trajectories From Adolescence to Midlife," JAMA Pediatrics, Vol. 173, No. 12, 2019, pp. e193336-e193336.

Cheng, T. C., and C. C. Lo, "Social Risk and Protective Factors in Adolescents' Reduction and Cessation of Alcohol Use," Subst Use Misuse, Vol. 52, No. 7, Jun 7, 2017, pp. 916-928.

Chopik, William J., and Robin S. Edelstein, "Retrospective memories of parental care and health from mid- to late life," Health Psychology, Vol. 38, No. 1, 2019, pp. 84-93.

Chung-Do, J. J., D. A. Goebert, F. Hamagani, J. Y. Chang, and E. S. Hishinuma, "Understanding the Role of School Connectedness and Its Association With Violent Attitudes and Behaviors Among an Ethnically Diverse Sample of Youth," Journal of Interpersonal Violence, Vol. 32, No. 9, 2017, pp. 1421-1446.

Clark, D. Angus, M. Brent Donnellan, Richard W. Robins, and Rand D. Conger, "Early adolescent temperament, parental monitoring, and substance use in Mexican-origin adolescents," Journal of Adolescence, Vol. 41, 2015, pp. 121-130.

Conner, K. R., P. Wyman, D. B. Goldston, R. M. Bossarte, N. Lu, K. Kaukeinen, X. M. Tu, R. J. Houston, D. A. Lamis, G. Chan, K. K. Bucholz, and V. M. Hesselbrock, "Two Studies of Connectedness to Parents and Suicidal Thoughts and Behavior in Children and Adolescents," J Clin Child Adolesc Psychol, Vol. 45, No. 2, 2016, pp. 129-140.

Crandall, A., B. M. Magnusson, C. L. Hanson, and B. Leavitt, "The effects of adverse and advantageous childhood experiences on adult health in a low-income sample," Acta psychologica, Vol. 220, 2021, p. 103430.

Crisanti, A. S., D. Duran, R. N. Greene, J. Reno, C. Luna-Anderson, and D. B. Altschul, "A longitudinal analysis of peer-delivered permanent supportive housing: Impact of housing on mental and overall health in an ethnically diverse population," Psychol Serv, Vol. 14, No. 2, May, 2017, pp. 141-153.

Cruz, Rick A., Kevin M. King, Ana M. Cauce, Rand D. Conger, and Richard W. Robins, "Cultural Orientation Trajectories and Substance Use: Findings From a Longitudinal Study of Mexican-Origin Youth," Child Development, Vol. 88, No. 2, 2017, pp. 555-572.

Cruz, R. A., K. M. King, M. Mechammil, M. Bámaca-Colbert, and R. W. Robins, "Mexican-origin youth substance use trajectories: Associations with cultural and family factors," Dev Psychol, Vol. 54, No. 1, Jan, 2018, pp. 111-126.

Culyba, Alison J., Elizabeth Miller, Steven M. Albert, and Kaleab Z. Abebe, "Co-occurrence of Violence-Related Risk and Protective Behaviors and Adult Support Among Male Youth in Urban Neighborhoods," JAMA Network Open, Vol. 2, No. 9, 2019, pp. e1911375-e1911375.

De Luca, S. M., C. Caramanis, and A. Zhang, "A longitudinal study examining the associations of bullying victimization and suicidal ideation among sexual minority adolescents," Suicide Life Threat Behav, Vol. 51, No. 6, Dec, 2021, pp. 1138-1147.

De Pedro, Kris Tunac, Monica Christina Esqueda, and Tamika D. Gilreath, "School Protective Factors and Substance Use Among Lesbian, Gay, and Bisexual Adolescents in California Public Schools," LGBT Health, Vol. 4, No. 3, 2017, pp. 210-216.

De Pedro, Kris Tunac, and Michael Morgan Gorse, "Substance use among transgender youth: Associations with school-based victimization and school protective factors," Journal of LGBT Youth, Jan 13, 2022, 2022.

Demir-Dagdas, Tuba, "Childhood disadvantage, cigarette smoking, and mental health: a mediation effect of social engagement," Vulnerable Children & Youth Studies, Vol. 15, No. 1, 2020, pp. 13-20.

Donaldson, Candice D., Brandon Nakawaki, and William D. Crano, "Variations in parental monitoring and predictions of adolescent prescription opioid and stimulant misuse," Addictive Behaviors, Vol. 45, 2015, pp. 14-21.

Doom, J. R., D. Seok, A. J. Narayan, and K. R. Fox, "Adverse and Benevolent Childhood Experiences Predict Mental Health During the COVID-19 Pandemic," Advers Resil Sci, Vol. 2, No. 3, 2021, pp. 193-204.

Doyle, David Matthew, Pam Factor-Litvak, and Bruce G. Link, "Modeling racial disparities in physical health via close relationship functioning: A life course approach," Social Science & Medicine, Vol. 204, 2018, pp. 31-38.

Drevon, D. D., E. P. Almazan, S. Jacob, and K. N. Rhymer, "Impact of Mentors During Adolescence on Outcomes Among Gay Young Adults," J Homosex, Vol. 63, No. 6, Jun, 2016, pp. 821-837.

Dubowitz, H., S. Roesch, R. Metzger, A. M. Arria, R. Thompson, and D. English, "Child Maltreatment, Relationship with Father, Peer Substance Use, and Adolescent Marijuana Use," J Child Adolesc Subst Abuse, Vol. 28, No. 3, 2019, pp. 150-159.

Dudovitz, Rebecca N., Paul J. Chung, and Mitchell D. Wong, "Teachers and Coaches in Adolescent Social Networks Are Associated With Healthier Self-Concept and Decreased Substance Use," Journal of School Health, Vol. 87, No. 1, 2017, pp. 12-20.

Dunn, M. S., "Association between physical activity and substance USE behaviors among high school students participating in the 2009 youth risk behavior survey," Psychological Reports, Vol. 114, No. 3, 2014, pp. 675-685.

Earnest, A. A., and S. S. Brady, "Dating Violence Victimization Among High School Students in Minnesota: Associations With Family Violence, Unsafe Schools, and Resources for Support," Journal of Interpersonal Violence, Vol. 31, No. 3, 2016, pp. 383-406.

Earnshaw, Valerie A., Lisa Rosenthal, Amy Carroll-Scott, Susan M. Peters, Catherine McCaslin, and Jeannette R. Ickovics, "Teacher involvement as a protective factor from the association between race-based bullying and smoking initiation," Social Psychology of Education: An International Journal, Vol. 17, No. 2, Jun 2014, 2014, pp. 197-209.

East, P. L., and A. Hokoda, "Risk and protective factors for sexual and dating violence victimization: a longitudinal, prospective study of Latino and African American adolescents," Journal of youth and adolescence, Vol. 44, No. 6, 2015, pp. 1288-1300.

Easterlin, M. C., P. J. Chung, M. Leng, and R. Dudovitz, "Association of Team Sports Participation with Long-term Mental Health Outcomes among Individuals Exposed to Adverse Childhood Experiences," JAMA Pediatrics, Vol. 173, No. 7, 2019, pp. 681-688.

Eiden, R. D., J. Lessard, C. R. Colder, J. Livingston, M. Casey, and K. E. Leonard, "Developmental cascade model for adolescent substance use from infancy to late adolescence," Dev Psychol, Vol. 52, No. 10, Oct, 2016, pp. 1619-1633.

Eisenberg, Marla E., Amy L. Gower, Barbara J. McMorris, G. Nicole Rider, and Eli Coleman, "Emotional Distress, Bullying Victimization, and Protective Factors Among Transgender and Gender Diverse Adolescents in City, Suburban, Town, and Rural Locations," Journal of Rural Health, Vol. 35, No. 2, 2019, pp. 270-281.

Elkins, S. R., P. J. Fite, T. M. Moore, J. E. Lochman, and K. C. Wells, "Bidirectional effects of parenting and youth substance use during the transition to middle and high school," Psychol Addict Behav, Vol. 28, No. 2, Jun, 2014, pp. 475-486.

Elmore, A. L., E. Crouch, and M. A. Kabir Chowdhury, "The Interaction of Adverse Childhood Experiences and Resiliency on the Outcome of Depression Among Children and Youth, 8-17 year olds," Child Abuse and Neglect, Vol. 107, 2020.

Escobar, O. S., and E. L. Vaughan, "Public religiosity, religious importance, and substance use among Latino emerging adults," Subst Use Misuse, Vol. 49, No. 10, Aug, 2014, pp. 1317-1325.

Espelage, D. L., R. W. Leemis, P. H. Niolon, M. Kearns, K. C. Basile, and J. P. Davis, "Teen Dating Violence Perpetration: Protective Factor Trajectories from Middle to High School among Adolescents," J Res Adolesc, Vol. 30, No. 1, Mar, 2020, pp. 170-188.

Ethier, K. A., C. R. Harper, and P. J. Dittus, "School Environment Is Related to Lower Health and Safety Risks Among Sexual Minority Middle and High School Students," J Adolesc Health, Vol. 62, No. 2, Feb, 2018, pp. 143-148.

Ewing, B. A., K. C. Osilla, E. R. Pedersen, S. B. Hunter, J. N. V. Miles, and E. J. D'Amico, "Longitudinal family effects on substance use among an at-risk adolescent sample," Addictive Behaviors, Vol. 41, 2015, pp. 185-191.

Fagan, A. A., E. M. Wright, and G. M. Pinchevsky, "The protective effects of neighborhood collective efficacy on adolescent substance use and violence following exposure to violence," J Youth Adolesc, Vol. 43, No. 9, Sep, 2014, pp. 1498-1512.

Fallin-Bennett, Amanda, and Amie Goodin, "Substance Use and School Characteristics in Lesbian, Gay, Bisexual, and Heterosexual High School Students," Journal of School Health, Vol. 89, No. 3, 2019, pp. 219-225.

Farrell, C. T., J. M. Bolland, and W. C. Cockerham, "The role of social support and social context on the incidence of attempted suicide among adolescents living in extremely impoverished communities," J Adolesc Health, Vol. 56, No. 1, Jan, 2015, pp. 59-65.

Feldman, Julia S., Yiyao Zhou, Chelsea Weaver Krug, Melvin N. Wilson, Kathryn Lemery-Chalfant, and Daniel S. Shaw, "Extracurricular involvement in the school-age period and adolescent problem behavior among low-income youth," Journal of Consulting and Clinical Psychology, Vol. 89, No. 11, Nov 2021, 2021, pp. 947-955.

Fish, J. N., B. S. Russell, R. J. Watson, and S. T. Russell, "Parent-child Relationships and Sexual Minority Youth: Implications for Adult Alcohol Abuse," Journal of youth and adolescence, Vol. 49, No. 10, 2020, pp. 2034-2046.

Fisher, S., L. A. Wheeler, P. G. Arora, J. Chaudry, and J. Barnes-Najor, "Ethnic identity and substance use in multiracial youth: the moderating role of support networks," Substance Use & Misuse, Vol. 54, No. 9, 2019, pp. 1417-1428.

Folk, J. B., L. K. Brown, B. D. L. Marshall, L. M. C. Ramos, L. Gopalakrishnan, D. Koinis-Mitchell, and M. Tolou-Shams, "The Prospective Impact of Family Functioning and Parenting Practices on Court-Involved Youth's Substance Use and Delinquent Behavior," Journal of youth and adolescence, Vol. 49, No. 1, 2020, pp. 238-251.

Forster, Myriam, Amy L. Gower, Iris W. Borowsky, and Barbara J. McMorris, "Associations between adverse childhood experiences, student-teacher relationships, and non-medical use of prescription medications among adolescents," Addictive Behaviors, Vol. 68, 2017, pp. 30-34.

Foshee, Vangie A., Luz McNaughton Reyes, Andra T. Tharp, Ling-Yin Chang, Susan T. Ennett, Thomas R. Simon, Natasha E. Latzman, and Chiravath Suchindran, "Shared Longitudinal Predictors of Physical Peer and Dating Violence," Journal of Adolescent Health, Vol. 56, No. 1, 2015, pp. 106-112.

Frenkel, T. I., N. A. Fox, D. S. Pine, O. L. Walker, K. A. Degnan, and A. Chronis-Tuscano, "Early childhood behavioral inhibition, adult psychopathology and the buffering effects of adolescent social networks: a twenty-year prospective study," Journal of child psychology and psychiatry, and allied disciplines, Vol. 56, No. 10, 2015, pp. 1065-1073.

Fulginiti, A., A. S. He, and S. Negriff, "Suicidal because I don't feel connected or vice versa? A longitudinal study of suicidal ideation and connectedness among child welfare youth," Child Abuse and Neglect, Vol. 86, 2018, pp. 278-289.

Fullerton, Lynne, Courtney A. FitzGerald, Meryn E. Hall, Dan Green, Lemyra M. DeBruyn, and Linda J. Peñaloza, "Suicide Attempt Resiliency in American Indian, Hispanic, and Anglo Youth in New Mexico: The Influence of Positive Adult Relationships," Family & Community Health, Vol. 42, No. 3, 2019, pp. 171-179.

Gaston, Shytierra, and Elaine Eggleston Doherty, "Why Don’t More Black Americans Offend? Testing a Theory of African American Offending’s Ethnic-Racial Socialization Hypothesis," Race and Justice, Vol. 8, No. 4, Oct 2018, 2018, pp. 366-395.

Genç, E., Y. Su, and J. Durtshi, "Moderating Factors Associated With Interrupting the Transmission of Domestic Violence Among Adolescents," J Interpers Violence, Vol. 36, No. 9-10, May, 2021, pp. Np5427-np5446.

Goldbach, J. T., S. M. Schrager, and I. W. Holloway, "The application of minority stress theory to binge drinking in lesbian and gay adolescents," Alcoholism: Clinical and Experimental Research, Vol. 38, 2014, p. 312A.

Goldstick, Jason E., Justin Heinze, Quyen Ngo, Hsing-Fang Hsieh, Maureen A. Walton, Rebecca M. Cunningham, and Marc A. Zimmerman, "Perceived Peer Behavior and Parental Support as Correlates of Marijuana Use: The Role of Age and Gender," Substance Use & Misuse, Vol. 53, No. 3, 2018, pp. 521-531.

Gonzales, Nancy A., Jessie J. Wong, Russell B. Toomey, Roger Millsap, Larry E. Dumka, and Anne M. Mauricio, "School engagement mediates long-term prevention effects for Mexican American adolescents," Prevention Science, Vol. 15, No. 6, 2014, pp. 929-939.

Goodrum, N. M., D. W. Smith, R. F. Hanson, A. D. Moreland, B. E. Saunders, and D. G. Kilpatrick, "Longitudinal Relations among Adolescent Risk Behavior, Family Cohesion, Violence Exposure, and Mental Health in a National Sample," Journal of Abnormal Child Psychology, Vol. 48, No. 11, 2020, pp. 1455-1469.

Goodwill, Janelle R., "Black Youth's Experiences With Feelings of Worthlessness, Parent Relationships, and Suicide: Findings From a National Probability Survey," Journal of Adolescent Health, Vol. 69, No. 2, 2021, pp. 294-301.

Gordon, M. S., B. S. Russell, and L. J. Finan, "The Influence of Parental Support and Community Belonging on Socioeconomic Status and Adolescent Substance Use over Time," Subst Use Misuse, Vol. 55, No. 1, 2020, pp. 23-36.

Gower, Amy L., G. Nic Rider, Camille Brown, Barbara J. McMorris, Eli Coleman, Lindsay A. Taliaferro, and Marla E. Eisenberg, "Supporting Transgender and Gender Diverse Youth: Protection Against Emotional Distress and Substance Use," American Journal of Preventive Medicine, Vol. 55, No. 6, 2018, pp. 787-794.

Greene, Naomi, Laura Tomedi, Jessica Reno, and Dan Green, "The Role of Substance Use and Resiliency Factors on Suicidal Ideation among Middle School Students," Journal of School Health, Vol. 90, No. 2, 2020, pp. 73-80.

Griffith, J. M., C. M. Crawford, C. W. Oppenheimer, J. F. Young, and B. L. Hankin, "Parenting and Youth Onset of Depression Across Three Years: Examining the Influence of Observed Parenting on Child and Adolescent Depressive Outcomes," J Abnorm Child Psychol, Vol. 47, No. 12, Dec, 2019, pp. 1969-1980.

Guttmannova, Katarina, Melissa J. Wheeler, Karl G. Hill, Teresa A. Evans-Campbell, Lacey A. Hartigan, Tiffany M. Jones, J. David Hawkins, and Richard F. Catalano, "Assessment of risk and protection in Native American youth: Steps toward conducting culturally relevant, sustainable prevention in Indian country," Journal of community psychology, Vol. 45, No. 3, Apr 2017, 2017, pp. 346-362.

Hall, M., L. Fullerton, D. Green, and C. A. Fitzgerald, "Positive relationships with adults and resilience to suicide attempt among new mexico hispanic adolescents," International Journal of Environmental Research and Public Health, Vol. 18, No. 19, 2021.

Hartley, K., J. Perazzo, C. Brokamp, G. L. Gillespie, K. M. Cecil, G. LeMasters, K. Yolton, and P. Ryan, "Residential surrounding greenness and self-reported symptoms of anxiety and depression in adolescents," Environmental Research, Vol. 194, 2021.

Hautala, Dane, and Kelley Sittner, "Moderators of the Association Between Exposure to Violence in Community, Family, and Dating Contexts and Substance Use Disorder Risk Among North American Indigenous Adolescents," Journal of Interpersonal Violence, Vol. 36, No. 9/10, 2021, pp. 4615-4640.

Heberle, A. E., S. C. Krill, M. J. Briggs-Gowan, and A. S. Carter, "Predicting externalizing and internalizing behavior in kindergarten: examining the buffering role of early social support," J Clin Child Adolesc Psychol, Vol. 44, No. 4, 2015, pp. 640-654.

Helms, S. W., M. Gallagher, C. D. Calhoun, S. Choukas-Bradley, G. C. Dawson, and M. J. Prinstein, "Intrinsic religiosity buffers the longitudinal effects of peer victimization on adolescent depressive symptoms," J Clin Child Adolesc Psychol, Vol. 44, No. 3, 2015, pp. 471-479.

Herrenkohl, T. I., H. Jung, J. Bart Klika, W. Alex Mason, E. C. Brown, R. T. Leeb, and R. C. Herrenkohl, "Mediating and moderating effects of social support in the study of child abuse and adult physical and mental health," American Journal of Orthopsychiatry, Vol. 86, No. 5, 2016, pp. 573-583.

Hirota, T., D. Paksarian, J. P. He, S. Inoue, E. K. Stapp, A. Van Meter, and K. R. Merikangas, "Associations of Social Capital with Mental Disorder Prevalence, Severity, and Comorbidity among U.S. Adolescents," Journal of clinical child and adolescent psychology : the official journal for the Society of Clinical Child and Adolescent Psychology, American Psychological Association, Division 53, 2021, pp. 1-12.

Horwitz, Adam G., Jacqueline Grupp-Phelan, David Brent, Bradley J. Barney, T. Charles Casper, Johnny Berona, Lauren S. Chernick, Rohit Shenoi, Mary Cwik, and Cheryl A. King, "Risk and protective factors for suicide among sexual minority youth seeking emergency medical services," Journal of Affective Disorders, Vol. 279, 2021, pp. 274-281.

Huang, C. Y., S. A. Nishioka, N. W. Zane, and P. Uchigakiuchi, "Examining Risk and Protective Predictors of Substance Use Among Low-Income Native Hawaiian and Pacific Islander Adolescents," American Journal of Orthopsychiatry, Vol. 92, No. 1, 2022, pp. 18-24.

Hurd, N. M., S. A. Stoddard, J. A. Bauermeister, and M. A. Zimmerman, "Natural mentors, mental health, and substance use: Exploring pathways via coping and purpose," Am J Orthopsychiatry, Vol. 84, No. 2, Mar, 2014, pp. 190-200.

Hurd, N. M., and M. A. Zimmerman, "An analysis of natural mentoring relationship profiles and associations with mentees' mental health: considering links via support from important others," Am J Community Psychol, Vol. 53, No. 1-2, Mar, 2014, pp. 25-36.

Ioverno, Salvatore, Alexander B. Belser, Roberto Baiocco, Arnold H. Grossman, and Stephen T. Russell, "The protective role of gay–straight alliances for lesbian, gay, bisexual, and questioning students: A prospective analysis," Psychology of Sexual Orientation and Gender Diversity, Vol. 3, No. 4, Dec 2016, 2016, pp. 397-406.

Janiri, D., G. E. Doucet, M. Pompili, G. Sani, B. Luna, D. A. Brent, and S. Frangou, "Risk and protective factors for childhood suicidality: a US population-based study," The Lancet Psychiatry, Vol. 7, No. 4, 2020, pp. 317-326.

Jimenez, M. P., E. Oken, D. R. Gold, H. Luttmann-Gibson, W. J. Requia, S. L. Rifas-Shiman, V. Gingras, M. F. Hivert, E. B. Rimm, and P. James, "Early life exposure to green space and insulin resistance: An assessment from infancy to early adolescence," Environment International, Vol. 142, 2020.

Kast, Nicole Rebecca, Marla E. Eisenberg, and Renee E. Sieving, "The Role of Parent Communication and Connectedness in Dating Violence Victimization among Latino Adolescents," Journal of Interpersonal Violence, Vol. 31, No. 10, 2016, pp. 1932-1955.

Katz-Wise, S. L., D. Ehrensaft, R. Vetters, M. Forcier, and S. B. Austin, "Family Functioning and Mental Health of Transgender and Gender-Nonconforming Youth in the Trans Teen and Family Narratives Project," Journal of sex research, Vol. 55, No. 4-5, 2018, pp. 582-590.

Katz-Wise, S. L., V. Sarda, S. Bryn Austin, and S. K. Harris, "Longitudinal effects of gender minority stressors on substance use and related risk and protective factors among gender minority adolescents," PLoS ONE, Vol. 16, No. 6 June, 2021.

Kent, B. V., and M. Bradshaw, "Adolescent Context and Depressive Symptom Trajectories in a National Sample: Ages 13 to 34," Int J Ment Health Addict, Vol. 19, No. 5, Oct, 2021, pp. 1468-1484.

Khetarpal, S. K., N. Szoko, A. J. Culyba, D. Shaw, and M. I. Ragavan, "Associations Between Parental Monitoring and Multiple Types of Youth Violence Victimization: A Brief Report," Journal of Interpersonal Violence, 2021, p. 8862605211035882.

Kim, J., E. Walsh, K. Pike, and E. A. Thompson, "Cyberbullying and Victimization and Youth Suicide Risk: The Buffering Effects of School Connectedness," The Journal of school nursing : the official publication of the National Association of School Nurses, Vol. 36, No. 4, 2020, pp. 251-257.

Kim, Yi Jin, Sung Seek Moon, Youn Kyoung Kim, and Javier Boyas, "Protective factors of suicide: Religiosity and parental monitoring," Children & Youth Services Review, Vol. 114, 2020, pp. N.PAG-N.PAG.

Kim, Y. J., C. R. Quinn, and S. S. Moon, "Buffering Effects of Social Support and Parental Monitoring on Suicide," Health & Social Work, Vol. 46, No. 1, 2021, pp. 42-50.

Kim-Spoon, J., J. P. Farley, C. Holmes, G. S. Longo, and M. E. McCullough, "Processes linking parents' and adolescents' religiousness and adolescent substance use: monitoring and self-control," J Youth Adolesc, Vol. 43, No. 5, May, 2014, pp. 745-756.

Kim-Spoon, J., J. P. Farley, C. J. Holmes, and G. S. Longo, "Does adolescents' religiousness moderate links between harsh parenting and adolescent substance use?," J Fam Psychol, Vol. 28, No. 6, Dec, 2014, pp. 739-748.

King, Cheryl A., Jacqueline Grupp-Phelan, David Brent, J. Michael Dean, Michael Webb, Jeffrey A. Bridge, Anthony Spirito, Lauren S. Chernick, E. Melinda Mahabee-Gittens, Rakesh D. Mistry, Margaret Rea, Allison Keller, Alexander Rogers, Rohit Shenoi, Mary Cwik, Danielle R. Busby, and T. Charles Casper, "Predicting 3-month risk for adolescent suicide attempts among pediatric emergency department patients," Journal of Child Psychology & Psychiatry, Vol. 60, No. 10, 2019, pp. 1055-1064.

Kirklewski, S. J., R. J. Watson, and C. Lauckner, "The moderating effect of physical activity on the relationship between bullying and mental health among sexual and gender minority youth," Journal of Sport and Health Science, 2021.

Kleiman, E. M., and R. T. Liu, "Prospective prediction of suicide in a nationally representative sample: religious service attendance as a protective factor," Br J Psychiatry, Vol. 204, 2014, pp. 262-266.

Kuramoto-Crawford, S. Janet, Mir M. Ali, and Holly C. Wilcox, "Parent-Child Connectedness and Long-Term Risk for Suicidal Ideation in a Nationally Representative Sample of US Adolescents," Crisis: The Journal of Crisis Intervention & Suicide Prevention, 2016, pp. 1-10.

Lardier, David T., Veronica R. Barrios, Pauline Garcia-Reid, and Robert J. Reid, "Preventing Substance Use Among Hispanic Urban Youth: Valuing the Role of Family, Social Support Networks, School Importance, and Community Engagement," Journal of Child & Adolescent Substance Abuse, Vol. 27, No. 5/6, 2018, pp. 251-263.

Lawrence, T. I., "Parental support, marital conflict, and stress as predictors of depressive symptoms among African American adolescents," Clin Child Psychol Psychiatry, Feb 24, 2022, p. 13591045211070163.

Lee, Jung Yeon, Wonkuk Kim, Judith S. Brook, Stephen J. Finch, and David W. Brook, "Adolescent Risk and Protective Factors Predicting Triple Trajectories of Substance Use from Adolescence into Adulthood," Journal of Child & Family Studies, Vol. 29, No. 2, 2020, pp. 403-412.

Lei, M. K., M. T. Berg, R. L. Simons, L. G. Simons, and S. R. H. Beach, "Childhood adversity and cardiovascular disease risk: An appraisal of recall methods with a focus on stress-buffering processes in childhood and adulthood," Social Science and Medicine, Vol. 246, 2020.

Lei, M. K., J. A. Lavner, S. E. Carter, A. R. Hart, and S. R. H. Beach, "Protective parenting behavior buffers the impact of racial discrimination on depression among Black youth," J Fam Psychol, Vol. 35, No. 4, Jun, 2021, pp. 457-467.

Lei, Man-Kit, Mark T. Berg, Ronald L. Simons, Leslie G. Simons, and Steven R. H. Beach, "Childhood adversity and cardiovascular disease risk: An appraisal of recall methods with a focus on stress-buffering processes in childhood and adulthood," Social Science & Medicine, Vol. 246, 2020, pp. N.PAG-N.PAG.

Lensch, T., K. Clements-Nolle, R. F. Oman, W. P. Evans, M. Lu, and W. Yang, "Adverse childhood experiences and co-occurring psychological distress and substance abuse among juvenile offenders: the role of protective factors," Public Health (Elsevier), Vol. 194, 2021, pp. 42-47.

———, "Adverse Childhood Experiences and Suicidal Behaviors Among Youth: The Buffering Influence of Family Communication and School Connectedness," Journal of Adolescent Health, Vol. 68, No. 5, 2021, pp. 945-952.

Lensch, T., K. Clements-Nolle, R. F. Oman, M. Lu, and W. P. Evans, "Prospective relationships between youth assets, negative life events, and binge drinking in a longitudinal cohort of the youth," Ann Epidemiol, Vol. 46, Jun, 2020, pp. 24-30.

Lipari, Rachel, Lori-Ann Palen, Olivia Silber Ashley, Michael Penne, Marni Kan, and Michael Pemberton, "Examination of Veteran Fathers' Parenting and Their Adolescent Children's Substance Use in the United States," Substance Use & Misuse, Vol. 52, No. 6, 2017, pp. 698-708.

Liu, Q., L. Mo, X. Huang, L. Yu, and Y. Liu, "Path analysis of the effects of social support, self-efficacy, and coping style on psychological stress in children with malignant tumor during treatment," Medicine (Baltimore), Vol. 99, No. 43, Oct 23, 2020, p. e22888.

Lorenzo-Blanco, Elma I., Seth J. Schwartz, Jennifer B. Unger, Andrea J. Romero, Miguel Ángel Cano, Lourdes Baezconde-Garbanati, David Córdova, Assaf Oshri, Daniel A. Santisteban, Sabrina E. Des Rosiers, Shi Huang, Juan A. Villamar, Daniel Soto, and Monica Pattarroyo, "A process-oriented analysis of parent acculturation, parent socio-cultural stress, family processes, and Latina/o youth smoking and depressive symptoms," International Journal of Intercultural Relations, Vol. 52, 2016, pp. 60-71.

Ma, Mindy, Lydia R. Malcolm, Kristine Díaz-Albertini, Juan Carlos Sánchez, Brett Simpson, Lissette Cortes, and Jeffrey L. Kibler, "Cultural Assets and Substance Use Among Hispanic Adolescents," Health Education & Behavior, Vol. 44, No. 2, 2017, pp. 326-331.

Mackin, D. M., G. Perlman, J. Davila, R. Kotov, and D. N. Klein, "Social support buffers the effect of interpersonal life stress on suicidal ideation and self-injury during adolescence," Psychological Medicine, Vol. 47, No. 6, 2017, pp. 1149-1161.

MacPherson, H. A., J. Wolff, B. Nestor, E. Frazier, M. Massing-Schaffer, H. Graves, C. Esposito-Smythers, and A. Spirito, "Parental Monitoring Predicts Depressive Symptom and Suicidal Ideation Outcomes in Adolescents Being Treated for Co-Occurring Substance Use and Psychiatric Disorders," Journal of Affective Disorders, Vol. 284, 2021, pp. 190-198.

Mak, H. W., G. M. Fosco, and S. T. Lanza, "Dynamic Associations of Parent-Adolescent Closeness and Friend Support With Adolescent Depressive Symptoms Across Ages 12-19," J Res Adolesc, Vol. 31, No. 2, Jun, 2021, pp. 299-316.

Mak, H. W., M. A. Russell, S. T. Lanza, M. E. Feinberg, and G. M. Fosco, "Age-varying associations of parental knowledge and antisocial peer behavior with adolescent substance use," Dev Psychol, Vol. 56, No. 2, Feb, 2020, pp. 298-311.

Manczak, E. M., S. J. Ordaz, M. K. Singh, M. S. Goyer, and I. H. Gotlib, "Time Spent with Parents Predicts Change in Depressive Symptoms in Adolescents with Major Depressive Disorder," J Abnorm Child Psychol, Vol. 47, No. 8, Aug, 2019, pp. 1401-1408.

Markowitz, A. J., "Associations Between School Connection and Depressive Symptoms From Adolescence Through Early Adulthood: Moderation by Early Adversity," J Res Adolesc, Vol. 27, No. 2, Jun, 2017, pp. 298-311.

Martin, Monica J., Kelly B. Bacher, Rand D. Conger, and Richard W. Robins, "Prospective Relationships Between Ethnic Discrimination and Substance Use by Mexican-American Adolescents," Child Development, Vol. 90, No. 6, 2019, pp. 2019-2034.

Martin, Monica J., Rand D. Conger, and Richard W. Robins, "Family Stress Processes and Drug and Alcohol Use by Mexican American Adolescents," Developmental Psychology, Vol. 55, No. 1, 2019, pp. 170-183.

Mason, M., J. Mennis, M. Russell, M. Moore, and A. Brown, "Adolescent Depression and Substance Use: the Protective Role of Prosocial Peer Behavior," J Abnorm Child Psychol, Vol. 47, No. 6, Jun, 2019, pp. 1065-1074.

Mason, M. J., and J. Mennis, "Young Urban Adolescents' Activity Spaces, Close Peers, and the Risk of Cannabis Use: A Social-Spatial Longitudinal Analysis," Subst Use Misuse, Vol. 53, No. 12, Oct 15, 2018, pp. 2032-2042.

Mauer, V. A., E. A. Waterman, K. M. Edwards, and V. L. Banyard, "Adolescents' Relationships With Important Adults: Exploring This Novel Protective Factor Against Interpersonal Violence Victimization and Perpetration," Journal of Interpersonal Violence, 2021, p. 8862605211031252.

McCallops, Kathleen, Ann M. Aviles, Valerie A. Earnshaw, and Rob Palkovitz, "Homelessness and suicidality: The role of bullying and parental support," Family Relations: An Interdisciplinary Journal of Applied Family Studies, Apr 15, 2020, 2020.

McNaughton Reyes, H. L., V. A. Foshee, N. C. Gottfredson, S. T. Ennett, and M. S. Chen, "Codevelopment of Delinquency, Alcohol Use, and Aggression Toward Peers and Dates: Multitrajectory Patterns and Predictors," Journal of research on adolescence : the official journal of the Society for Research on Adolescence, Vol. 30, No. 4, 2020, pp. 1025-1038.

McNeil, S. L., A. R. Andrews, and J. R. Cohen, "Emotional Maltreatment and Adolescent Depression: Mediating Mechanisms and Demographic Considerations in a Child Welfare Sample," Child Dev, Vol. 91, No. 5, Sep, 2020, pp. 1681-1697.

Metts, A. V., J. S. Yarrington, R. Zinbarg, C. Hammen, S. Mineka, C. Enders, and M. G. Craske, "Early-life adversity and risk for depression and anxiety: The role of interpersonal support," Development and psychopathology, 2022, pp. 1-13.

Meyer, Ilan H., Luo Feijun, Bianca D. M. Wilson, and Deborah M. Stone, "Sexual Orientation Enumeration in State Antibullying Statutes in the United States: Associations with Bullying, Suicidal Ideation, and Suicide Attempts Among Youth," LGBT Health, Vol. 6, No. 1, 2019, pp. 9-14.

Miles-McLean, H., M. Liss, and M. J. Erchull, "Fathers, daughters, and self-objectification: does bonding style matter?," Body Image, Vol. 11, No. 4, Sep, 2014, pp. 534-542.

Miller, A. B., L. M. Adams, C. Esposito-Smythers, R. Thompson, and L. J. Proctor, "Parents and friendships: a longitudinal examination of interpersonal mediators of the relationship between child maltreatment and suicidal ideation," Psychiatry Res, Vol. 220, No. 3, Dec 30, 2014, pp. 998-1006.

Miller, A. B., C. Esposito-Smythers, and R. N. Leichtweis, "Role of social support in adolescent suicidal ideation and suicide attempts," J Adolesc Health, Vol. 56, No. 3, Mar, 2015, pp. 286-292.

Miller, G. F., L. DePadilla, S. E. Jones, B. N. Bartholow, K. Sarmiento, and M. J. Breiding, "The Association Between Sports- or Physical Activity-Related Concussions and Suicidality Among US High School Students, 2017," Sports health, Vol. 13, No. 2, 2021, pp. 187-197.

Miller, R. N., A. A. Fagan, and E. M. Wright, "The Moderating Effects of Peer and Parental Support on the Relationship Between Vicarious Victimization and Substance Use," J Drug Issues, Vol. 44, No. 4, Oct, 2014, pp. 362-380.

Mintz, Sasha, Alberto Valido, Matthew Rivas-Koehl, Tomei Kuehl, Dorothy L. Espelage, Ashley Woolweaver, and Katherine M. Ingram, "Supporting Sexual Minority Youth: Protective Factors of Adverse Health Outcomes and Implications for Public Health," Journal of Adolescent Health, Vol. 69, No. 6, 2021, pp. 983-990.

Moon Sung, Seek, Jin Kim Yi, and Danielle Parrish, "Understanding the Linkages Between Parental Monitoring, School Academic Engagement, Substance Use, and Suicide Among Adolescents in U.S," Child & Youth Care Forum, Vol. 49, No. 6, Dec 2020, 2020, pp. 953-968.

Moreno, Oswaldo, Tim Janssen, Melissa J. Cox, Suzanne Colby, and Kristina M. Jackson, "Parent-adolescent relationships in Hispanic versus Caucasian families: Associations with alcohol and marijuana use onset," Addictive Behaviors, Vol. 74, 2017, pp. 74-81.

Mulla, M. M., K. W. Bogen, and L. M. Orchowski, "The mediating role of school connectedness in the associations between dating and sexual violence victimization and substance use among high school students," Preventive Medicine, Vol. 139, 2020.

Murphy, Sean M., and Robert Rosenman, "The "Real" Number of Washington State Adolescents Using Marijuana, and Why: A Misclassification Analysis," Substance Use & Misuse, Vol. 54, No. 1, 2019, 2019, p. 89.

Musliner, K. L., and J. B. Singer, "Emotional support and adult depression in survivors of childhood sexual abuse," Child Abuse Negl, Vol. 38, No. 8, Aug, 2014, pp. 1331-1340.

Oppenheimer, C. W., L. B. Stone, and B. L. Hankin, "The influence of family factors on time to suicidal ideation onsets during the adolescent developmental period," J Psychiatr Res, Vol. 104, Sep, 2018, pp. 72-77.

Palimaru, Alina I., Lu Dong, Ryan A. Brown, Elizabeth J. D'Amico, Daniel L. Dickerson, Carrie L. Johnson, and Wendy M. Troxel, "Mental health, family functioning, and sleep in cultural context among American Indian/Alaska Native urban youth: A mixed methods analysis," Social Science & Medicine, Vol. 292, 2022, pp. N.PAG-N.PAG.

Park, So-Young, Jungup Lee, and Charissa S. L. Cheah, "The long-term effects of perceived parental control and warmth on self-esteem and depressive symptoms among Asian American youth," Children & Youth Services Review, Vol. 126, 2021, pp. N.PAG-N.PAG.

Parks, M. J., J. Roesler, B. Menanteau, M. Raguet, and M. E. Eisenberg, "The Intersection of Depressive Symptoms, Adverse Childhood Experiences, and Protective Factors Among Adolescents: Epidemiological Evidence from Minnesota, 2016 and 2019," Advers Resil Sci, Jan 26, 2022, pp. 1-16.

Parmar, D. D., J. Tabler, M. J. Okumura, and J. M. Nagata, "Investigating Protective Factors Associated With Mental Health Outcomes in Sexual Minority Youth," J Adolesc Health, Vol. 70, No. 3, Mar, 2022, pp. 470-477.

Patel, Hima, Rachel Chambers, Shea Littlepage, Summer Rosenstock, Jennifer Richards, Angelita Lee, Anna Slimp, Laura Melgar, Shauntel Lee, Davette Susan, and Lauren Tingey, "The association of parental monitoring and parental communication with sexual and substance use risk behaviors among Native American Youth," Children & Youth Services Review, Vol. 129, 2021, pp. N.PAG-N.PAG.

Perreira, Krista M., Ashley N. Marchante, Seth J. Schwartz, Carmen R. Isasi, Mercedes R. Carnethon, Heather L. Corliss, Robert C. Kaplan, Daniel A. Santisteban, Denise C. Vidot, Linda Van Horn, and Alan M. Delamater, "Stress and Resilience: Key Correlates of Mental Health and Substance Use in the Hispanic Community Health Study of Latino Youth," Journal of Immigrant & Minority Health, Vol. 21, No. 1, 2019, pp. 4-13.

Peviani, K. M., A. Brieant, C. J. Holmes, B. King-Casas, and J. Kim-Spoon, "Religious Social Support Protects against Social Risks for Adolescent Substance Use," J Res Adolesc, Vol. 30, No. 2, Jun, 2020, pp. 361-371.

Piña-Watson, Brandy, Linda G. Castillo, Kimberly M. Rodriguez, and Sara Ray, "Familial factors related to suicidal ideation of latina adolescents in the United States," Archives of Suicide Research, Vol. 18, No. 2, 2014, pp. 213-220.

Pittenger, S. L., K. E. Moore, E. R. Dworkin, C. A. Crusto, and C. M. Connell, "Risk and Protective Factors for Alcohol, Marijuana, and Cocaine Use Among Child Welfare-Involved Youth," Child Youth Serv Rev, Vol. 95, Dec, 2018, pp. 88-94.

Porche, M. V., L. R. Fortuna, A. Wachholtz, and R. T. Stone, "Distal and Proximal Religiosity as Protective Factors for Adolescent and Emerging Adult Alcohol Use," Religions (Basel), Vol. 6, No. 2, 2015, pp. 365-384.

Quinn, Katherine, Jennifer L. Walsh, and Julia Dickson-Gomez, "Multiple Marginality and the Variation in Delinquency and Substance use Among Adolescent Gang Members," Substance Use & Misuse, Vol. 54, No. 4, 2019, pp. 612-627.

Ragavan, M. I., A. J. Culyba, D. Shaw, and E. Miller, "Social Support, Exposure to Parental Intimate Partner Violence, and Relationship Abuse Among Marginalized Youth," Journal of Adolescent Health, Vol. 67, No. 1, 2020, pp. 127-130.

Rallis, B. A., C. Esposito-Smythers, and R. Mehlenbeck, "Family environment as a moderator of the association between conduct disorder and suicidality," Journal of Aggression, Maltreatment and Trauma, Vol. 24, No. 2, 2015, pp. 150-168.

Reisner, S. L., K. Biello, N. S. Perry, K. E. Gamarel, and M. J. Mimiaga, "A compensatory model of risk and resilience applied to adolescent sexual orientation disparities in nonsuicidal self-injury and suicide attempts," The American journal of orthopsychiatry, Vol. 84, No. 5, 2014, pp. 545-556.

Richards, Tara N., Kathryn A. Branch, and Katherine Ray, "The impact of parental and peer social support on dating violence perpetration and victimization among female adolescents: a longitudinal study," Violence & Victims, Vol. 29, No. 2, 2014, pp. 317-331.

Rodriguez, K. E., S. E. McDonald, and S. M. Brown, "Relationships among Early Adversity, Positive Human and Animal Interactions, and Mental Health in Young Adults," Behav Sci (Basel), Vol. 11, No. 12, Dec 14, 2021.

Ross-Reed, D. E., J. Reno, L. Peñaloza, D. Green, and C. FitzGerald, "Family, School, and Peer Support Are Associated With Rates of Violence Victimization and Self-Harm Among Gender Minority and Cisgender Youth," Journal of Adolescent Health, Vol. 65, No. 6, 2019, pp. 776-783.

Ross-Reed, Danielle E., Jessica Reno, Linda Peñaloza, Dan Green, and Courtney FitzGerald, "Family, School, and Peer Support Are Associated With Rates of Violence Victimization and Self-Harm Among Gender Minority and Cisgender Youth...Head to Toe Conference, April 25, 2019, Albuquerque, New Mexico," Journal of Adolescent Health, Vol. 65, No. 6, 2019, pp. 776-783.

Rudolph, K. D., J. D. Monti, H. Modi, W. Y. Sze, and W. Troop-Gordon, "Protecting Youth Against the Adverse Effects of Peer Victimization: Why Do Parents Matter?," J Abnorm Child Psychol, Vol. 48, No. 2, Feb, 2020, pp. 163-176.

Rusby, Julie C., John M. Light, Ryann Crowley, and Erika Westling, "Influence of parent-youth relationship, parental monitoring, and parent substance use on adolescent substance use onset," Journal of Family Psychology, Vol. 32, No. 3, 2018, pp. 310-320.

Sabina, C., E. S. Marsical, and C. A. Cuevas, "Psychological Functioning Among Latino Victims of Teen Dating Violence: The Role of Relational and Collective Resources," Journal of Interpersonal Violence, 2021, p. 8862605211044097.

Santesteban-Echarri, O., M. A. Ramos-Olazagasti, R. E. Eisenberg, C. Wei, H. R. Bird, G. Canino, and C. S. Duarte, "Parental warmth and psychiatric disorders among Puerto Rican children in two different socio-cultural contexts," J Psychiatr Res, Vol. 87, Apr, 2017, pp. 30-36.

Sartor, C. E., A. E. Hipwell, and T. Chung, "Public and private religious involvement and initiation of alcohol, cigarette, and marijuana use in Black and White adolescent girls," Soc Psychiatry Psychiatr Epidemiol, Vol. 55, No. 4, Apr, 2020, pp. 447-456.

Sheidow, A. J., D. B. Henry, P. H. Tolan, and M. K. Strachan, "The Role of Stress Exposure and Family Functioning in Internalizing Outcomes of Urban Families," J Child Fam Stud, Vol. 23, No. 8, Nov, 2014, pp. 1351-1365.

Shetgiri, Rashmi, Denise Paquette Boots, Hua Lin, and Tina L. Cheng, "Predictors of Weapon-Related Behaviors among African American, Latino, and White Youth," Journal of Pediatrics, Vol. 171, 2016, pp. 277-282.

Shih, R. A., L. Parast, E. R. Pedersen, W. M. Troxel, J. S. Tucker, J. N. V. Miles, L. Kraus, and E. J. D'Amico, "Individual, peer, and family factor modification of neighborhood-level effects on adolescent alcohol, cigarette, e-cigarette, and marijuana use," Drug and Alcohol Dependence, Vol. 180, 2017, pp. 76-85.

Sigal, M., B. J. Ross, A. O. Behnke, and S. W. Plunkett, "Neighborhood, Peer, and Parental Influences on Minor and Major Substance Use of Latino and Black Adolescents," Children (Basel), Vol. 8, No. 4, Mar 31, 2021.

Simons-Morton, B., D. Haynie, D. Liu, A. Chaurasia, K. Li, and R. Hingson, "The Effect of Residence, School Status, Work Status, and Social Influence on the Prevalence of Alcohol Use Among Emerging Adults," Journal of Studies on Alcohol and Drugs, Vol. 77, No. 1, 2016, pp. 121-132.

Sloand, E., A. Butz, H. Rhee, L. Walters, K. Breuninger, R. A. Pozzo, C. M. Barnes, M. N. Wicks, and L. Tumiel-Berhalter, "Influence of social support on asthma self-management in adolescents," Journal of Asthma, Vol. 58, No. 3, 2021, pp. 386-394.

Slopen, Natalie, Ying Chen, Jennifer L. Guida, Michelle A. Albert, and David R. Williams, "Positive childhood experiences and ideal cardiovascular health in midlife: Associations and mediators," Preventive Medicine, Vol. 97, 2017, pp. 72-79.

Smith, Lia J., Chase Aycock, Kimberly Hook, Pan Chen, and Sandra Yu Rueger, "Parental Monitoring Moderates the Relation Between Radio Exposure and Adolescent Alcohol and Tobacco Use: Preliminary Findings From a National Survey," Journal of Child & Adolescent Substance Abuse, Vol. 26, No. 4, 2017, pp. 314-323.

Smokowski, P. R., M. L. Bacallao, K. L. Cotter, and C. B. Evans, "The effects of positive and negative parenting practices on adolescent mental health outcomes in a multicultural sample of rural youth," Child Psychiatry Hum Dev, Vol. 46, No. 3, Jun, 2015, pp. 333-345.

Smokowski, Paul R., Caroline B. R. Evans, Katie L. Cotter, and Shenyang Guo, "Ecological correlates of depression and self-esteem in rural youth," Child Psychiatry & Human Development, Vol. 45, No. 5, 2014, pp. 500-518.

Smokowski, P. R., S. Guo, C. B. Evans, Q. Wu, R. A. Rose, M. Bacallao, and K. L. Cotter, "Risk and protective factors across multiple microsystems associated with internalizing symptoms and aggressive behavior in rural adolescents: Modeling longitudinal trajectories from the Rural Adaptation Project," Am J Orthopsychiatry, Vol. 87, No. 1, 2017, pp. 94-108.

Smokowski, P. R., S. Guo, R. Rose, C. B. Evans, K. L. Cotter, and M. Bacallao, "Multilevel risk factors and developmental assets for internalizing symptoms and self-esteem in disadvantaged adolescents: modeling longitudinal trajectories from the Rural Adaptation Project," Dev Psychopathol, Vol. 26, No. 4 Pt 2, Nov, 2014, pp. 1495-1513.

So, Marvin, Nicole B. Perry, Adam D. Langenfeld, and Andrew J. Barnes, "Adolescent Sleep and Mental Health Across Race/Ethnicity: Does Parent-Child Connectedness Matter?," Journal of Developmental & Behavioral Pediatrics, Vol. 42, No. 9, 2021, pp. 742-750.

Spillane, N. S., M. R. Schick, K. T. Kirk-Provencher, D. C. Hill, J. Wyatt, and K. M. Jackson, "Structured and Unstructured Activities and Alcohol and Marijuana Use in Middle School: The Role of Availability and Engagement," Subst Use Misuse, Vol. 55, No. 11, 2020, pp. 1765-1773.

Spillane, Nichea S., Lisa Weyandt, Danielle Oster, and Hayley Treloar, "Social contextual risk factors for stimulant use among adolescent American Indians," Drug & Alcohol Dependence, Vol. 179, 2017, pp. 167-173.

Srivastav, Aditi, Rachel E. Davis, Melissa Strompolis, Elizabeth Crouch, James F. Thrasher, and Mindi Spencer, "Responding to Adverse Childhood Experiences: Understanding the Role of Safe, Stable, and Nurturing Relationships in Reducing Alcohol and Tobacco Related Risk Behaviors," Journal of Child & Adolescent Substance Abuse, Vol. 28, No. 6, Nov 2019, 2019, pp. 426-438.

Standley, C. J., and P. Foster-Fishman, "Intersectionality, social support, and youth suicidality: A socioecological approach to prevention," Suicide & Life-Threatening Behavior, Vol. 51, No. 2, 2021, pp. 203-211.

Steiner, R. J., G. Sheremenko, C. Lesesne, P. J. Dittus, R. E. Sieving, and K. A. Ethier, "Adolescent Connectedness and Adult Health Outcomes," Pediatrics, Vol. 144, No. 1, Jul, 2019.

Swaim, R. C., and L. R. Stanley, "Multivariate family factors in lifetime and current marijuana use among American Indian and white adolescents residing on or near reservations," Drug Alcohol Depend, Vol. 169, Dec 1, 2016, pp. 92-100.

Taliaferro, Lindsay A., Sung Tae Jang, Nicholas J. Westers, Jennifer J. Muehlenkamp, Janis L. Whitlock, and Barbara J. McMorris, "Associations between connections to parents and friends and non-suicidal self-injury among adolescents: The mediating role of developmental assets," Clinical Child Psychology & Psychiatry, Vol. 25, No. 2, 2020, pp. 359-371.

Taliaferro, Lindsay A., Barbara J. McMorris, G. Nicole Rider, and Marla E. Eisenberg, "Risk and Protective Factors for Self-Harm in a Population-Based Sample of Transgender Youth," Archives of Suicide Research, Vol. 23, No. 2, 2019, pp. 203-221.

Taliaferro, Lindsay A., and Jennifer J. Muehlenkamp, "Risk and Protective Factors that Distinguish Adolescents Who Attempt Suicide from Those Who Only Consider Suicide in the Past Year," Suicide & Life-Threatening Behavior, Vol. 44, No. 1, 2014, pp. 6-22.

———, "Nonsuicidal Self-Injury and Suicidality Among Sexual Minority Youth: Risk Factors and Protective Connectedness Factors," Academic Pediatrics, Vol. 17, No. 7, 2017, pp. 715-722.

Tingey, Lauren, Mary F. Cwik, Summer Rosenstock, Novalene Goklish, Francene Larzelere-Hinton, Angelita Lee, Rosemarie Suttle, Melanie Alchesay, Kirk Massey, and Allison Barlow, "Risk and protective factors for heavy binge alcohol use among American Indian adolescents utilizing emergency health services," American Journal of Drug & Alcohol Abuse, Vol. 42, No. 6, 2016, pp. 715-725.

Tsuchiya, K., D. B. Lee, Y. Qian, C. H. Caldwell, and R. B. Mincy, "Risk and protective family factors during childhood on youth violence among African American males: The role of mothers and nonresident fathers," J Community Psychol, Vol. 48, No. 5, Jul, 2020, pp. 1543-1563.

Turanovic, J. J., and T. C. Pratt, "Longitudinal effects of violent victimization during adolescence on adverse outcomes in adulthood: a focus on prosocial attachments," J Pediatr, Vol. 166, No. 4, Apr, 2015, pp. 1062-1069.e1061.

Van Meter, A. R., D. Paksarian, and K. R. Merikangas, "Social Functioning and Suicide Risk in a Community Sample of Adolescents," Journal of clinical child and adolescent psychology : the official journal for the Society of Clinical Child and Adolescent Psychology, American Psychological Association, Division 53, Vol. 48, No. 2, 2019, pp. 273-287.

Vaughan, Ellen L., Sylvia Martinez, Oscar S. Escobar, and Lisa K. Denton, "School Factors and Alcohol Use: The Moderating Effect of Nativity in a National Sample of Latino Adolescents," Substance Use & Misuse, Vol. 51, No. 6, 2016, pp. 742-751.

Vidourek, Rebecca A., Keith A. King, Michelle Burbage, and Barbara Okuley, "Impact of Parenting Behaviors on Recent Alcohol Use Among African American Students," Child & Adolescent Social Work Journal, Vol. 35, No. 3, 2018, pp. 271-282.

Vidourek, Rebecca A., Keith A. King, and LaTrice Montgomery, "Psychosocial determinants of marijuana use among African American youth," Journal of Ethnicity in Substance Abuse, Vol. 16, No. 1, 2017, pp. 43-65.

Voisin, Dexter R., Kelly M. King, Ralph J. Diclemente, and Monique Carry, "Correlates of gang involvement and health-related factors among African American females with a detention history," Children and Youth Services Review, Vol. 44, Sep 2014, 2014, pp. 120-125.

Wang, Cixin, Tamika La Salle, Chaorong Wu, Kieu Anh Do, and Kathryn E. Sullivan, "School climate and parental involvement buffer the risk of peer victimization on suicidal thoughts and behaviors among Asian American middle school students," Asian American Journal of Psychology, Vol. 9, No. 4, Dec 2018, 2018, pp. 296-307.

Wang, Cixin, Tanya Nieri, Kieu Anh Do, and Elizabeth Llanes, "Parenting Factors Predicting Substance Use and Aggression among Latino/a Adolescents: The Moderating Role of Cultural Values," Journal of Child & Family Studies, Vol. 29, No. 10, 2020, pp. 2710-2721.

Wang, M. T., and S. Sheikh-Khalil, "Does parental involvement matter for student achievement and mental health in high school?," Child Dev, Vol. 85, No. 2, Mar-Apr, 2014, pp. 610-625.

Weber Ku, E. B., M. A. Hagler, M. F. Parnes, S. E. O. Schwartz, J. E. Rhodes, and L. D. Erickson, "Natural mentoring relationships among survivors of caregiver childhood abuse: findings from the Add Health Study," Ann N Y Acad Sci, Vol. 1483, No. 1, Jan, 2021, pp. 50-66.

Wen, M., "Social Capital and Adolescent Substance Use: The Role of Family, School, and Neighborhood Contexts," Journal of research on adolescence : the official journal of the Society for Research on Adolescence, Vol. 27, No. 2, 2017, pp. 362-378.

Whitaker, Kelly, Valerie B. Shapiro, and John P. Shields, "School-Based Protective Factors Related to Suicide for Lesbian, Gay, and Bisexual Adolescents," Journal of Adolescent Health, Vol. 58, No. 1, 2016, pp. 63-68.

Whitesell, N. R., N. L. Asdigian, C. E. Kaufman, C. Big Crow, C. Shangreau, E. M. Keane, A. C. Mousseau, and C. M. Mitchell, "Trajectories of substance use among young American Indian adolescents: patterns and predictors," J Youth Adolesc, Vol. 43, No. 3, Mar, 2014, pp. 437-453.

Wilhelm, April K., Annie-Laurie McRee, Zobeida E. Bonilla, and Marla E. Eisenberg, "Mental health in Somali youth in the United States: the role of protective factors in preventing depressive symptoms, suicidality, and self-injury," Ethnicity & Health, Vol. 26, No. 4, 2021, pp. 530-553.

Wilkerson, J. M., V. R. Schick, K. A. Romijnders, J. Bauldry, and S. A. Butame, "Social Support, Depression, Self-Esteem, and Coping Among LGBTQ Adolescents Participating in Hatch Youth," Health Promotion Practice, Vol. 18, No. 3, 2017, pp. 358-365.

Wise, A. E., B. C. Smith, A. P. Armelie, J. M. Boarts, and D. L. Delahanty, "Age moderates the relationship between source of social support and mental health in racial minority lesbian, gay, and bisexual youth," Journal of Health Psychology, Vol. 24, No. 7, 2019, pp. 888-897.

Wolfe, J. D., "Maternal alcohol use disorders and depression in emerging adulthood: Examining the relevance of social ties, childhood adversity, and socioeconomic status," Psychiatry Res, Vol. 257, Nov, 2017, pp. 441-445.

Yang, Fang, Kit-Aun Tan, and Wendy J. Y. Cheng, "The effects of connectedness on health-promoting and health-compromising behaviors in adolescents: evidence from a statewide survey," Journal of Primary Prevention, Vol. 35, No. 1, 2014, pp. 33-46.

Yoon, Dalhee, "Peer-relationship patterns and their association with types of child abuse and adolescent risk behaviors among youth at-risk of maltreatment," Journal of Adolescence, Vol. 80, 2020, pp. 125-135.

Yoon, S., F. Pei, X. Wang, D. Yoon, G. Lee, K. Shockley McCarthy, and S. J. Schoppe-Sullivan, "Vulnerability or resilience to early substance use among adolescents at risk: The roles of maltreatment and father involvement," Child Abuse Negl, Vol. 86, Dec, 2018, pp. 206-216.

Zwald, Marissa L., Francis B. Annor, Amanda Wilkinson, Mike Friedrichs, Anna Fondario, Angela C. Dunn, Allyn Nakashima, Leah K. Gilbert, and Asha Ivey-Stephenson, "Suicidal Ideation and Attempts Among Students in Grades 8, 10, and 12 - Utah, 2015," MMWR: Morbidity & Mortality Weekly Report, Vol. 67, No. 15, 2018, pp. 451-454.

File S5. Summary table of studies reviewed

		Relationships										
		All Relationships Studies<br>N=139 (%)	Being securely attached to a parent or caregiver<br>N=4 (%)	Doing activities with caregiver<br>N=1 (%)	Familism and related family environments<br>N=4 (%)	Having positive peer relationships<br>N=28 (%)	Having prosocial peers<br>N=8 (%)	Love and support from a caregiver<br>N=66 (%)	Quality of relationship with caregiver<br>N=38 (%)	Social Support<br>N=18 (%)	Support from a teacher<br>N=14 (%)	Support from non-caregiver adult<br>N=15 (%)
Study type	Cross sectional	76 (54.7)	1 (25)	0 (0)	2 (50)	17 (60.7)	3 (37.5)	37 (56.1)	21 (55.3)	12 (66.7)	4 (28.6)	11 (73.3)
	Cohort	61 (43.9)	3 (75)	1 (100)	2 (50)	10 (35.7)	5 (62.5)	28 (42.4)	16 (42.1)	6 (33.3)	10 (71.4)	4 (26.7)
	Case control	2 (1.4)	0 (0)	0 (0)	0 (0)	1 (3.6)	0 (0)	1 (1.5)	1 (2.6)	0 (0)	0 (0)	0 (0)
	Randomized Control Trial	0 (0)	0 (0)	0 (0)	0 (0)	0 (0)	0 (0)	0 (0)	0 (0)	0 (0)	0 (0)	0 (0)
Outcome measures category*	Behavioral health	96 (49.0)	3 (75)	0 (0)	3 (75)	19 (67.9)	6 (75)	42 (63.6)	27 (71.1)	12 (66.7)	10 (71.4)	13 (86.7)
	Mental health	55 (28.1)	1 (25)	1 (100)	2 (50)	13 (46.4)	2 (25)	29 (43.9)	15 (39.5)	6 (33.3)	6 (42.9)	6 (40)
	Physical health	6 (3.1)	0 (0)	0 (0)	0 (0)	0 (0)	0 (0)	4 (6.1)	1 (2.6)	0 (0)	0 (0)	1 (6.7)
Study size	n < 1000	61 (43.9)	1 (25)	0 (0)	3 (75)	9 (32.1)	4 (50)	25 (37.9)	22 (57.9)	7 (38.9)	3 (21.4)	4 (26.7)
	n ≥ 1000	78 (56.1)	3 (75)	1 (100)	1 (25)	19 (67.9)	4 (50)	41 (62.1)	16 (42.1)	11 (61.1)	11 (78.6)	11 (73.3)
Outcome Age	Child Outcomes Only	109 (78.4)	1 (25)	0 (0)	4 (100)	23 (82.1)	6 (75)	56 (84.8)	29 (76.3)	15 (83.3)	13 (92.9)	10 (66.7)
	Includes Adult Outcomes	30 (21.6)	3 (75)	1 (100)	0 (0)	5 (17.9)	2 (25)	10 (15.2)	9 (23.7)	3 (16.7)	1 (7.1)	5 (33.3)

Data Source	Add Health	17	2 (50)	1 (100)	0 (0)	3 (10.7)	1 (12.5)	8 (12.1)	4 (10.5)	1 (5.6)	1 (7.1)	2 (13.3)
	Minnesota Student Survey	9	0 (0)	0 (0)	0 (0)	3 (10.7)	0 (0)	8 (12.1)	0 (0)	0 (0)	6 (42.9)	0 (0)
	New Mexico Youth Risk and Resilience Survey	6	0 (0)	0 (0)	0 (0)	4 (14.3)	0 (0)	4 (6.1)	0 (0)	1 (5.6)	0 (0)	3 (20)
	YRBS	6	0 (0)	0 (0)	0 (0)	0 (0)	0 (0)	2 (3.0)	1 (2.6)	0 (0)	1 (7.1)	2 (13.3)
	Other	59	2 (50)	0 (0)	1 (25)	14 (50)	6 (75)	25 (37.9)	19 (50)	10 (55.5)	5 (35.8)	3 (20)
	Not Reported	42	0 (0)	0 (0)	3 (75)	4 (14.3)	1 (12.5)	19 (28.8)	14 (36.9)	6 (33.3)	1 (7.1)	5 (33.4)

Environment							
		All Environment Studies N=93 (%)	Access to green spaces and playgrounds to play N=2 (%)	Having access to a protective school environment N=47 (%)	Living in a safe neighborhood or community N=9 (%)	Parental monitoring and related family environment N=42 (%)	Regular physical activity N=7 (%)
Study type	Cross sectional	51 (54.8)	0 (0)	32 (68.1)	5 (55.6)	22 (52.4)	5 (71.4)
	Cohort	40 (43.0)	2 (100)	14 (29.8)	4 (44.4)	19 (45.2)	2 (28.6)
	Case control	0 (0)	0 (0)	0 (0)	0 (0)	0 (0)	0 (0)
	Randomized Control Trial	2 (2.2)	0 (0)	1 (2.1)	0 (0)	1 (2.4)	0 (0)
Outcome measures category*	Behavioral health	78 (83.9)	0 (0)	40 (85.1)	7 (77.8)	39 (92.9)	4 (57.1)
	Mental health	22 (23.7)	1 (50)	13 (27.7)	3 (33.3)	4 (9.5)	3 (42.9)

	Physical health	1 (1.1)	1 (50)	0 (0)	0 (0)	0 (0)	0 (0)
Study size	n < 1000	35 (37.6)	2 (100)	14 (29.8)	0 (0)	19 (45.2)	0 (0)
	n ≥ 1000	58 (62.4)	0 (0)	33 (70.2)	9 (100)	23 (54.8)	7 (100)
Outcome Age	Child Outcomes Only	84 (90.3)	2 (100)	43 (91.5)	9 (100)	38 (90.5)	5 (71.4)
	Includes Adult Outcomes	9 (9.7)	0 (0)	4 (8.5)	0 (0)	4 (9.5)	2 (28.6)
Data Source	Add Health	10 (10.7)	0 (0)	6 (12.8)	2 (22.2)	2 (4.8)	2 (28.6)
	Minnesota Student Survey	4 (4.3)	0 (0)	3 (6.4)	2 (22.2)	0 (0)	1 (14.3)
	National Survey on Drug Use and Health	7 (7.5)	0 (0)	2 (4.2)	0 (0)	6 (14.3)	0 (0)
	New Mexico Youth Risk and Resilience Survey	5 (5.4)	0 (0)	3 (6.4)	0 (0)	3 (7.1)	0 (0)
	YRBS	5 (5.4)	0 (0)	3 (6.4)	0 (0)	0 (0)	3 (42.8)
	Other	33 (35.5)	2 (100)	17 (36.2)	4 (44.5)	12 (28.6)	1 (14.3)
	Not Reported	29 (31.2)	0 (0)	13 (27.6)	1 (11.1)	19 (45.2)	0 (0)

Engagement							
	All Engagement Studies N=46 (%)	Community connectedness N=9 (%)	For Indian/Native communities, native culture engagement	Having opportunities with constructive social engagement	Having opportunity for extracurricular	Having opportunity to have beliefs that give comfort	Sense of community or other cultural belonging

				N=1 (%)	and developing connectedness<br><br>N=3 (%)	engagement with school or community<br><br>N=16 (%)	N=10 (%)	N=11 (%)
Study type	Cross sectional	26 (56.5)	8 (88.9)	0 (0)	1 (33.3)	11 (68.8)	3 (30)	6 (54.5)
	Cohort	19 (41.3)	1 (11.1)	0 (0)	2 (66.7)	5 (31.2)	7 (70)	4 (36.4)
	Case control	1 (2.2)	0 (0)	1 (100)	0 (0)	0 (0)	0 (0)	1 (9.1)
	Randomized Control Trial	0 (0)	0 (0)	0 (0)	0 (0)	0 (0)	0 (0)	0 (0)
Outcome measures category*	Behavioral health	39 (84.8)	9 (100)	1 (100)	2 (66.7)	12 (75)	9 (90)	9 (81.8)
	Mental health	16 (34.8)	5 (55.6)	0 (0)	2 (66.7)	7 (43.8)	2 (20)	3 (27.3)
	Physical health	0 (0)	0 (0)	0 (0)	0 (0)	0 (0)	0 (0)	0 (0)
Study size	n < 1000	21 (45.7)	1 (11.1)	1 (100)	3 (100)	4 (25)	6 (60)	7 (63.6)
	n ≥ 1000	25 (54.3)	8 (88.9)	0 (0)	0 (0)	12 (75)	4 (40)	4 (36.4)
Outcome Age	Child Outcomes Only	36 (78.3)	9 (100)	1 (100)	1 (33.3)	15 (93.7)	5 (50)	9 (81.8)
	Includes Adult Outcomes	10 (21.7)	0 (0)	0 (0)	2 (66.7)	1 (6.3)	5 (50)	2 (18.2)
Data Source	Add Health	3 (6.5)	0 (0)	0 (0)	0 (0)	1 (6.3)	1 (10)	1 (9.1)
	Minnesota Student Survey	6 (13.1)	5 (55.6)	0 (0)	0 (0)	2 (12.5)	0 (0)	0 (0)
	New Mexico Youth Risk and	4 (8.7)	0 (0)	0 (0)	0 (0)	4 (25)	0 (0)	0 (0)

	Resilience Survey							
	YRBS	2 (4.3)	1 (11.1)	0 (0)	0 (0)	1 (6.3)	0 (0)	0 (0)
	Other	21 (45.7)	2 (22.2)	0 (0)	1 (33.3)	6 (37.4)	6 (60)	8 (72.7)
	Not Reported	10 (21.7)	1 (11.1)	1 (100)	2 (66.7)	2 (12.5)	3 (30)	2 (18.2)

File S6. Percent of tests showing a beneficial association between a positive childhood experience and a health outcome

		Substance misuse	Suicidal behaviors	Violence perpetration	Violence victimization	Anxiety	Depression	Post-traumatic stress disorders	Other mental health	Cardiovascular disease C	Other physical health	Any outcome
		% of associations where beneficial relationship detected (Total # of tests, mean sample size studies where beneficial association was detected)										
Relationships	Being in nurturing, supportive relationships (combined measures)	100% (2, 3414)	0% (0, 0)	0% (0,0)	0% (0,0)	0% (0,0)	0% (0, 0)	0% (0,0)	100% (1, 357)	0% (0, 0)	0% (0, 0)	100% (3, 2395)
	Being securely attached to a parent or caregiver	100% (4, 1779)	0% (1, 0)	0% (0,0)	0% (0,0)	0% (0,0)	100% (2, 12248)	0% (0,0)	0% (0, 0)	0% (0, 0)	0% (0, 0)	86% (7, 5269)
	Doing activities with caregiver	0% (0, 0)	0% (0, 0)	0% (0,0)	0% (0,0)	0% (0,0)	100% (1, 12248)	0% (0,0)	0% (0, 0)	0% (0, 0)	0% (0, 0)	100% (1, 12248)
	Familism and related family environments	75% (4, 251)	0% (0, 0)	0% (0,0)	0% (0,0)	0% (2,0)	0% (3, 0)	0% (0,0)	0% (0, 0)	0% (0, 0)	0% (0, 0)	33% (9, 251)
	Having positive peer relationships	10% (10, 2168)	50% (20, 20501)	20% (5, 18451)	50% (4, 9399)	100% (2, 3975)	38% (8, 5914)	50% (2, 96)	80% (5, 34162)	0% (0, 0)	0% (0, 0)	43% (56, 16952)
	Having prosocial peers	70% (10, 2145)	0% (0, 0)	0% (0,0)	0% (0,0)	0% (0,0)	50% (2, 927)	0% (0,0)	0% (0, 0)	0% (0, 0)	0% (0, 0)	67% (12, 1993)
	Love and support from a caregiver	41% (22, 6207)	74% (46, 26440)	89% (9, 15234)	80% (5, 16626)	100% (1, 2617)	65% (20, 26148)	50% (2, 64)	82% (11, 19724)	67% (3, 454)	50% (4, 1930)	67% (123, 20097)
	Quality relationship with caregiver	46% (46, 3600)	71% (14, 8771)	67% (9, 3931)	60% (5, 3467)	80% (5, 419)	79% (19, 12805)	100% (1, 3604)	100% (4, 4416)	0% (0, 0)	100% (1, 340)	62% (104, 6379)
	Social Support	11% (9, 538)	50% (6, 11814)	60% (5, 1909)	100% (3, 1419)	50% (4, 991)	50% (6, 3455)	0% (0,0)	100% (1, 141)	0% (0, 0)	0% (0, 0)	47% (34, 3653)
	Support from a teacher	67% (9, 18441)	17% (6, 11836)	0% (0,0)	0% (0,0)	0% (0,0)	33% (3, 82135)	0% (0,0)	67% (3, 9241)	0% (0, 0)	0% (0, 0)	48% (21, 22310)
	Support from non-caregiver adult (excluding teachers)	64% (11, 1854)	67% (15, 17743)	40% (5, 1520)	67% (3, 1520)	0% (1,0)	17% (6, 396)	0% (0,0)	0% (2, 0)	0% (0, 0)	50% (2, 12270)	53% (45, 9094)
En[illegible]	Access to green spaces and playgrounds to play	0% (0, 0)	0% (0, 0)	0% (0, 0)	0% (0, 0)	100% (1, 762)	100% (1, 762)	0% (0, 0)	0% (0, 0)	0% (0, 0)	0% (1, 0)	67% (3, 762)

	Having access to a protective school environment	81% (36, 10678)	63% (38, 9277)	82% (11, 11392)	75% (4, 16017)	100% (2, 1525)	60% (10, 4231)	0% (0, 0)	100% (6, 9737)	0% (0, 0)	0% (0, 0)	74% (107, 9744)
	Living in a safe neighborhood or community	40% (10, 3530)	0% (2, 0)	0% (2, 0)	0% (0, 0)	0% (0, 0)	0% (2, 0)	0% (0, 0)	100% (1, 6483)	0% (0, 0)	0% (0, 0)	29% (17, 4121)
	Parental monitoring and related family environments	63% (51, 2311)	100% (12, 11076)	75% (13, 2159)	0% (0, 0)	0% (0, 0)	100% (4, 516)	0% (0, 0)	0% (0, 0)	0% (0, 0)	0% (0, 0)	72% (80, 4068)
	Regular physical activity	67% (9, 16343)	36% (14, 14765)	0% (0, 0)	0% (0, 0)	100% (1, 4888)	50% (4, 7389)	0% (0, 0)	100% (1, 14306)	0% (0, 0)	0% (0, 0)	52% (29, 13724)
	Availability of culturally appropriate and effective mental and behavioral health services	0% (1, 0)	100% (1, 2744)	0% (0, 0)	0% (0, 0)	0% (0, 0)	0% (1, 0)	0% (0, 0)	0% (0, 0)	0% (0, 0)	0% (0, 0)	33% (3, 2744)
Engagement	Community connectedness	100% (2, 26536)	100% (8, 40146)	100% (1, 46588)	0% (0, 0)	0% (0, 0)	67% (3, 42152)	0% (0, 0)	100% (2, 4722)	0% (0, 0)	0% (0, 0)	94% (16, 34305)
	For American Indian or Alaska Native communities, native culture engagement	100% (1, 123)	0% (0, 0)	0% (0, 0)	0% (0, 0)	0% (0, 0)	0% (0, 0)	0% (0, 0)	0% (0, 0)	0% (0, 0)	0% (0, 0)	100% (1, 123)
	Having opportunities with constructive social engagement and developing connectedness	100% (2, 450)	0% (0, 0)	0% (0, 0)	0% (0, 0)	0% (0, 0)	0% (1, 0)	0% (0, 0)	100% (1, 129)	0% (0, 0)	0% (0, 0)	75% (4, 343)
	Having opportunity for extracurricular engagement with school or with the community	38% (8, 2681)	50% (8, 29644)	50% (2, 18451)	67% (3, 47021)	0% (0, 0)	80% (5, 37116)	0% (0, 0)	67% (6, 1924)	0% (0, 0)	0% (0, 0)	56% (32, 21960)
	Having opportunity to have beliefs that give comfort	50% (26, 1466)	100% (2, 17143)	0% (0, 0)	0% (0, 0)	0% (0, 0)	50% (2, 14272)	0% (0, 0)	0% (0, 0)	0% (0, 0)	0% (0, 0)	53% (30, 4226)
	Sense of community or other cultural belonging	52% (21, 557)	0% (0, 0)	40% (5, 598)	0% (0, 0)	0% (2, 0)	50% (2, 1466)	0% (0, 0)	0% (1, 0)	0% (0, 0)	0% (0, 0)	45% (31, 628)
	Cumulative measures of PCEs	60% (5, 8186)	100% (1, 206)	0% (0, 0)	0% (0, 0)	0% (1, 0)	100% (5, 87190)	100% (2, 210)	0% (0, 0)	100% (1, 1147)	0% (0, 0)	80% (15, 38523)
	Other	14% (7, 303)	0% (1, 0)	100% (1, 213)	0% (0, 0)	0% (0, 0)	0% (2, 0)	0% (1, 0)	0% (1, 0)	0% (0, 0)	50% (2, 523)	20% (15, 346)
